# Fungicidal Activity and In Silico Studies of Triazoles Derived From Glycerol Against *Neocosmospora falciformis*, A Causal Agent of Guava Tree Decline

**DOI:** 10.1002/cbdv.71389

**Published:** 2026-06-05

**Authors:** Adilson Vidal Costa, Arêssa de Oliveira Correia, Breno Benvindo dos Anjos, Mariana Belizario de Oliveira, Poliana Aparecida Rodrigues Gazolla, Vagner Tebaldi de Queiroz, Juliana Alves Resende, Róbson Ricardo Teixeira, Osmair Vital de Oliveira, Gabriel Jacomin Vargas, Waldir Cintra de Jesus Júnior, Willian Bucker Moraes, Fábio Ramos Alves

**Affiliations:** ^1^ Department of Chemistry and Physics Federal University of Espírito Santo Alegre Espírito Santo Brazil; ^2^ Department of Agronomy Federal University of Espírito Santo Alegre Espírito Santo Brazil; ^3^ Department of Pharmacy and Nutrition Federal University of Espírito Santo Alegre Espírito Santo Brazil; ^4^ Department of Chemistry Federal University of Viçosa Viçosa Minas Gerais Brazil; ^5^ Federal Institute of São Paulo Catanduva São Paulo Brazil; ^6^ Federal University of São Carlos Buri São Paulo Brazil

**Keywords:** antifungal agents, glycerol, guava tree, molecular modeling, *Psidium guajava*

## Abstract

Guava tree decline is a complex disease characterized by root rot caused by the soilborne fungus *Neocosmospora falciformis*. Current management strategies have proven unsatisfactory under field conditions, highlighting the need for research into alternative control methods. This study evaluated the fungicidal activity of seventeen glycerol‐derived triazoles against the mycelial growth and sporulation of *N. falciformis* at concentrations of 1, 10, 100, 500, and 1000 µg/mL. The commercial fungicide tebuconazole was included as a positive control. At 1000 µg/mL, triazole **4l** completely inhibited mycelial growth and eliminated *N. falciformis* spores. Triazoles **4i**–**4l** and **4n**–**4q** also showed satisfactory activity at the highest concentrations in both parameters evaluated. Molecular docking studies investigated the interactions of 1,2,3‐triazole derivatives with a modeled lanosterol 14α‐demethylase (FsCYP51) from *N. falciformis*. All derivatives exhibited favorable binding to the enzyme's active site, with binding energies comparable to those of the natural substrate lanosterol and slightly less favorable than that of tebuconazole. These findings suggest that the triazole derivatives effectively prevent lanosterol from accessing the catalytic pocket of FsCYP51, thereby inhibiting its conversion into ergosterol. Notably, the **4l** derivative exhibited enhanced activity, likely due to its docked pose superimposed on tebuconazole, underscoring this compound as a candidate for fungicide development.

## Graphical abstract

Among seventeen 1,2,3‐triazoles synthetized from glycerol (**4a**–**4q**), compound **4l** completely inhibited mycelial growth and eliminated *N. falciformis* spores at 1,000 µg/mL, exhibiting enhanced antifungalactivity and strong binding within the FsCYP51 active site, which highlights its promise as a lead candidate for antifungal development in both biological and computational evaluations.

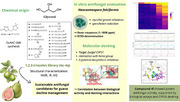

## 1 Introduction

Guava (*Psidium guajava* L.) is a species in the Myrtaceae family, which includes over 80 genera and around 3,000 species, predominantly found in tropical and subtropical regions such as the Americas, Asia, and Australia. The genus *Psidium* comprises roughly 150 species of evergreen shrubs, with *P. guajava* being the most widespread and well‐known globally. Recognized among the top 50 most popular tropical and subtropical edible fruits, guava has significant commercial value in more than 50 countries. Guava fruits are commonly consumed fresh or processed into a variety of products. Leading producers worldwide include India, Pakistan, Sudan, Brazil, Egypt, Mexico, Indonesia, and Bangladesh [1, 2, 3].

In recent years, guava production has been severely affected by a complex disease known as guava tree decline, characterized by root rot, wilting, and eventual plant death. The disease is caused by the soil‐borne fungus *Neocosmospora falciformis* (Carrión) L. Lombard & Crous, which enters plant roots through lesions often induced by the root‐knot nematode *Meloidogyne enterolobii* Yang & Eisenback. This synergistic interaction between nematodes and fungal pathogens has resulted in significant crop losses in guava‐producing regions worldwide [4, 5, 6, 7, 8].

Efforts to manage guava tree decline have focused on nematicides, resistant cultivars, biological control using nematophagous fungi and rhizobacteria, and the incorporation of organic soil amendments. However, these strategies have produced inconsistent and often unsatisfactory results [9, 10, 11]. The continued ineffectiveness of existing control measures has driven the search for new, more effective alternatives.

Fungicides remain essential for crop protection and food security. However, the extensive and prolonged use of synthetic fungicides in agriculture has raised environmental and toxicological concerns and increased the risk of resistance development in target pathogens [12]. Therefore, there is a growing need to discover new fungicidal molecules that are both effective and environmentally sustainable [13].

Triazoles are among the most effective systemic fungicides, widely used in agriculture for their antifungal activity, broad‐spectrum action, rapid uptake, and systemic translocation in plants [14, 15]. Recently, new triazole derivatives with fungicidal activity have been synthesized from glycerol, a major by‐product of biodiesel production. Glycerol is produced in large volumes and poses a significant environmental challenge if improperly disposed of. Therefore, developing sustainable, value‐added applications for surplus glycerol, particularly in agricultural contexts, has become increasingly relevant [16].

Despite numerous management strategies, guava tree decline remains a significant threat to guava production because available control methods are limited in effectiveness and raise environmental concerns about conventional chemical fungicides. While the synthesis and preliminary biological evaluations of glycerol‐derived 1,2,3‐triazoles against fruit‐surface pathogens such as *Colletotrichum gloeosporioides* have been previously reported [17, 18], their potential against complex soilborne diseases remains unexplored. Guava tree decline, caused by *N. falciformis*, represents a distinct and more challenging phytosanitary problem due to its systemic impact on the root system and the lack of effective chemical treatments. Therefore, this study aims to expand the application of these sustainable, glycerol‐based compounds by investigating their fungicidal efficacy specifically against *N. falciformis*. Beyond phenotypic screening, we provide a deeper understanding of the structure‐activity relationship (SAR) for this emergent pathogen and introduce, for the first time, in silico molecular docking studies to elucidate the binding mode of these triazoles with the *N. falciformis* CYP51 enzyme (FsCYP51), aiming to integrate sustainable chemistry with innovative management strategies for guava cultivation.

## 2 Results and Discussion

### 2.1 Preparation of 1,2,3‐Triazole Glycerol‐Derived Compounds

**SCHEME 1:**
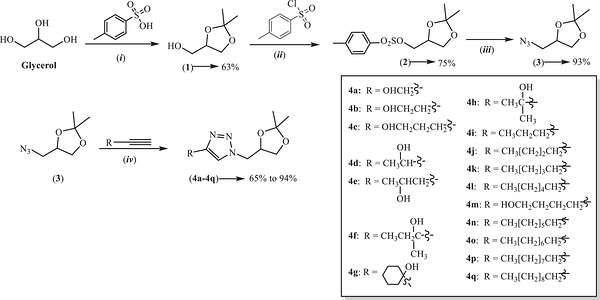
Synthetic route for the preparation of glycerol‐derived 1,2,3‐triazole derivatives. Reagents and conditions: (i) CuSO_4_, acetone, 48 h; (ii) pyridine, 2 h; (iii) NaN_3_, DMF, reflux, 8 h; (iv) sodium ascorbate, CuSO_4_ .5H_2_O, *t*‐butyl alcohol/water (1:1 v v^−1^).

As previously reported, the seventeen 1,2,3‐triazole derivatives (**4a–4q**) used in this study were synthesized via a four‐step route (Scheme 1) [17, 18]. The synthetic route employed glycerol as the starting material, with the copper(I)‐catalyzed azide‐alkyne cycloaddition (CuAAC) reaction serving as the key step for triazole formation [19, 20, 21, 22, 23, 24]. In the first step, glycerol reacted with acetone in the presence of *p*‐toluenesulfonic acid to yield acetal **1** in 63% yield. In the second step, compound **1** reacted with *p*‐toluenesulfonyl chloride to produce ester sulfonate **2** in 75% yield. The third step involved the preparation of organic azide **3** via a bimolecular nucleophilic substitution (S_N_2) reaction between compound **2** and sodium azide, yielding 93% of the product. Finally, the CuAAC reaction between azide **3** and a series of commercially available alkynes yielded seventeen 1,4‐disubstituted 1,2,3‐triazole derivatives (**4a**–**4q**), with isolated yields ranging from 65% to 94%. The structures of the synthesized compounds were confirmed by infrared (IR) and nuclear magnetic resonance (^1^H and ^1^ ^3^C NMR) spectroscopies, followed by mass spectrometry (MS) analysis.

### 2.2 In Vitro Assessment of Mycelial Growth of *N. falciformis* Exposed to Triazole Derivatives

The antifungal activity of seventeen glycerol‐derived triazole compounds **4a**–**4q** against *N. falciformis* isolated from guava trees was evaluated and compared with that of the commercial fungicide tebuconazole. A dose‐dependent inhibitory effect was observed, with higher triazole concentrations producing greater suppression of mycelial growth.

**FIGURE 1:**
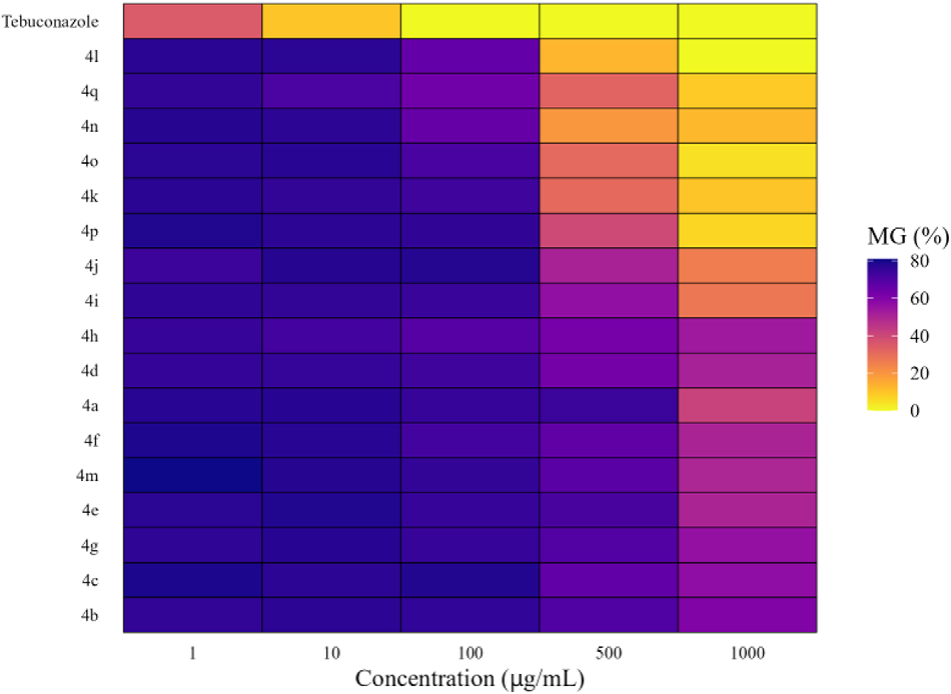
Heatmap of mycelial growth (MG%) of *N. falciformis* UENF/CF 295, isolated from guava tree roots, treated with different concentrations of seventeen glycerol‐derived triazoles **4a**‐**4q**. Color gradient represents the intensity of mycelial growth inhibition, ranging from yellow (high inhibition) to blue (low inhibition).

**FIGURE 2:**
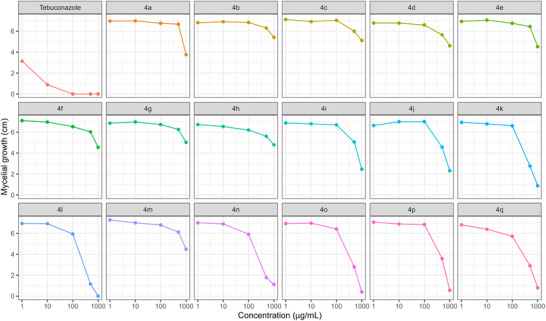
In vitro mycelial growth (cm) of *N. falciformis* UENF/CF 295 exposed to increasing concentrations (1, 10, 100, 500, and 1,000 µg/mL) of seventeen glycerol‐derived triazoles **4a**–**4q**.

At each concentration, statistically distinct groups among the tested compounds were identified using the Scott‐Knott test at a 5% significance level (Table 1). To support and visually represent these findings, a heatmap was used to illustrate the percentage of fungal mycelial growth (Figure 1), and growth curves were used to show variation in mycelial expansion across concentrations (Figure 2).

**TABLE 1:** Diameter of mycelial growth (cm) of *N. falciformis* UENF/CF 295 from guava tree roots after exposure to different concentrations of seventeen glycerol‐derived triazole derivatives.

Compounds	Concentrations (µg/mL)				
	1	10	100	500	1000
**4a**	6.96 a	6.98 a	6.75 a	6.67 a	3.75 d
**4b**	6.81 a	6.90 a	6.83 a	6.30 b	5.41 a
**4c**	7.12 a	6.91 a	7.04 a	5.99 c	5.11 b
**4d**	6.78 a	6.77 a	6.59 b	5.65 d	4.61 c
**4e**	6.93 a	7.06 a	6.75 a	6.44 a	4.52 c
**4f**	7.10 a	6.97 a	6.53 b	6.03 c	4.55 b
**4g**	6.86 a	6.98 a	6.73 a	6.26 b	5.03 b
**4h**	6.73 a	6.54 b	6.20 c	5.61 d	4.79 b
**4i**	6.88 a	6.80 a	6.70 a	5.06 e	2.46 e
**4j**	6.64 a	7.00 a	7.01 a	4.57 f	2.32 e
**4k**	6.94 a	6.80 a	6.61 b	2.77 h	0.88 g
**4l**	6.94 a	6.92 a	5.94 c	1.16 j	0.00 i
**4m**	7.28 a	7.00 a	6.79 a	6.12 b	4.48 c
**4n**	7.00 a	6.90 a	5.92 c	1.77 i	1.12 f
**4o**	6.93 a	6.97 a	6.42 b	2.80 h	0.40 h
**4p**	7.06 a	6.89 a	6.84 a	3.58 g	0.56 g
**4q**	6.81 a	6.39 b	5.72 c	2.90 h	0.80 g
**Tebuconazole**	3.14 b	0.89 c	0.00 d	0.00 k	0.00 i

*Note*: Means in a column that share the same letter do not differ significantly at the 5% level, according to the Scott–Knott test. ANOVA and Figure S81 regarding the in vitro studies are found in the Supporting Information.

The triazoles **4a**–**4q** displayed varying levels of inhibitory activity against the mycelial growth of *N. falciformis* UENF/CF 295, when compared to the commercial fungicide tebuconazole (Table 1; Figures 1 and 2).

At 1 µg/mL, tebuconazole showed the greatest inhibition of mycelial growth, while all triazole treatments clustered into a single statistical group with no significant differences among them (Table 1; Figures 1 and 2). This superior tebuconazole performance was maintained at 10 µg/mL. At this concentration, however, the triazole compounds separated into two groups: compounds **4h** and **4q**, which showed higher inhibition, and a second group comprising the remaining derivatives with lower activity (Table 1).

At 100 µg/mL, tebuconazole completely suppressed mycelial growth, while the triazoles were distributed into three groups with distinct inhibitory profiles: (i) **4a**, **4b**, **4c**, **4e**, **4g**, **4i**, **4j**, **4m**, and **4p**; (ii) **4d**, **4f**, and **4k**; and (iii) **4o**, **4h**, **4l**, **4n**, and **4q** (Table 1).

A more pronounced differentiation among triazoles was observed at 500 µg/mL. At this concentration, tebuconazole again caused complete inhibition, and the triazole derivatives were classified into ten statistical groups. Compounds **4l**, **4n**, **4o**, and **4q** exhibited the strongest antifungal effects, as indicated by the smallest mycelial halo diameters (1.16, 1.77, 2.80, and 2.90 cm, respectively; Table 1; Figures 1 and 2).

At 1000 µg/mL, tebuconazole and compound **4l** formed a single statistical group, both achieving 100% inhibition of mycelial growth. Compound **4o** constituted a second group, whereas compounds **4k**, **4p**, and **4q** formed a third group characterized by fungistatic activity (Table 1).

Overall, triazole derivatives **4k**, **4l**, **4o**, and **4q** displayed the most pronounced antifungal activity at higher concentrations (500 and 1000 µg/mL). In contrast, at lower concentrations (1, 10, and 100 µg/mL), all triazoles were less effective than tebuconazole in inhibiting mycelial growth (Table 1; Figures 1 and 2).

The ED_50_ values (Table 2) were determined because they provide a more accurate quantitative measure of the relative potency of the triazoles against *N. falciformis* than the traditional MIC values.

**TABLE 2:** Effective Dose for 50% growth inhibition (ED_50_, µg/mL) for compounds **4a**–**4q**.

Compounds	ED_50_ (µg/mL)	SE	LCI	UCI
**4a**	1,038.70	40.5	956.4	1,120.90
**4b**	31,773.90	207,719.90	−390,363.80	453,911.60
**4c**	12,606.70	Not determined	Not determined	Not determined
**4d**	1,848.10	572.8	684.1	3,012.10
**4e**	1,252.80	135.9	976.6	1,529.00
**4f**	1,522.50	372.3	765.9	2,279.00
**4g**	1,700.20	473	739	2,661.40
**4h**	10,919.80	Not determined	Not determined	Not determined
**4i**	784.50	48.40	686.10	882.90
**4j**	706.2	47.4	609.8	802.6
**4k**	419.3	38.9	340.2	498.3
**4l**	230.1	26.6	176.1	284.2
**4m**	1,366.30	222.1	914.9	1,817.80
**4n**	277.2	30.4	215.4	339
**4o**	441.7	39.6	361.1	522.2
**4p**	508.8	22.8	462.5	555.1
**4q**	397.9	54	288.2	507.7
**Tebuconazole**	7.47	0.08	7.13	7.80

*Note*: ED: effective dose, SE: standard errors, LCI: lower confidence interval, UCI: Upper confidence interval, not determined: indicate could not be estimated due to insufficient data.

### 2.3 In Vitro Assessment of Sporulation of *N. falciformis* Exposed to Glycerol‐Derived Triazole

The ability of glycerol‐derived triazole compounds (**4a**–**4q**) to suppress the sporulation of *N. falciformis* was evaluated relative to the commercial fungicide tebuconazole. The results showed significant variation in the compounds’ effects on spore production across concentrations, with statistical significance at the 5% level.

For each concentration, distinct statistical groupings were identified using the Scott‐Knott test (p < 0.05) (Table 2). To further illustrate and support these findings, a heatmap showing spore count distribution (Figure 4) and graphs depicting the effect of triazole concentrations on spore production reduction (Figure 5) were generated.

At 1 µg/mL, two distinct groups were identified: (i) tebuconazole and triazoles **4b**, **4d**, **4g**, **4j**, **4l**, **4m**, and **4n**; and (ii) the remaining triazoles (Table 3). At both 10 and 100 µg/mL, four distinct groups emerged. Notably, at 100 µg/mL, only tebuconazole completely inhibited spore production (Table 3).

**TABLE 3:** In vitro evaluation of the average number of spores produced by *N. falciformis* UENF/CF 295, isolated from guava roots and exposed to different concentrations of seventeen glycerol‐derived triazoles.

Triazoles	Concentrations (µg/mL)				
	1	10	100	500	1,000
**4a**	106.51a	63.43 c	88.03 c	78.06 c	49.67 e
**4b**	61.20 b	73.74 c	69.80 c	75.62 c	84.51 d
**4c**	86.30 a	81.88 c	112.06 b	188.29 a	214.9 a
**4d**	58.13 b	103.54 b	117.10 b	84.11 c	89.19 d
**4e**	93.52 a	110.38 b	113.10 b	121.14 b	173.2 b
**4f**	86.27 a	102.31 b	113.27 b	152.94 a	141.12 c
**4g**	64.05 b	51.83 d	93.87 b	96.76 c	118.51 c
**4h**	88.97 a	93.53 b	99.70 b	135.17 b	111.65 c
**4i**	87.96 a	153.26 a	163.46 a	88.63 c	31.96 f
**4j**	59.47 b	126.65 a	58.21 c	84.68 c	70.75 f
**4k**	80.67 a	81.31 c	130.36 b	43.63 d	12.35 f
**4l**	72.76 b	50.97 d	76.57 c	12.06 d	00.00 f
**4m**	69.93 b	83.60 c	66.97 c	158.84 a	65.72 e
**4n**	64.10 b	80.01 c	75.85 c	31.28 d	18.56 f
**4o**	76.93 a	78.98 c	73.82 c	38.77 d	0.51 f
**4p**	81.76 a	49.22 d	61.06 c	48.87 d	2.32 f
**4q**	90.80 a	63.00 c	81.37 c	32.38 d	5.47 f
**Tebuconazole**	31.03 b	16.60 d	00.00 d	00.00 d	00.00 f

*Note*: Means followed by the same letter in each column do not differ significantly according to the Scott–Knott test at the 5% level. ANOVA analysis is available in the Supporting Information.

**FIGURE 3:**
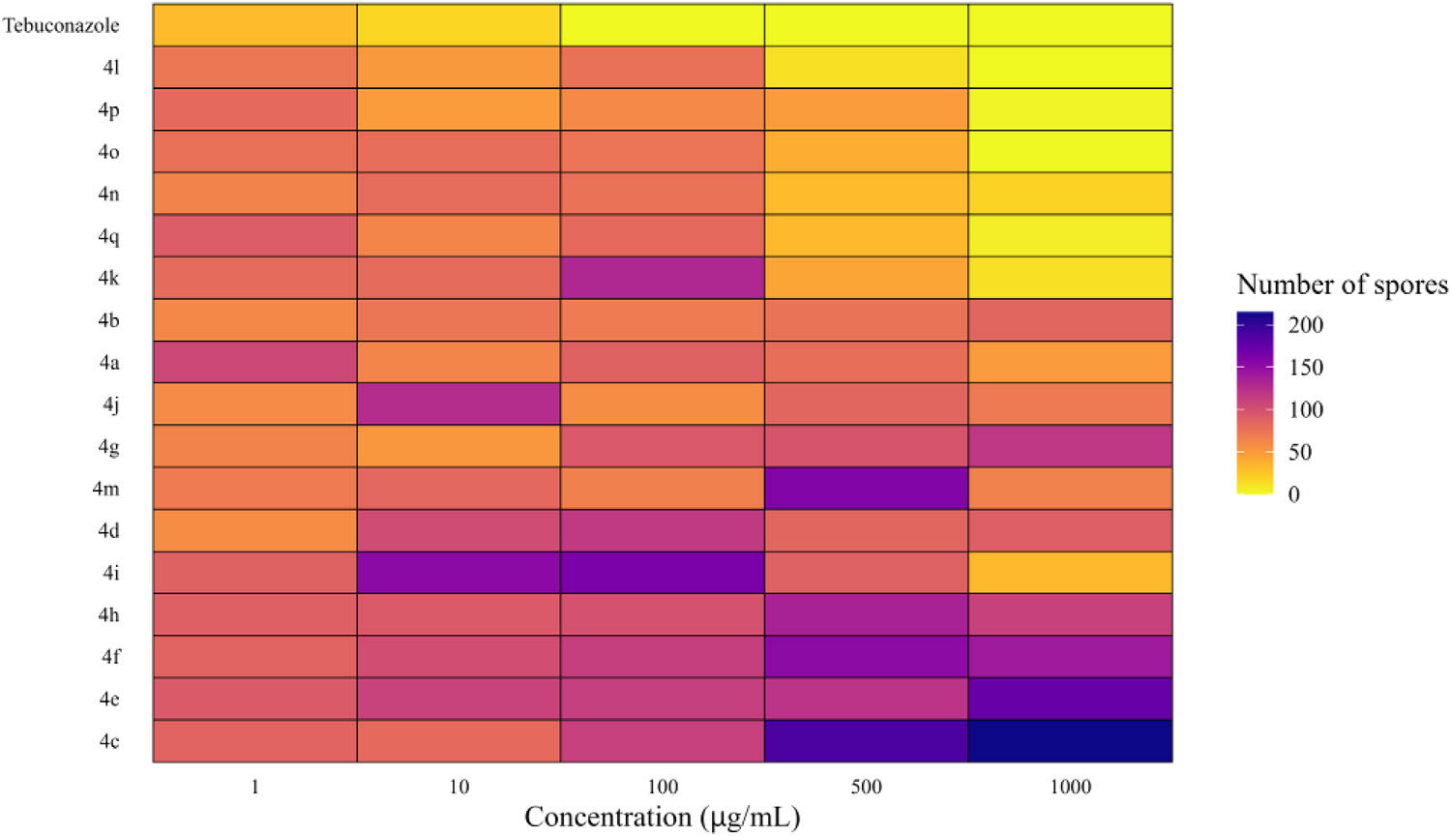
Heatmap of the average spore production by *N. falciformis* UENF/CF 295, isolated from guava roots and treated with different concentrations of seventeen glycerol‐derived triazoles. The color gradient represents the number of spores, ranging from yellow (low) to blue (high inhibition).

At 500 µg/mL, triazoles **4k**, **4l,** and **4n**–**4q** clustered with the commercial fungicide, exhibiting no significant differences in spore inhibition. A similar pattern was observed at 1000 µg/mL, where triazoles **4i–4l** and **4n**–**4q** also grouped with tebuconazole, demonstrating strong efficacy in reducing spore counts (Table 3 and Figure 3).

**FIGURE 4:**
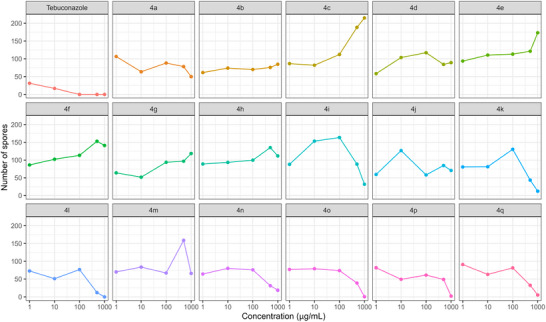
In vitro evaluation of the average spore counts of the *N. falciformis* isolate UENF/CF 295 exposed to increasing concentrations (1, 10, 100, 500, and 1000 µg/mL) of seventeen glycerol‐derived triazoles.

For triazoles **4i–4q**, higher concentrations (500 and 1000 µg/mL) led to greater reductions in spore production, except for triazoles **4j** and **4m** (Figures 3 and 4).

### 2.4 In Silico Study

The docking protocol in this study used was validated by redocking the co‐crystallized ligand, voriconazole, into the active site of the FsCYP51 enzyme. The voriconazole‐FsCYP51 complex generated in this calculation was superimposed with the crystallographic structure of CYP51 (PDB ID 4YUM), and a root mean square deviation (RMSD) of 0.6 Å was obtained between the best‐docked and crystallized ligand voriconazole. This RMSD value is well within the acceptable threshold (<2.0 Å), confirming the reliability of our docking parameters.

A molecular docking study was conducted to elucidate the binding modes of glycerol‐derived triazole compounds (**4a**–**4q**) with the modeled FsCYP51 enzyme. All compounds exhibited favorable binding within the active site of FsCYP51, with calculated binding energies ranging from −6.9 to −8.0 kcal/mol.

**FIGURE 5:**
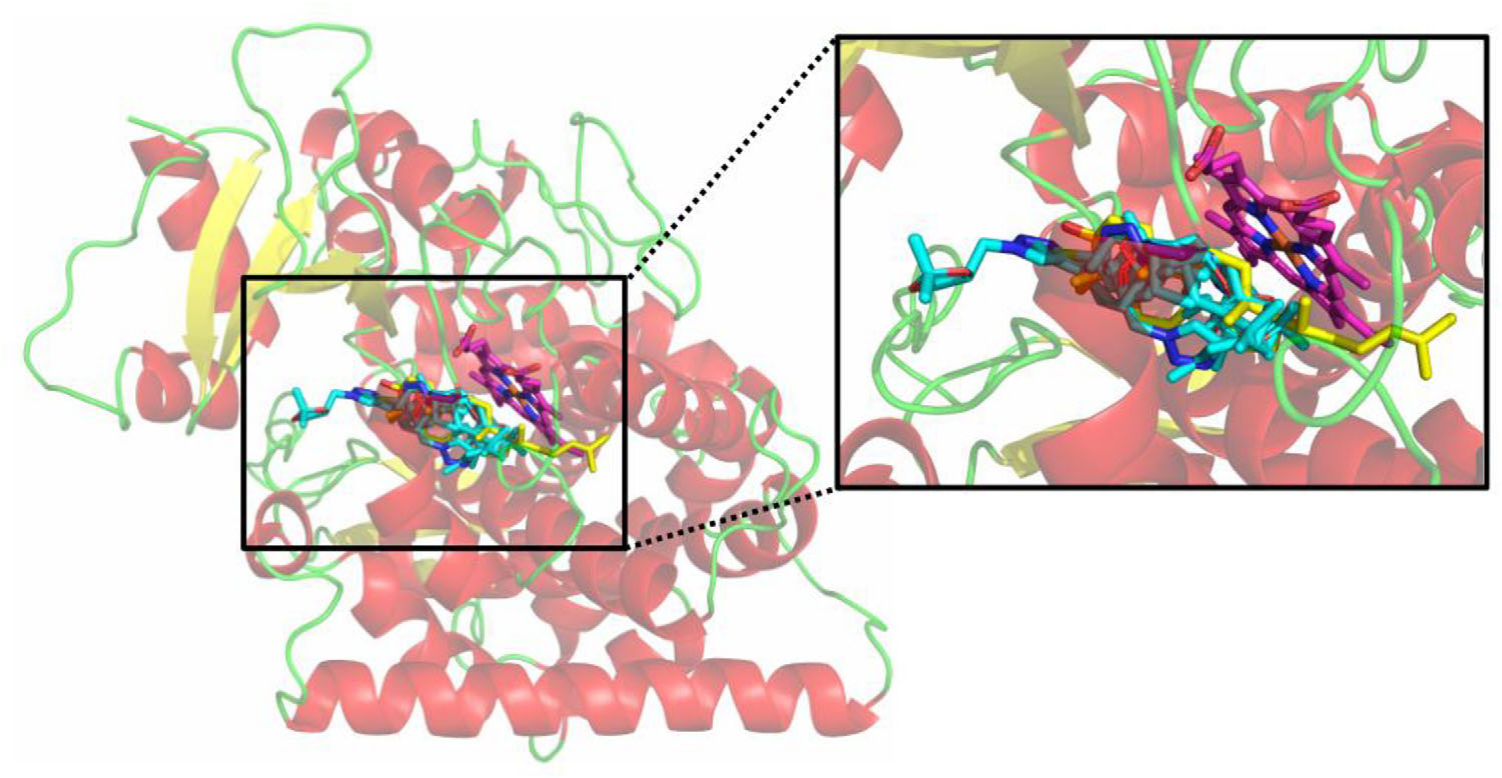
Binding of the selected compounds (in cyan) to FsCYP51. The heme and the substrate, lanosterol, are shown in magenta and yellow, respectively.

Figure 5 shows the best‐docked compounds—**4i**, **4j**, **4k**, **4l**, **4n**, **4p**, and **4q**—complexed with FsCYP51. These compounds were selected based on their strong in vitro fungistatic effects against *N. falciformis* (Table 1). Additionally, docking simulations were performed for lanosterol (LAN) and the commercial fungicide tebuconazole (TEB), which served as reference ligands. All 1,2,3‐triazole derivatives occupied the same binding pocket near the heme cofactor as LAN and TEB.

**FIGURE 6:**
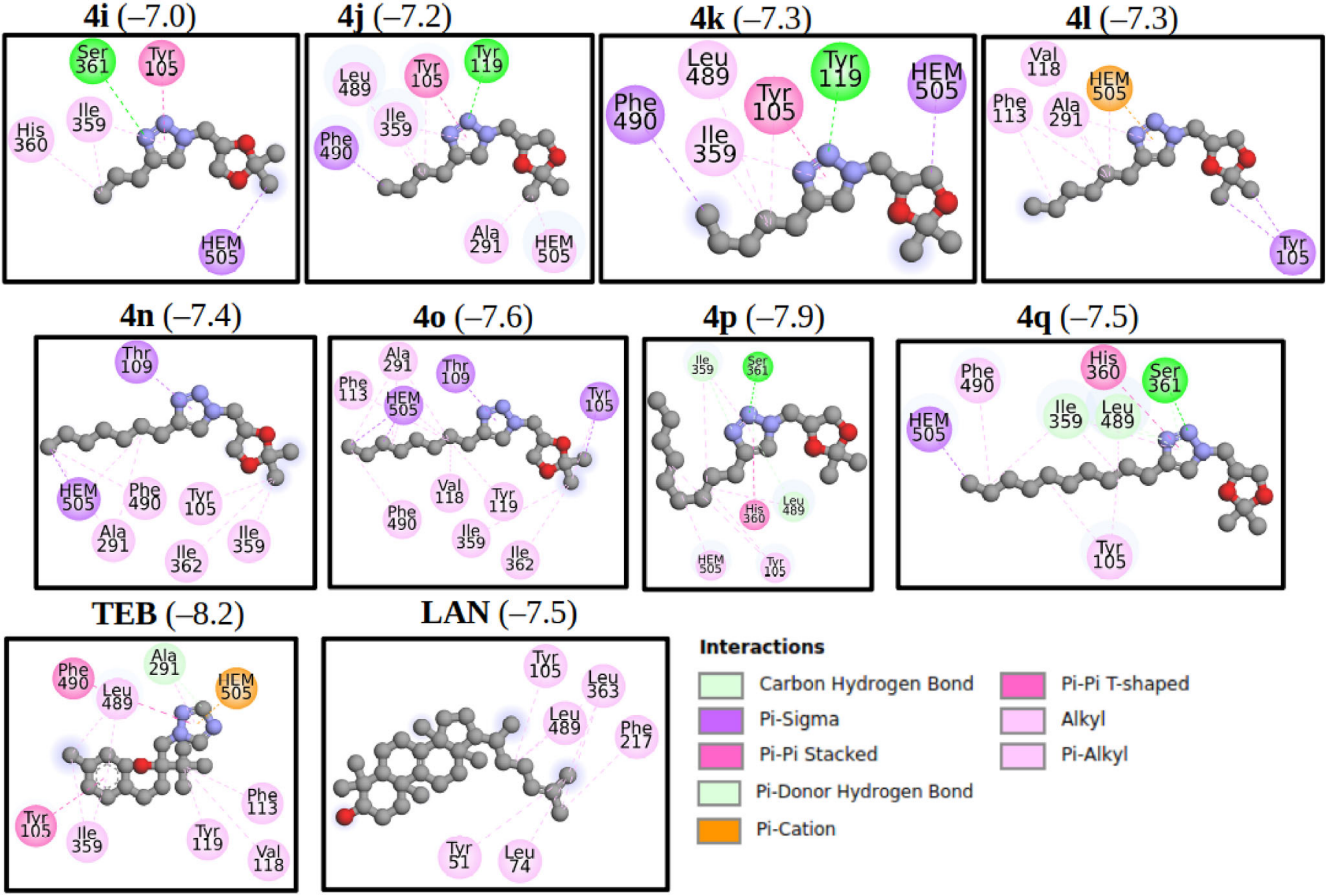
2D interaction map showing the nature of interactions between the selected docked poses with FsCYP51. In parentheses, the binding energy in kcal/mol is given.

**FIGURE 7:**
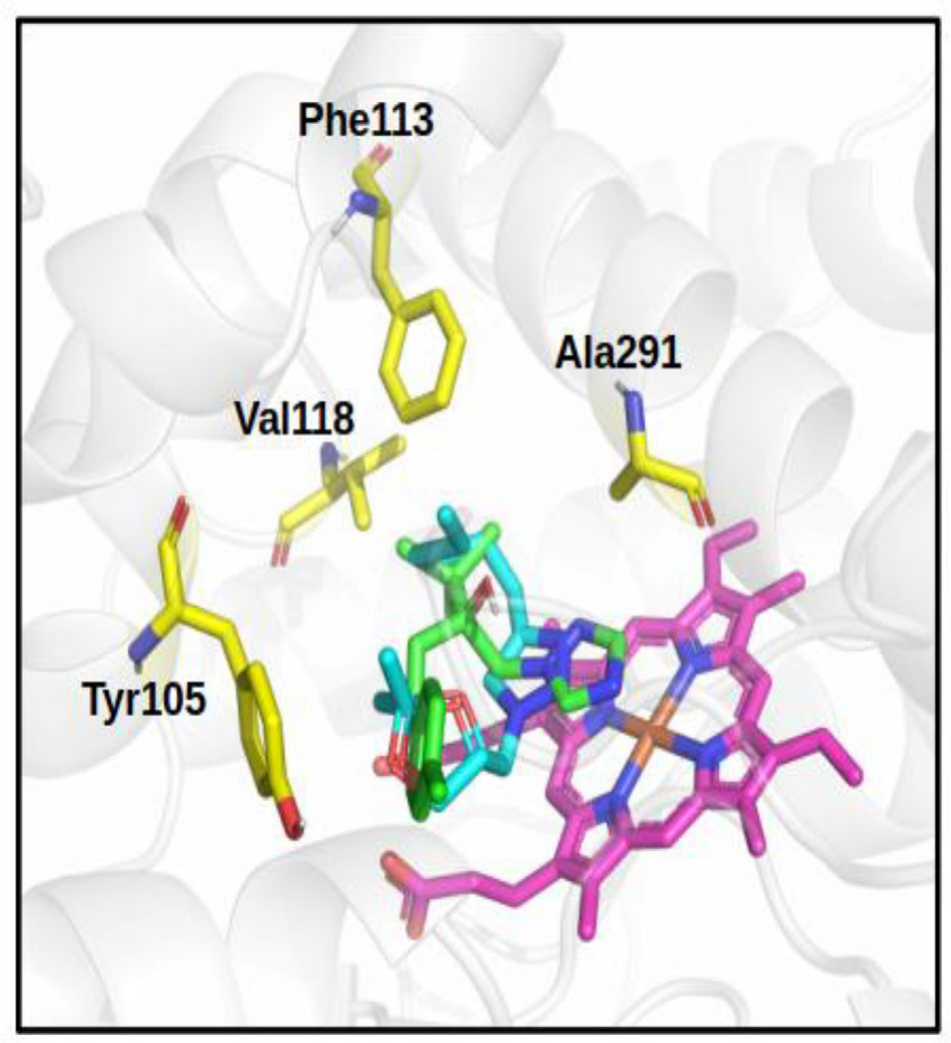
Best‐docked **4l** (green) and the TEB (cyan) into the FsCYP51. Heme group in magenta.

The 2D interaction map (Figure 6) highlights interactions between the selected ligands and FsCYP51. Pi‐Cation interactions with the heme group were observed only in the docked triazole derivatives. Figure 7 shows the binding modes of the best‐docked compound **4l** and TEB within the active site of FsCYP51.

Analysis of the 2D interaction map (Figure 6) shows that the Pi‐Cation interaction with the heme group occurs only in these compounds, suggesting that this interaction is a key contributor to the activity observed in the biological assay. For clarity, Figure 7 shows the binding modes of the best‐docked compounds, **4l** and TEB, within the FsCYP51 enzyme.

Guava is a commercially and nutritionally important crop cultivated in many countries. However, guava orchards, particularly in Brazil, have been severely affected by guava decline, a complex disease characterized by root parasitism by the root‐knot nematode *Meloidogyne enterolobii* and secondary infections caused by opportunistic fungi such as *N. falciformis*. Managing this disease remains challenging, necessitating the development of new, effective control strategies. In this context, we evaluated seventeen 1,2,3‐triazole‐glycerol derivatives (**4a–4q**) for their fungicidal activity against *N. falciformis*.

The antifungal activity of the compounds was assessed by their ability to inhibit mycelial growth of *N. falciformis*. The results showed dose‐dependent inhibition, with more pronounced effects at higher concentrations (Table 1). Notably, compound **4l** completely inhibited mycelial growth at 1,000 µg/mL, making it the most effective among the tested compounds. Compounds **4k**, **4n**, **4o**, **4p**, and **4q** also exhibited substantial inhibition at this concentration.

The fungicidal activity of triazole compounds is primarily due to inhibition of ergosterol biosynthesis, a key component of fungal plasma membranes. Triazoles target lanosterol demethylase, thereby preventing ergosterol production. The resulting membrane disruption compromises fungal cell viability [15, 25, 26]. This mode of action may explain the inhibitory effects observed in our study.

Several studies support the antifungal activity of triazoles against *Neocosmospora* species. For instance, Rodrigues reported complete inhibition of *N. falciformis* mycelial growth by tebuconazole at 1,000 µg a.i./mL [27]. Similarly, Fisher et al. observed complete suppression of *Fusarium subglutinans* growth with tebuconazole at 100 and 1,000 µg/mL [28]. Other studies have highlighted the fungistatic effects of triazoles against *F. graminearum*, *F. meridionale*, and *F. oxysporum*, as well as the inhibitory activity of triticonazole and triflumizole against *F. graminearum* [29, 30, 31].

In addition to affecting mycelial growth, triazole derivatives significantly alter the fungus´s sporulation capacity. In our study, compound **4l** completely suppressed spore production of *N. falciformis* at 1,000 µg/mL. This finding is significant, given the role of spores as the primary propagules of fungal reproduction. Inhibiting sporulation is particularly relevant, as fungal spores serve as the primary inoculum for disease propagation under field conditions. Therefore, suppressing conidial formation may reduce epidemic onset and limit pathogen dissemination through soil or pruning equipment. Other triazoles have similarly shown inhibitory effects on sporulation in various fungal species, including *Cylindrocladium candelabrum*, *Sclerotinia sclerotiorum*, *Alternaria ricini*, *Melampsora medusae*, *Colletotrichum gloeosporioides*, *Oidium eucalypti*, and *A. solani* [32, 33, 34, 35, 36, 37, 38]. However, sporulation inhibition is not universal; for instance, *Myrothecium roridum* and *S. sclerotiorum* were not affected by certain triazoles [39, 40].

Triazole activity can vary across fungal species. Silva, for example, evaluated nine novel triazole derivatives against both human and plant pathogenic fungi, including *Candida* spp. and *Cryptococcus* spp., and reported no significant antifungal effect at optimal concentrations [16]. These performance variations may be attributed to compound‐specific interactions with fungal enzymes and cellular targets.

Mechanistically, triazoles inhibit lanosterol demethylation, leading to the accumulation of 14α‐methyl sterol intermediates. This disruption of ergosterol biosynthesis compromises membrane integrity and impairs fungal viability [41, 42, 43]. Despite their potent activity, triazoles are considered environmentally safe when applied at recommended doses. Roman et al. report that soil microorganisms can degrade these compounds, using them as carbon sources [15, 17]. Klink et al. further emphasize the importance for triazoles in the effective control of fungal diseases [44].

Because fungal spores play an ecological role similar to that of seeds, suppressing them is crucial to limiting pathogen spread [45]. The findings of the present study thus have practical implications for the integrated management of guava decline, which involves not only *M. enterolobii* but also *N. falciformis*. To validate the efficacy of these triazole derivatives under real‐world conditions, in vivo studies should be expanded to include greenhouse trials, small‐scale field experiments, and eventually, large‐scale applications.

In addition, the sustainability of these compounds is noteworthy. The triazole derivatives evaluated here are synthesized from glycerol, a biodiesel byproduct. Finding high‐value uses for glycerol help mitigate its environmental impact, including potential soil and water contamination from improper disposal.

It is widely recognized that azole‐based compounds exert antifungal activity by inhibiting lanosterol 14α‐demethylase (CYP51), an essential enzyme in the ergosterol biosynthesis pathway [46, 47]. Consistent with this mechanism, Wang et al. recently demonstrated, using chemical proteomics and chemicobiological approaches, that a 1,2,3‐triazole derivative interacts with CYP51 in *Colletotrichum gloeosporioides* infecting mango [48]. Several studies have reported targeting this enzyme in the search for new antifungal agents [49, 50, 51, 52, 53, 54, 55].

Thus, to better understand the molecular basis of the antifungal activity of the glycerol‐derived 1,2,3‐triazoles (**4a**–**4q**), molecular docking studies were performed using a homology‐modeled structure of the FsCYP51 enzyme from *N. falciformis*. The docking results suggest that the synthesized triazoles, particularly **4l,** may act by inhibiting the FsCYP51 enzyme, thereby preventing the natural substrate lanosterol (LAN) from accessing the enzyme's active site. Binding energy calculations showed that LAN (−7.5 kcal/mol) and most of the derivatives have similar affinities, suggesting competitive inhibition. Notably, tebuconazole (TEB) exhibited a higher binding energy (−8.5 kcal/mol), which correlates with its superior fungistatic effect (Table 1).

Interestingly, although compounds (**4a**–**4h)** interacted favorably with FsCYP51, they showed limited biological activity. This may be due to their small molecular size, which fails to effectively block the substrate entrance channel, allowing the compounds to be easily displaced. These smaller derivatives typically position their 1,2,3‐triazole and 1,3‐dioxolane rings near the heme group, while the hydroxyl group remains distal.

In contrast, compounds **4i**–**4q** feature longer alkyl chains that extend into the substrate‐access channel and interact with key residues. For instance, compounds **4i**, **4j**, **4k**, and **4m** engage the heme cofactor through the 1,3‐dioxolane ring, whereas **4n**–**4q** interact primarily through the alkyl chain. These largely hydrophobic interactions mirror those observed for LAN and TEB. Additionally, regarding the alkyl‐substituted series (**4i**–**4q**), a clear parabolic trend related to the chain length was observed. The antifungal activity increased from methyl (**4i**) to hexyl (**4l**), which reached the maximum inhibition. Further increasing the chain length to decyl (**4q**) decreased activity. This suggests that the hexyl group provides an ideal balance of lipophilicity and molecular volume, enabling efficient membrane permeation and a precise fit within the hydrophobic pocket of the FsCYP51 enzyme.

Docking analysis (Figure 7) showed that residues such as Tyr105, Leu108, Thr109, Phe113, and Ser361 participate in key interactions with the derivatives. Notably, compounds **4i**, **4j**, **4k**, **4p**, and **4q** form hydrogen bonds with Tyr199 or Ser361. These interactions occur within the enzyme's catalytic pocket, which includes critical residues such as Tyr105, Phe113, Thr287, and Phe490. Among the tested compounds, **4l** exhibited the strongest fungicidal activity, comparable to that of TEB at 1,000 µg/mL. The aforementioned findings emphasize the importance of molecular size, functional group orientation, and hydrophobicity in determining antifungal potency among the triazole derivatives investigated in this study.

Further insights were derived from the 2D interaction maps and structural alignment (Figures 6 and 7). Compound **4l** closely aligns with TEB, with its 1,3‐dioxolane and alkyl chains superimposing on TEB's 4‐chlorophenyl and dimethyl ethyl fragments, respectively. Both compounds orient their triazole rings (1,2,3‐triazole in **4l** and 1,2,4‐triazole in TEB) in a parallel conformation relative to the heme group, thereby facilitating a key Pi‐Cation interaction, a likely determinant of their high biological activity.

Taken together, the results of this study highlight the potential of glycerol‐derived 1,2,3‐triazole compounds, particularly **4l**, **4o, 4p**, and **4q**, as candidates for antifungal development against *N. falciformis*, a key pathogen in guava decline. The observed in vitro inhibition of mycelial growth and sporulation, together with molecular docking data indicating favorable interactions with the FsCYP51 catalytic site, support their candidacy for further development. Notably, compound **4l** shares structural and functional features closely aligned with tebuconazole, suggesting a comparable mode of action. The sustainable origin of these compounds from biodiesel byproduct glycerol further enhances their value within integrated disease management strategies. However, in vivo studies, field evaluations, and toxicity assessments are necessary to confirm their applicability under real‐world agricultural conditions and to assess potential risks associated with environmental persistence and the development of resistance.

## 3 Conclusions

Among the seventeen glycerol‐derived 1,2,3‐triazole compounds evaluated, derivative **4l** completely inhibited both mycelial growth and sporulation of *N. falciformis* (UENF/CF 295) at 1,000 µg/mL. Other derivatives, particularly **4i**–**4l** and **4n**–**4q**, also showed substantial inhibition at higher concentrations. Molecular docking analysis indicated that all compounds bound favorably to the FsCYP51 active site, with compound **4l** exhibiting interaction patterns similar to tebuconazole, supporting its pronounced antifungal activity.

Their dual ability to inhibit both mycelial development and sporulation, together with evidence of strong molecular interactions with the FsCYP51 active site, highlights their potential as novel crop protection agents. Furthermore, their sustainable origin in biodiesel‐derived glycerol strengthens their applicability in integrated disease management.

Although compound **4l** is less potent than tebuconazole at lower concentrations, its sustainable origin from glycerol and its possible mechanism of action make it an excellent candidate for future structural optimization toward highly effective, eco‐friendly fungicides.

Future structure‐activity relationship (SAR) studies will be essential to optimize this novel 1,2,3‐triazole scaffold, aiming to enhance its binding affinity for the CYP51 enzyme and significantly lower the effective dose required for field applications.

## 4 Experimental Section

### 4.1 Synthesis of Glycerol Derivatives

Detailed synthetic protocols and purification procedures for all glycerol‐derived triazoles (**4a**–**4q**) are provided in Figures S1–S80. In addition, the spectroscopic data for all compounds are presented, including nuclear magnetic resonance (NMR), infrared (IR), and mass spectrometry (MS) analyses used for their characterization.

### 4.2 Biological Assays

The in vitro assay was conducted using an 18 × 5 factorial design, comprising 18 triazole compounds **4a**–**4q** and five concentration levels (1, 10, 100, 500, and 1,000 µg/mL), with a completely randomized design and five replicates. Each replicate consisted of a Petri dish containing potato dextrose agar (PDA) medium supplemented with the corresponding triazole concentration. The antifungal activity of the compounds was evaluated by assessing their effects on mycelial growth and spore production of the fungus *N. falciformis* isolate UENF/CF 295. For comparison, the commercial fungicide tebuconazole served as a reference.

The *N. falciformis* isolate utilized in the assays was previously identified using specific taxonomic keys and provided by Professor Dr. Ricardo Moreira de Souza of the Universidade Estadual do Norte Fluminense Darcy Ribeiro (UENF). The isolate was maintained on PDA medium in a BOD‐type incubator at 25°C.

### 4.3 In Vitro Assessment of Mycelial Growth and Sporulation of *N. falciformis* Under Triazole Exposure

The antifungal activity of the triazole compounds was evaluated following the method described by Edgington et al. with modifications by Menten et al. and Rampersad and Teelucksingh [56, 57, 58]. Each compound was initially dissolved in 5 mL of dimethyl sulfoxide (DMSO), and the final volume was adjusted to 100 mL with sterile distilled water, yielding a stock solution at 100,000 µg/mL (active ingredient).

Serial dilutions were prepared to achieve final concentrations of 1, 10, 100, 500, and 1000 µg/mL in the culture medium. Under aseptic conditions, 1 mL of each diluted solution was added to 99 mL of molten potato dextrose agar (PDA) at 45°C–50°C, and thoroughly homogenized. The treated medium was then poured into sterile 8 cm diameter Petri dishes.

After solidification, 4‐mm agar plugs containing actively growing fungal mycelium were excised with a sterilized platinum loop and placed at the center of each treated plate. Plates were incubated at 25°C ± 1°C under a 12‐h photoperiod for 7 days. Radial growth was assessed 24 h after inoculation. Colony diameters were measured along two perpendicular axes, and the mean was calculated. The original inoculum plug diameter (4 mm) was subtracted from the colony diameter to obtain the corrected colony diameter, which was recorded for subsequent analysis.

After evaluating mycelial growth, sporulation was assessed on the same plates. For each treatment, an aqueous spore suspension was prepared by adding 10 mL of sterile distilled water directly onto the colony surface. The colony was gently scraped with a Drigalski loop to release spores into the liquid. The resulting suspension was filtered through a double layer of sterile gauze in a glass funnel to retain hyphae and debris. The spore‐containing filtrate was homogenized, and the spore count was quantified using a Neubauer counting chamber, following Cruz et al. [59]. For each replicate, sixteen fields were counted.

### 4.4 Statistical Analysis

For the assessment of antifungal activity, the collected data were analyzed using analysis of variance (ANOVA). The qualitative factor (triazole compound) was evaluated using the Scott‐Knott test at the 5% significance level. For the quantitative factors, several visualizations were generated: a heatmap showing the percentage of mycelial growth and spore counts of *N. falciformis*; line graphs illustrating mycelial growth (cm) and spore production across increasing triazole concentrations; box plots showing the effects of triazole concentrations on mycelial growth and sporulation; and a comparative graph of EC_50_ values (EC_50_< 500 µg/mL) for the tested triazoles and the positive control (tebuconazole) with respect to their effects on mycelial growth. All statistical analyses and graphical representations were performed using R software, version 4.5.0.

### 4.5 Molecular Docking

The 3D homology model of lanosterol 14α‐demethylase (FsCYP51) from *N. falciformis*, previously developed by Oliveira et al. served as the biological receptor in the docking calculations [60]. The compounds **4a–4q,** synthesized by Costa et al. were used as ligands [17, 18]. Their initial structures were drawn and pre‐optimized using Avogadro software [61]. Subsequently, structural optimization was performed using the MOPAC2016 package, with the semi‐empirical PM7 Hamiltonian [62, 63]. The optimized structures were converted from PDB to PDBQT format using the OBABEL program [64]. The active site region of the FsCYP51 enzyme was selected as the target for evaluating the binding modes of the derivatives **4a–4q**. Accordingly, a grid box with dimensions of 28 × 28 × 28 Å and a spacing of 1 Å, centered at coordinates 5.675, −2.998, and −3.003, was defined for the docking calculations. A total of 20 binding modes were generated, and an exhaustiveness level of 16. All molecular docking calculations were performed using the AutoDock Vina package [65]. The results were visualized and analyzed using Discovery Studio Visualizer [66] and PyMOL software [67].

## Author Contributions

**Arêssa de Oliveira Correia, Breno Benvindo dos Anjos, Mariana Belizario de Oliveira, Poliana Aparecida Rodrigues Gazolla, Vagner Tebaldi de Queiroz, Juliana Alves Resende, Gabriel Jacomin Vargas, Waldir Cintra de Jesus Júnior, Willian Bucker Moraes,** and **Fábio Ramos Alves**: data curation, formal analysis, investigation, validation, visualization, and writing – review and editing. **Osmair Vital de Oliveira**: data curation, formal analysis, funding acquisition, investigation, validation, visualization, writing – original draft, and writing – review and editing. **Adilson Vidal Costa** and **Róbson Ricardo Teixeira**: conceptualization, data curation, formal analysis, funding acquisition, investigation, project administration, resources, validation, supervision, visualization, writing – original draft, and writing – review and editing.

## Conflicts of Interest

The authors declare no conflicts of interest.

## Supporting information




**Supporting File 1**: cbdv71389‐sup‐0001‐SuppMat.docx.

## Data Availability

Data sharing not applicable to this article as no datasets were generated or analyzed during the current study.
