# Supplementary material for: Fungicidal Activity and In Silico Studies of Triazoles Derived From Glycerol Against Neocosmospora falciformis, A Causal Agent of Guava Tree Decline
Source: Chem Biodivers. 2026 Jun 5;23(6):e71389. doi: 10.1002/cbdv.71389 (PMC13238394; doi:10.1002/cbdv.71389)
Supplement: Supplementary file 1 — Supporting File 1: cbdv71389‐sup‐0001‐SuppMat.docx. [file CBDV-23-e71389-s001.docx]

Supplementary Information

Fungicidal Activity and *In silico* Studies of Triazoles Derived From Glycerol Against *Neocosmospora falciformis*, a Causal Agent of Guava Tree Decline

Adilson Vidal Costa,^1*^ Arêssa de Oliveira Correia,^2^ Breno Benvindo dos Anjos,^2^ Mariana Belizario de Oliveira,^1^ Poliana Aparecida Rodrigues Gazolla,^1^ Vagner Tebaldi de Queiroz,^1^ Juliana Alves Resende,^3^ Róbson Ricardo Teixeira,^4*^ Osmair Vital de Oliveira,^5^ Gabriel Jacomin Vargas,^4^ Waldir Cintra de Jesus Júnior,^6^ Willian Bucker Moraes,^2^ and Fábio Ramos Alves^2^

^1^Department of Chemistry and Physics, Federal University of Espírito Santo, Alegre, Espírito Santo, Brazil.

^2^Department of Agronomy, Federal University of Espírito Santo, Alegre, Espírito Santo, Brazil.

^3^Department of Pharmacy and Nutrition, Federal University of Espírito Santo, Alegre, Espírito Santo, Brazil.

^4^Department of Chemistry, Federal University of Viçosa, Viçosa, Minas Gerais, Brazil.

^5^Federal Institute of São Paulo, Catanduva Campus, Catanduva, São Paulo, Brazil.

^6^Federal University of São Carlos, Lagoa do Sino Campus, Buri, São Paulo, Brazil.

*Corresponding authors: e-mail: [avcosta@hotmail.com](mailto:avcosta@hotmail.com); [robsonr.teixeira@ufv.br](mailto:robsonr.teixeira@ufv.br)

**1. IR, NMR (^1^H AND ^13^C), AND MS SPECTRA**

**
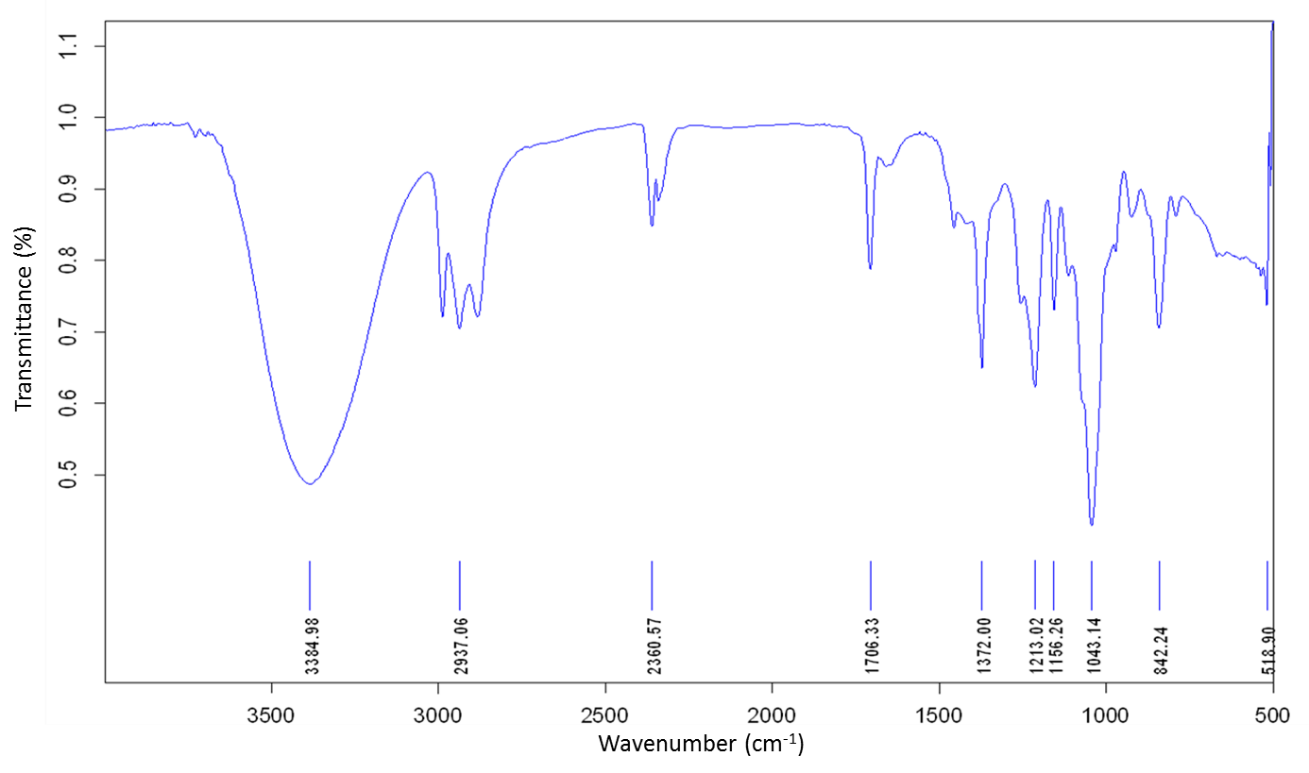
**

**Figure S1.** IR spectrum of (2,2-dimethyl-1,3-dioxolan-4-yl)methanol (**1**).

**
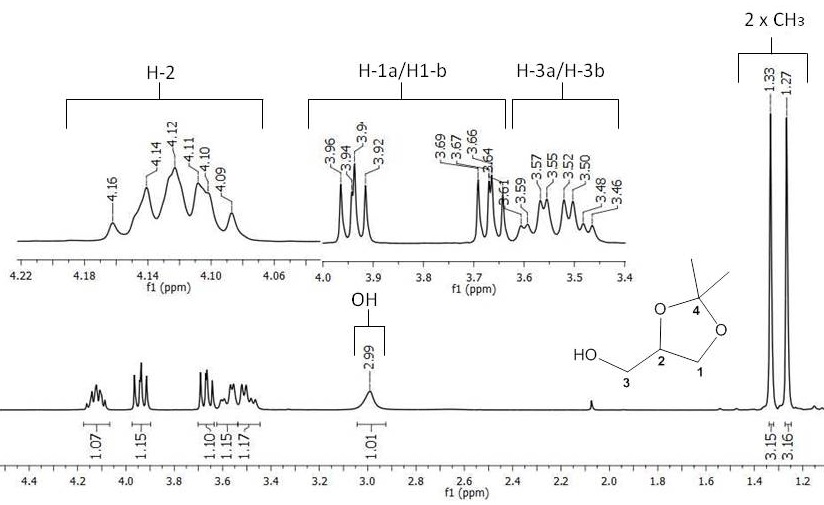
**

**Figure S2.** ^1^H NMR spectrum (300 MHz, CDCl_3_) of (2,2-dimethyl-1,3-dioxolan-4-yl)methanol (**1**).


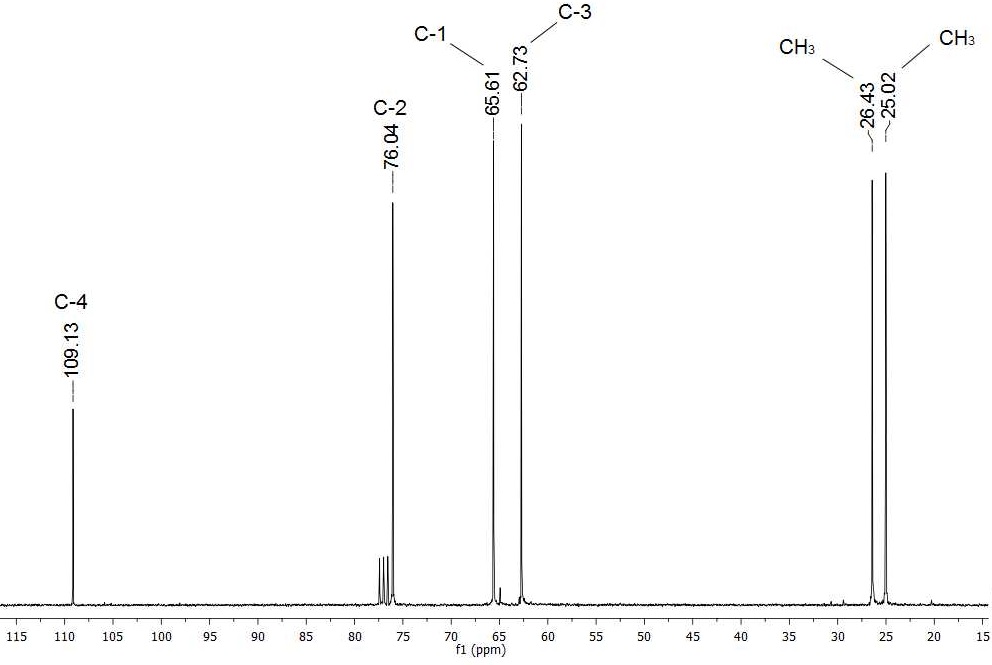


**Figure S3.** ^13^C NMR spectrum (75 MHz, CDCl_3_) of (2,2-dimethyl-1,3-dioxolan-4-yl)methanol (**1**).


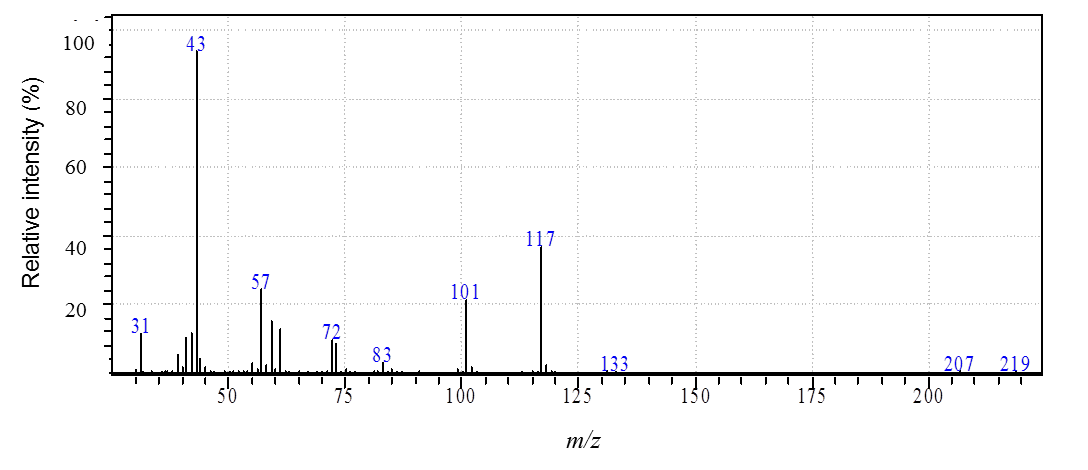


**Figure S4.** MS spectrum of (2,2-dimethyl-1,3-dioxolan-4-yl)methanol (**1**).


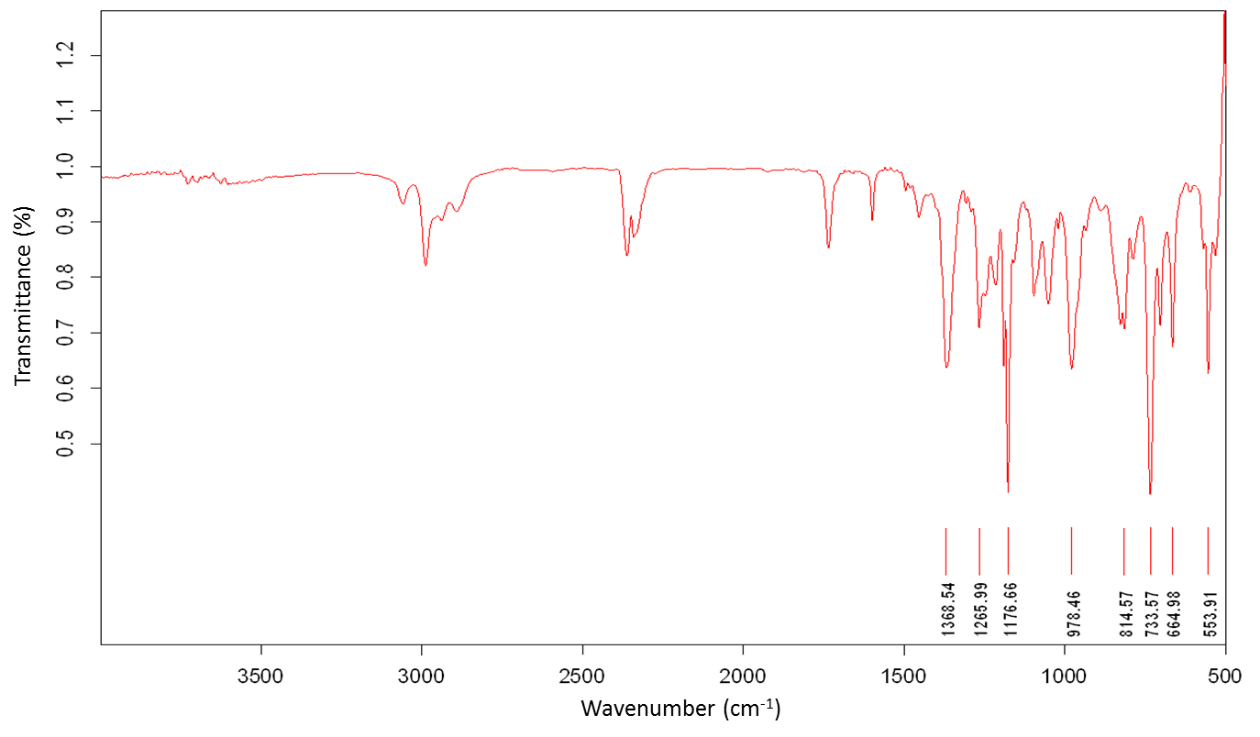


**Figure S5.** IR spectrum of (2,2-dimethyl-1,3-dioxolan-4-yl)methyl-4-methylbenzenesulfonate (**2**).

**
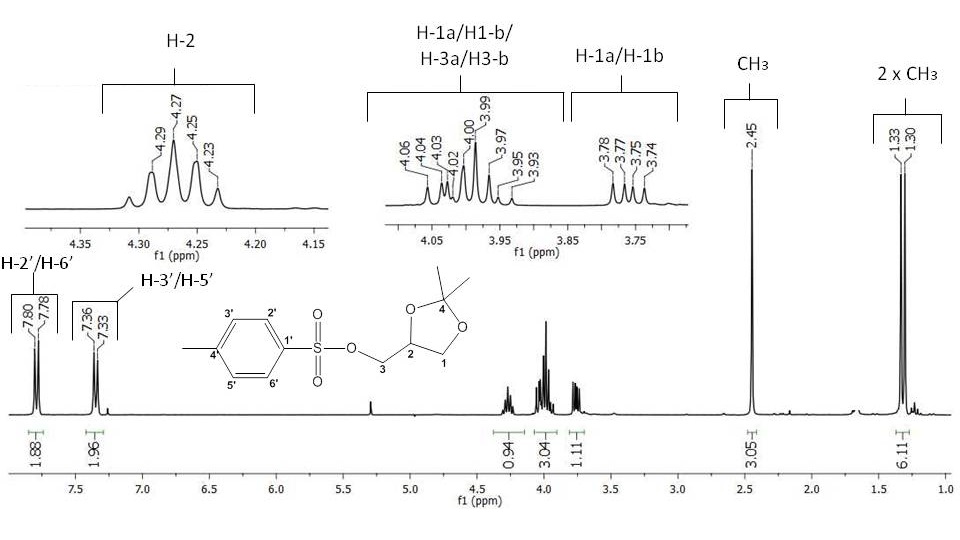
**

**Figure S6.** ^1^H NMR spectrum (300 MHz, CDCl_3_) of (2,2-dimethyl-1,3-dioxolan-4-yl)methyl-4-methylbenzenesulfonate (**2**).


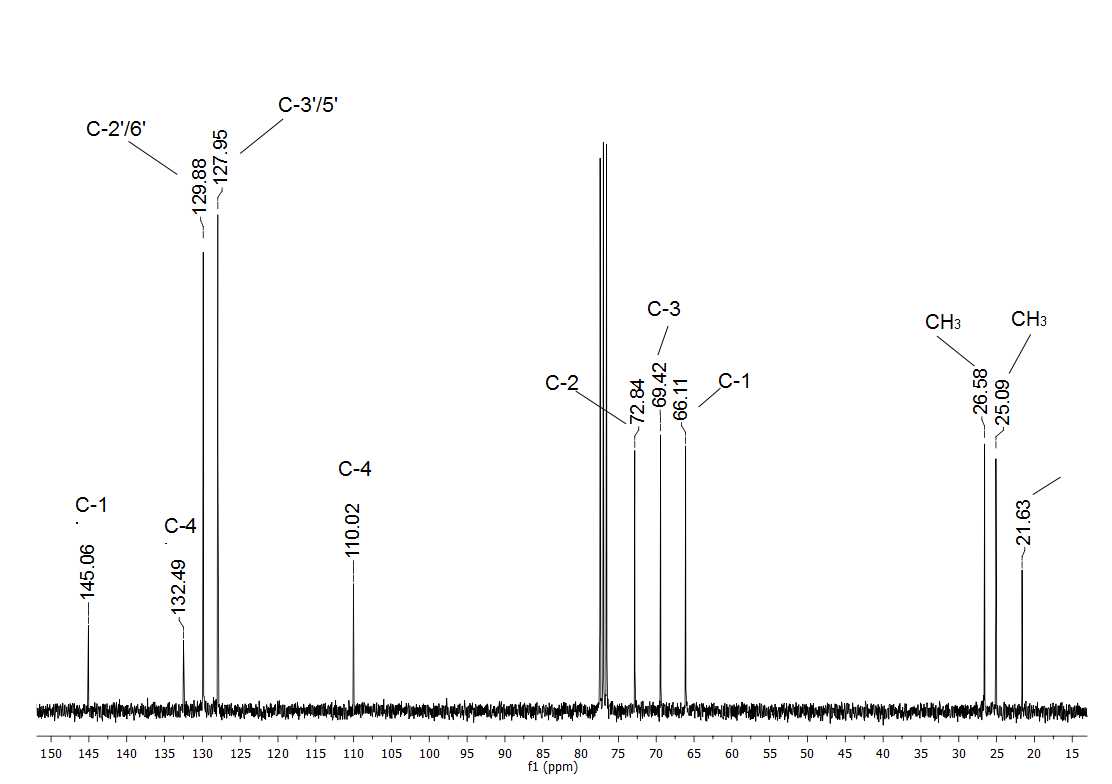


**Figure S7.** ^13^C NMR spectrum (75 MHz, CDCl_3_) of (2,2-dimethyl-1,3-dioxolan-4-yl)methyl-4-methylbenzenesulfonate (**2**).


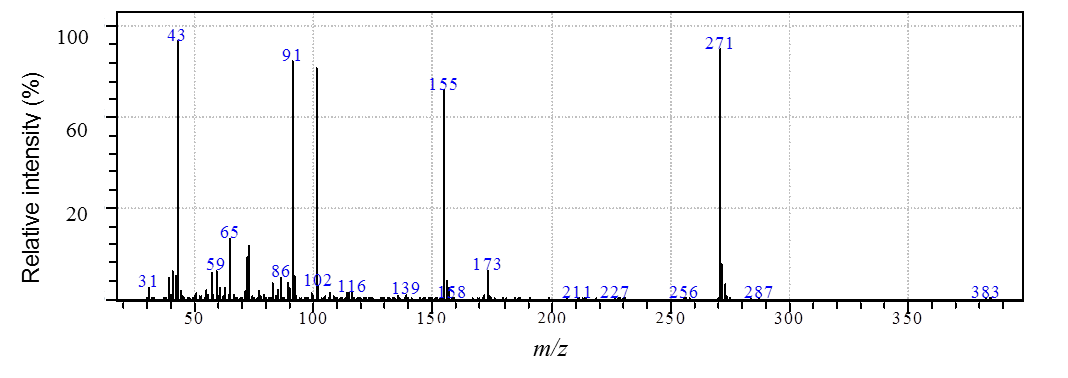


**Figure S8.** MS spectrum of (2,2-dimethyl-1,3-dioxolan-4-yl)methyl-4-methylbenzenesulfonate (**2**).


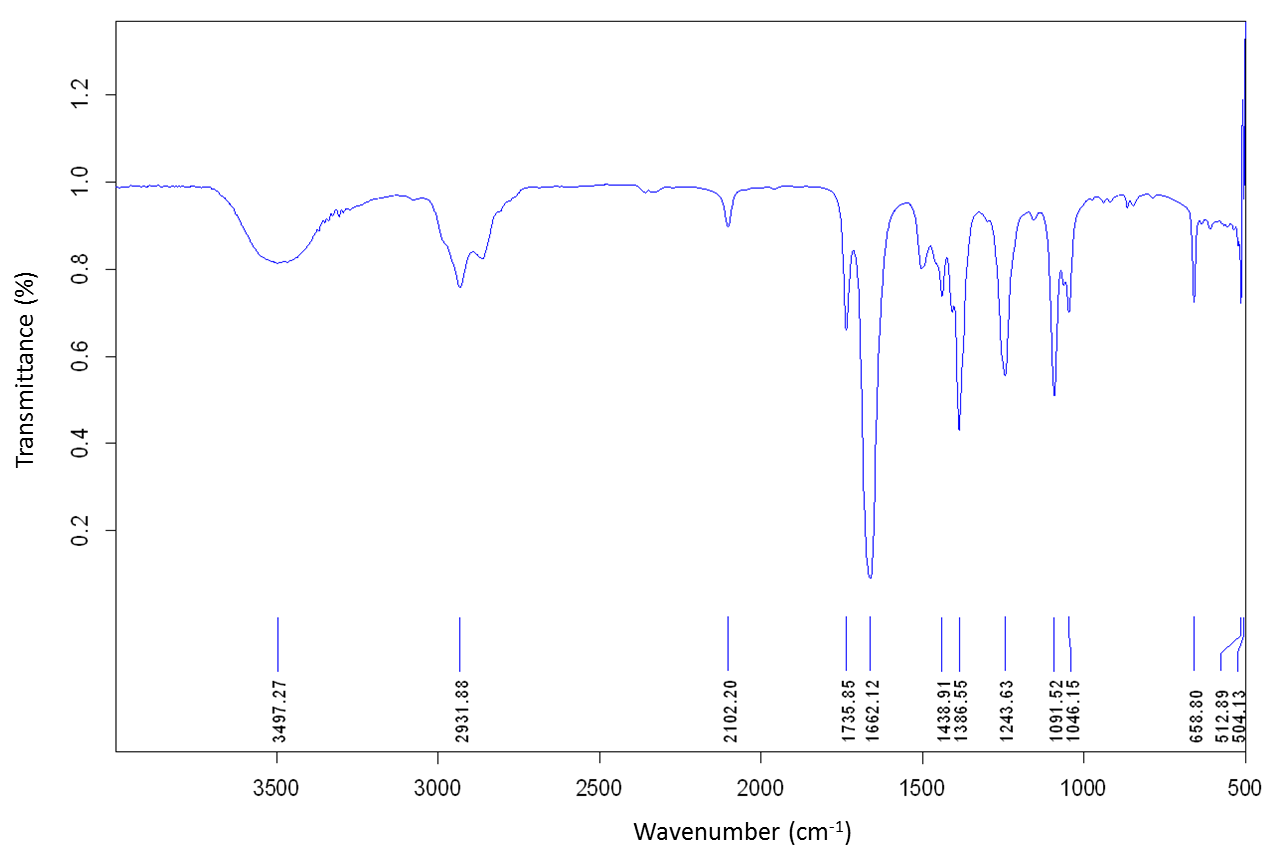


**Figure S9.** IR spectrum of 4-(azidomethyl)-2,2-dimethyl-1,3-dioxolane (**3**).


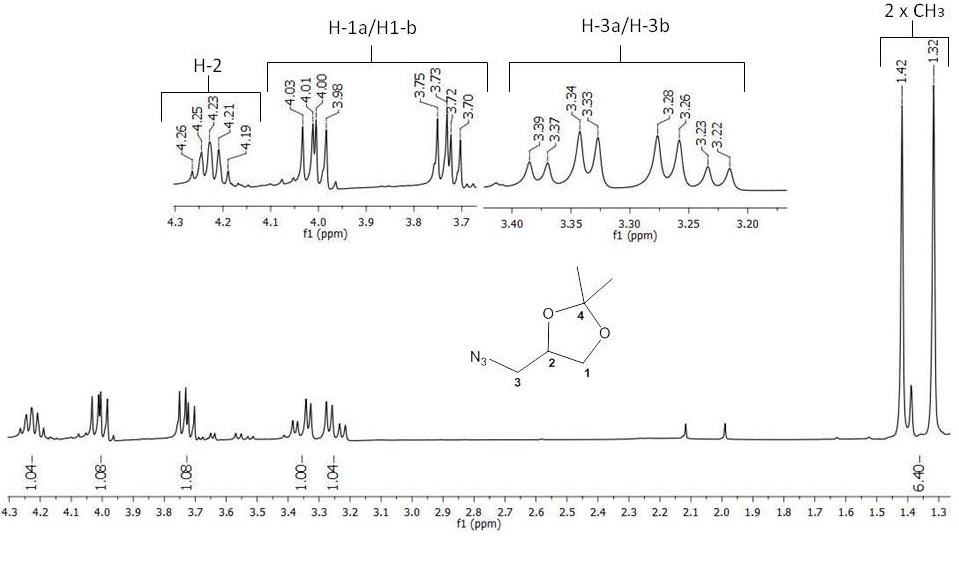


**Figure S10.** ^1^H NMR spectrum (300 MHz, CDCl_3_) of 4-(azidomethyl)-2,2-dimethyl-1,3-dioxolane (**3**).


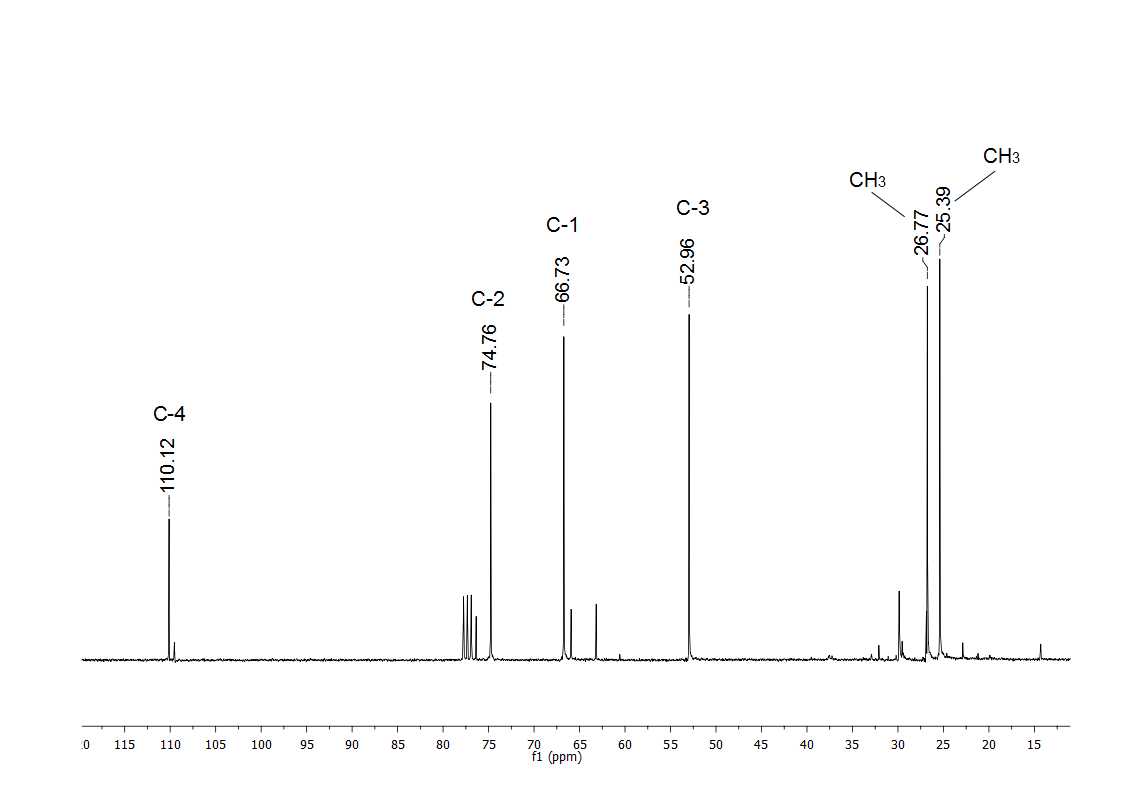


**Figure S11.** ^13^C NMR spectrum (75 MHz, CDCl_3_) of 4-(azidomethyl)-2,2-dimethyl-1,3-dioxolane (**3**).


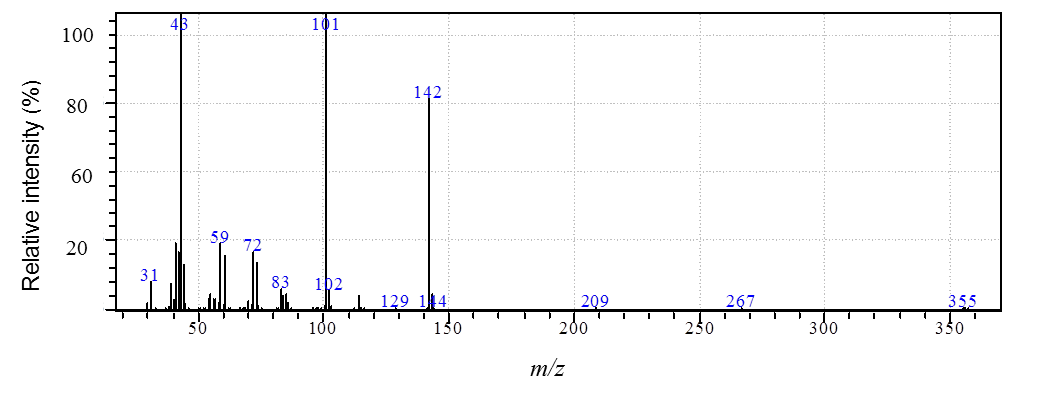


**Figure S12.** MS spectrum of 4-(azidomethyl)-2,2-dimethyl-1,3-dioxolane (**3**).


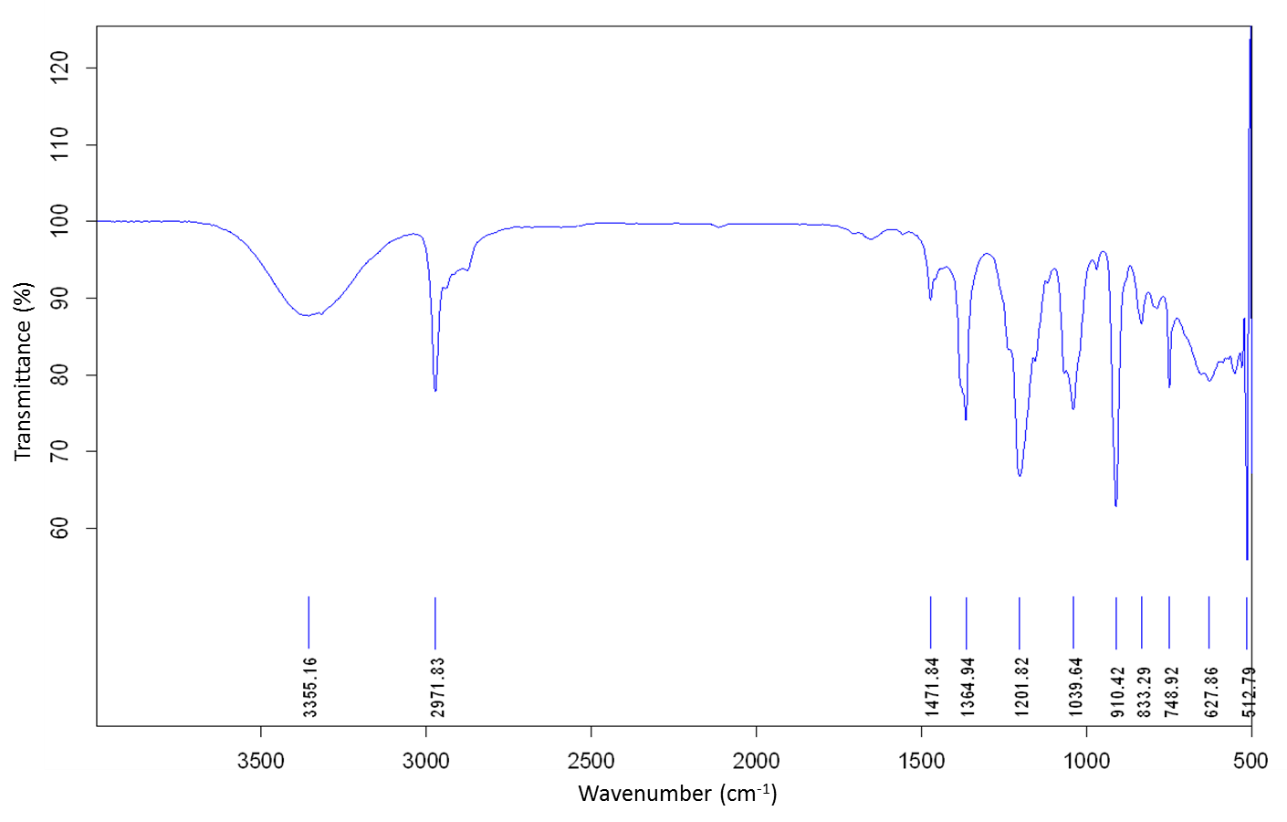


**Figure S13.** IR spectrum of (1-((2,2-dimethyl-1,3-dioxolan-4-yl)methyl)-1*H*-1,2,3-triazol-4-yl)methanol (**4a**).


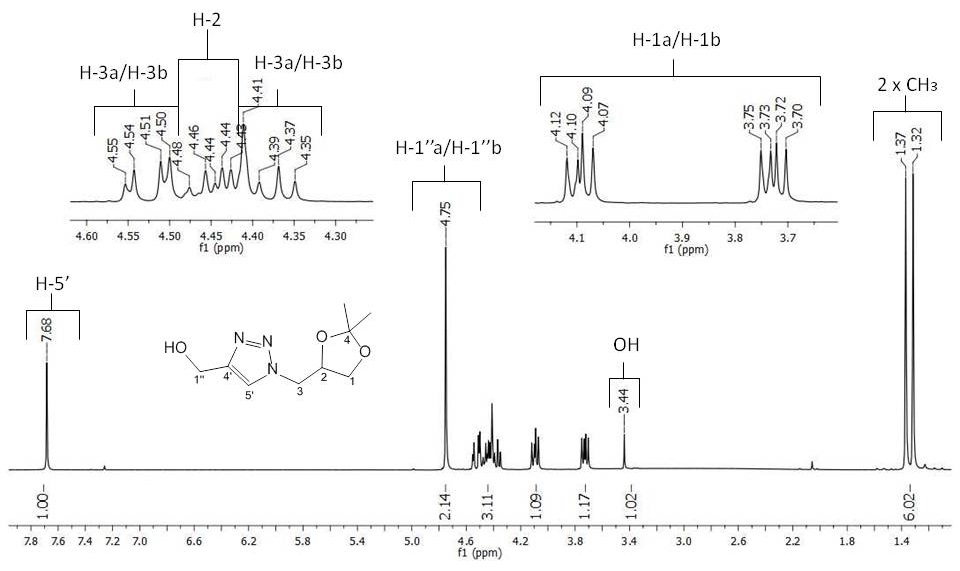


**Figure S14.** ^1^H NMR spectrum (300 MHz, CDCl_3_) of (1-((2,2-dimethyl-1,3-dioxolan-4-yl)methyl)-1*H*-1,2,3-triazol-4-yl)methanol (**4a**).

**
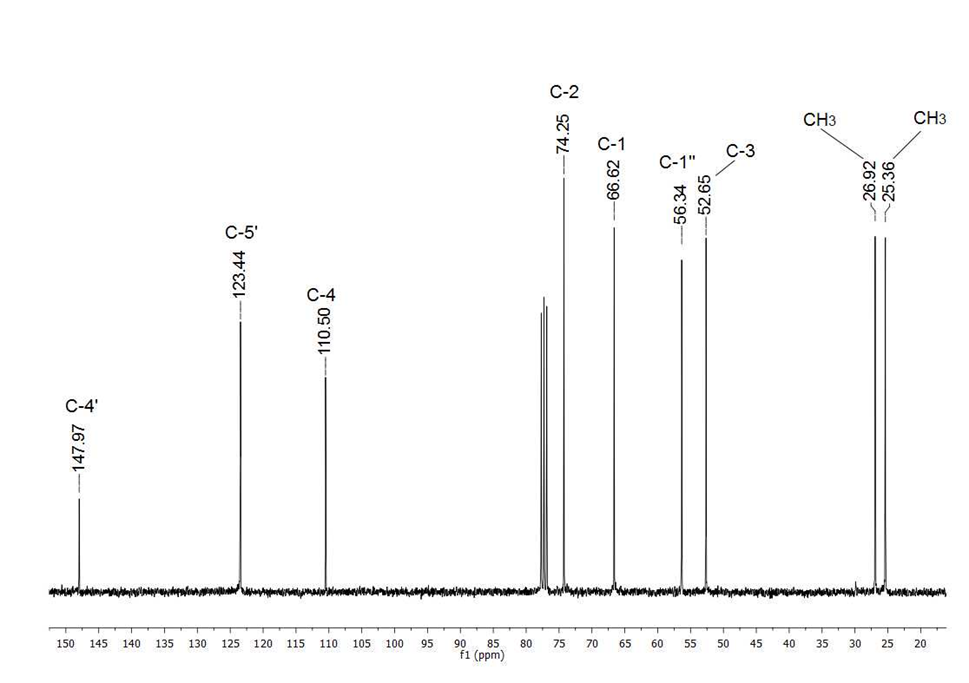
**

**Figure S15.** ^13^C NMR spectrum (75 MHz, CDCl_3_) of (1-((2,2-dimethyl-1,3-dioxolan-4-yl)methyl)-1*H*-1,2,3-triazol-4-yl)methanol (**4a**).


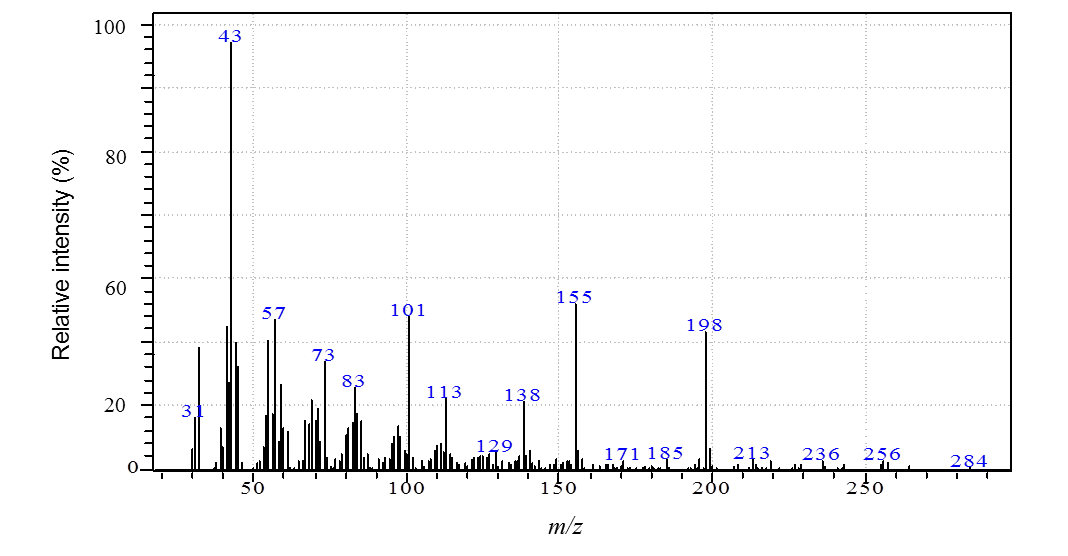


**Figure S16.** MS spectrum of (1-((2,2-dimethyl-1,3-dioxolan-4-yl)methyl)-1*H*-1,2,3-triazol-4-yl)methanol (**4a**).


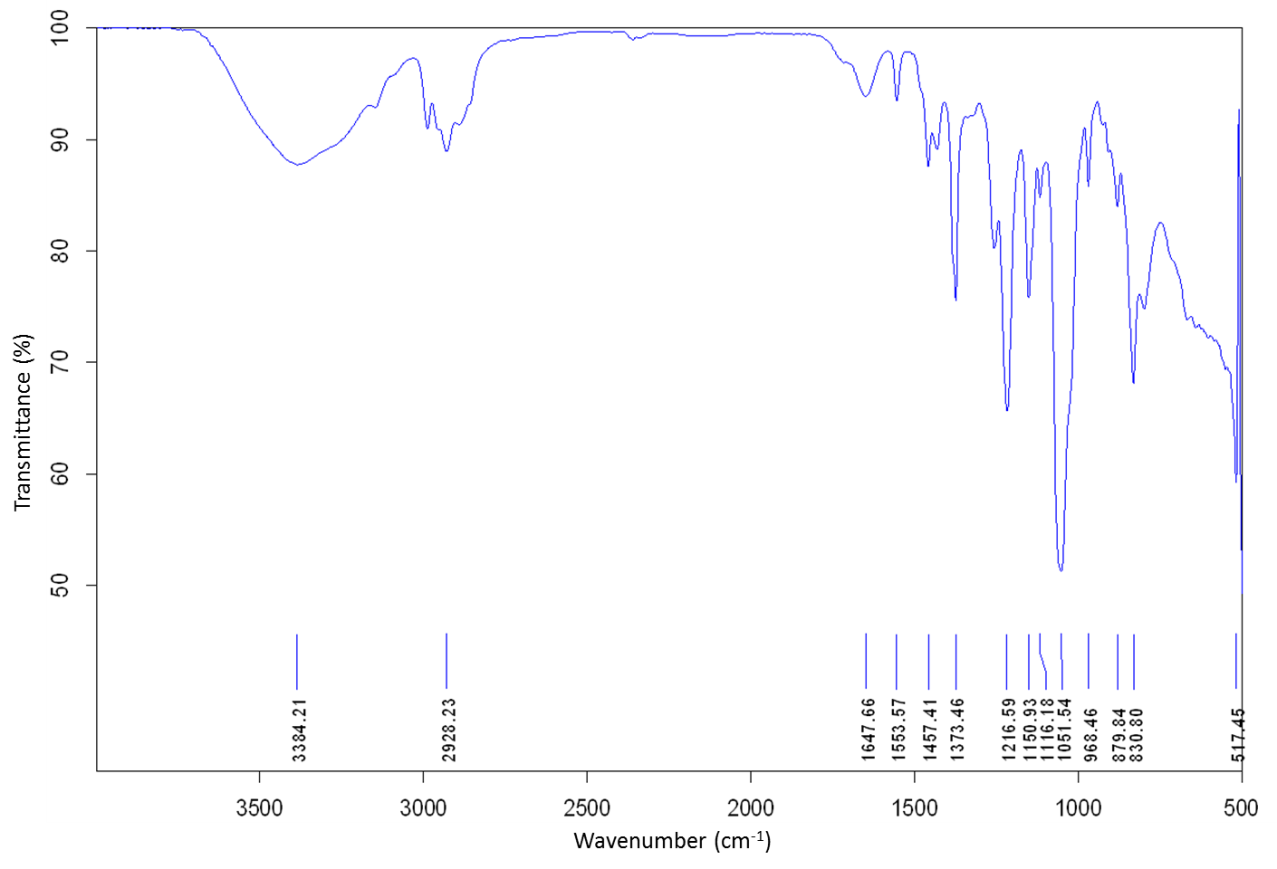


**Figure S17.** IR spectrum of 2-(1-((2,2-dimethyl-1,3-dioxolan-4-yl)methyl)-1*H*-1,2,3-triazol-4-yl)ethanol (**4b**).


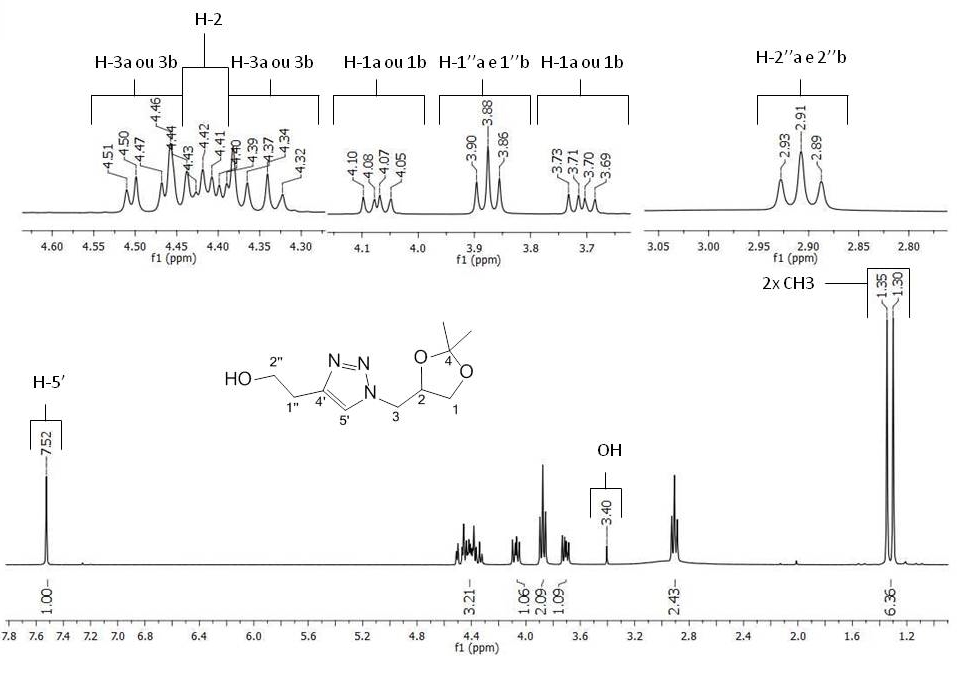


**Figure S18.** ^1^H NMR spectrum (300 MHz, CDCl_3_) of 2-(1-((2,2-dimethyl-1,3-dioxolan-4-yl)methyl)-1*H*-1,2,3-triazol-4-yl)ethanol (**4b**).


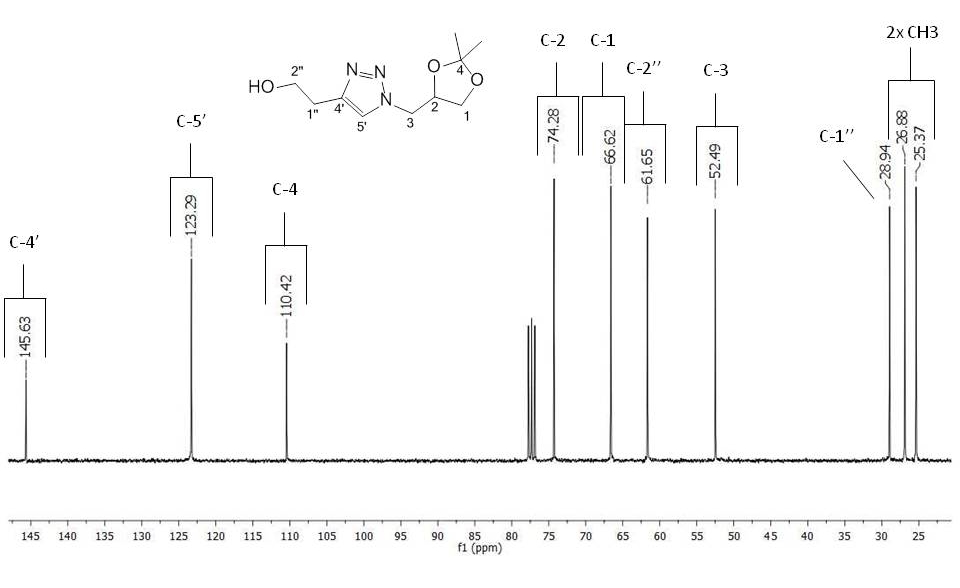


**Figure S19.** ^13^C NMR spectrum (75 MHz, CDCl_3_) of 2-(1-((2,2-dimethyl-1,3-dioxolan-4-yl)methyl)-1*H*-1,2,3-triazol-4-yl)ethanol (**4b**).


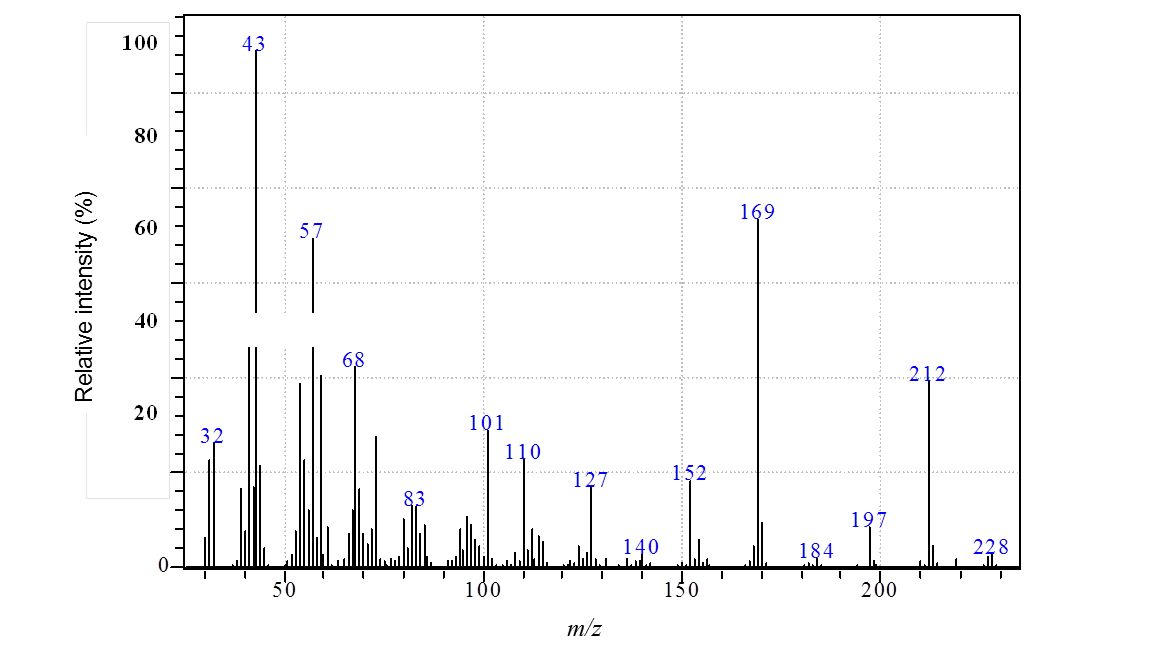


**Figure S20.** MS spectrum of 2-(1-((2,2-dimethyl-1,3-dioxolan-4-yl)methyl)-1*H*-1,2,3-triazol-4-yl)ethanol (**4b**).

**
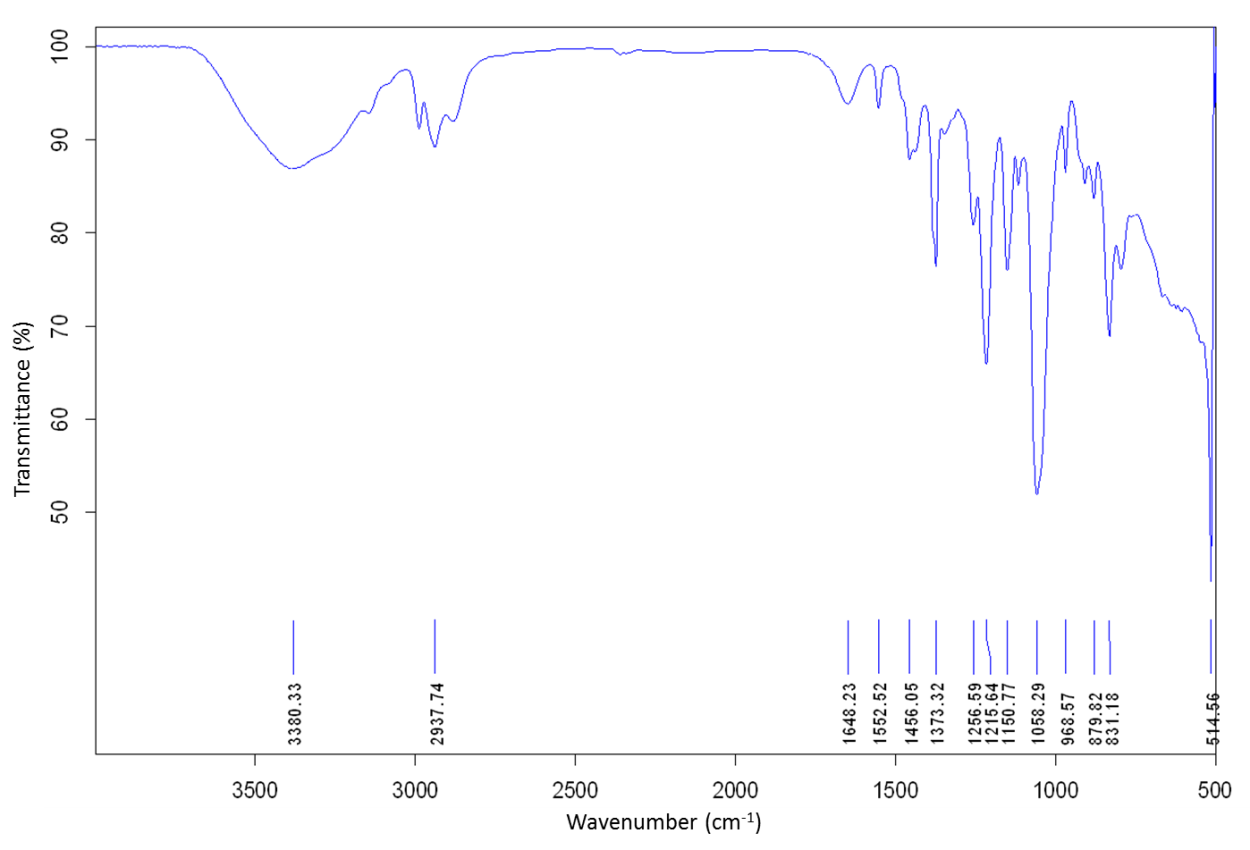
**

**Figure S21.** IR spectrum of 3-(1-((2,2-dimethyl-1,3-dioxolan-4-yl)methyl)-1*H*-1,2,3-triazol-4-yl)propan-1-ol (**4c**).


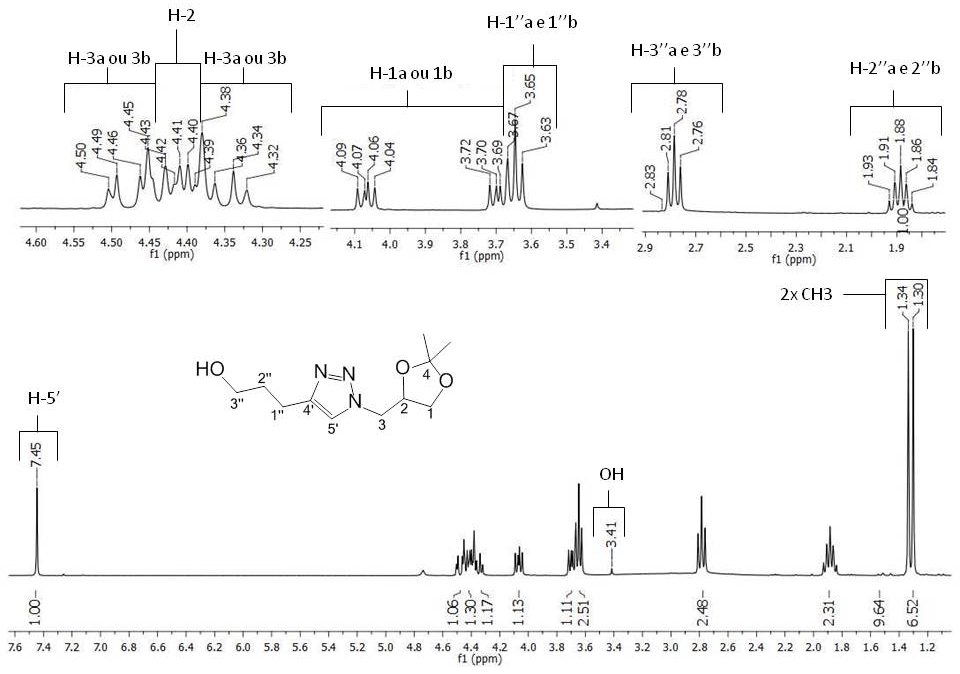


**Figure S22.** ^1^H NMR spectrum (300 MHz, CDCl_3_) of 3-(1-((2,2-dimethyl-1,3-dioxolan-4-yl)methyl)-1*H*-1,2,3-triazol-4-yl)propan-1-ol (**4c**).


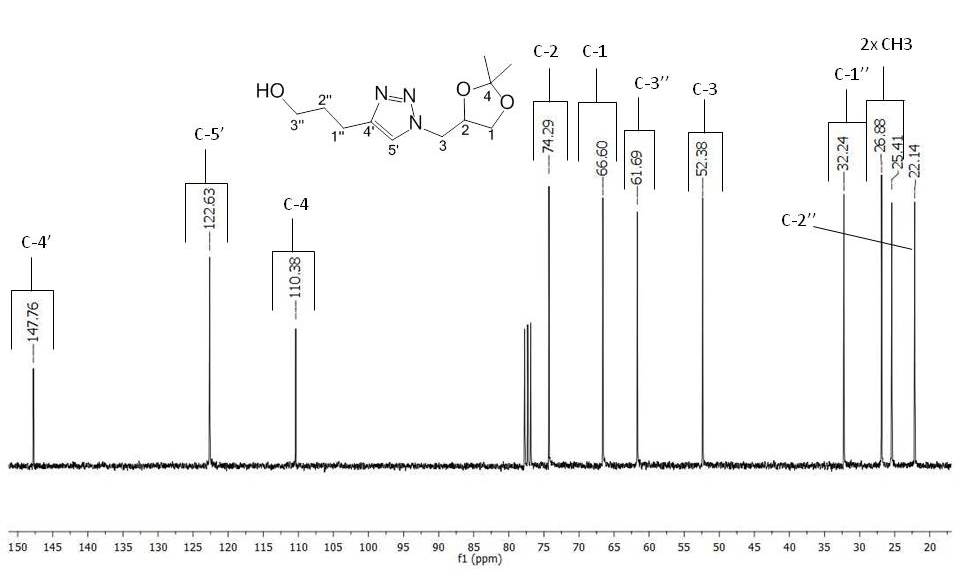


**Figure S23.** ^13^C NMR spectrum (75 MHz, CDCl_3_) of 3-(1-((2,2-dimethyl-1,3-dioxolan-4-yl)methyl)-1*H*-1,2,3-triazol-4-yl)propan-1-ol (**4c**).


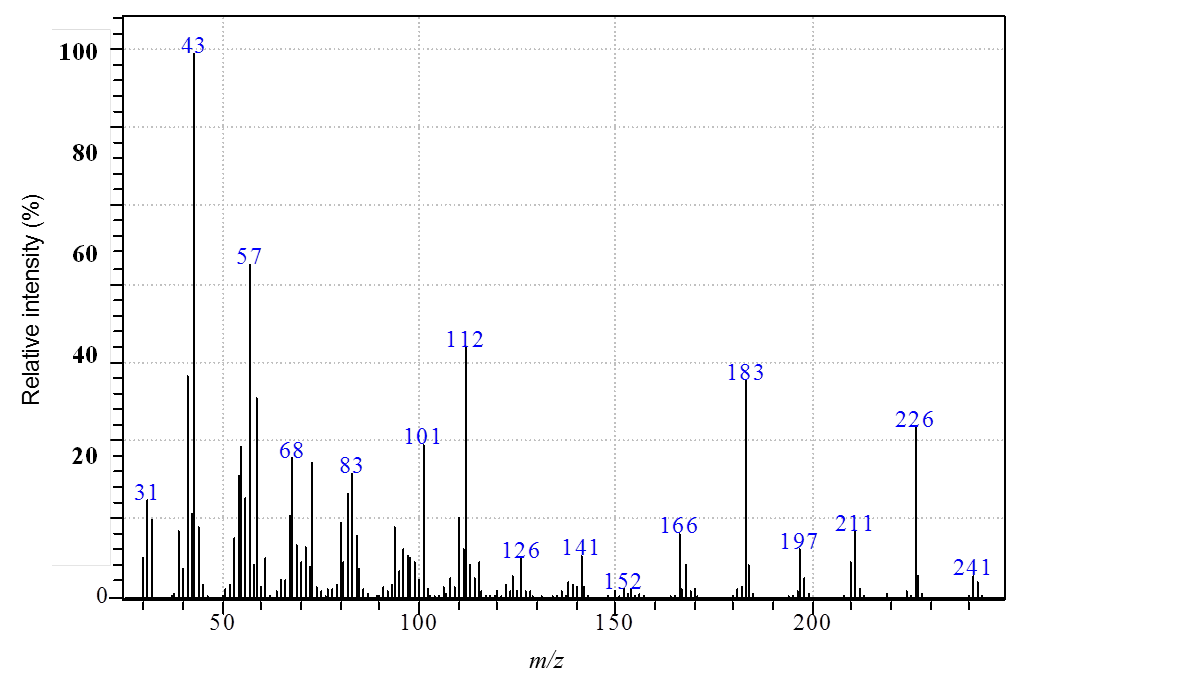


**Figure S24.** MS spectrum of 3-(1-((2,2-dimethyl-1,3-dioxolan-4-yl)methyl)-1*H*-1,2,3-triazol-4-yl)propan-1-ol (**4c**).

**
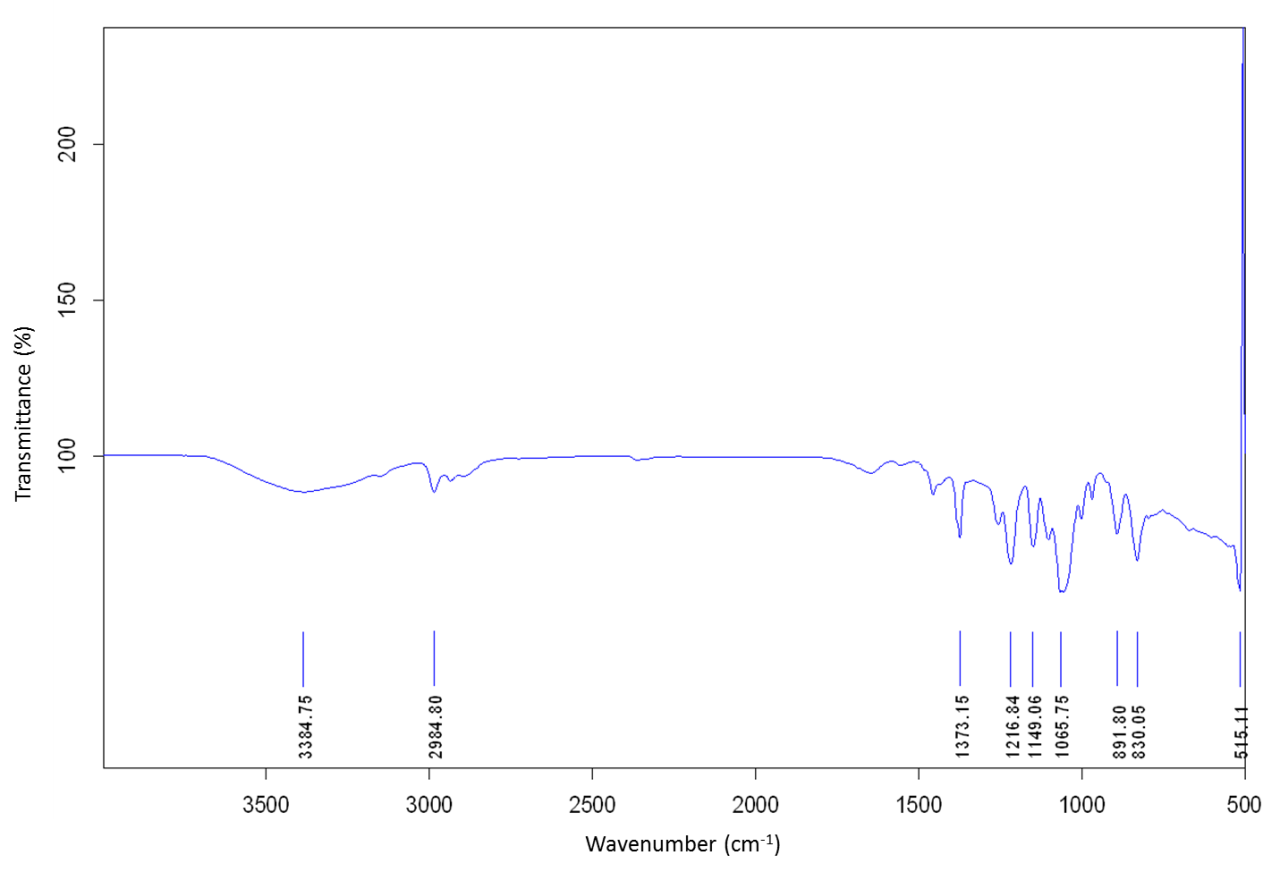
**

**Figure S25.** IR spectrum of 1-(1-((2,2-dimethyl-1,3-dioxolan-4-yl)methyl)-1*H*-1,2,3-triazol-4-yl)ethanol (**4d**).


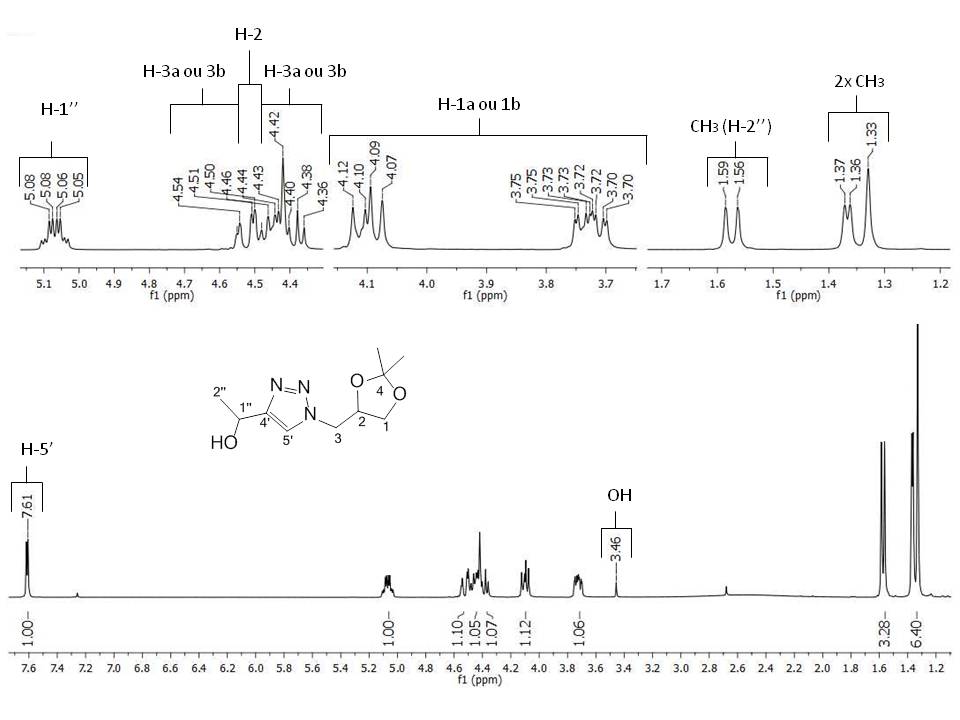


**Figure S26.** ^1^H NMR spectrum (300 MHz, CDCl_3_) of 1-(1-((2,2-dimethyl-1,3-dioxolan-4-yl)methyl)-1*H*-1,2,3-triazol-4-yl)ethanol (**4d**).


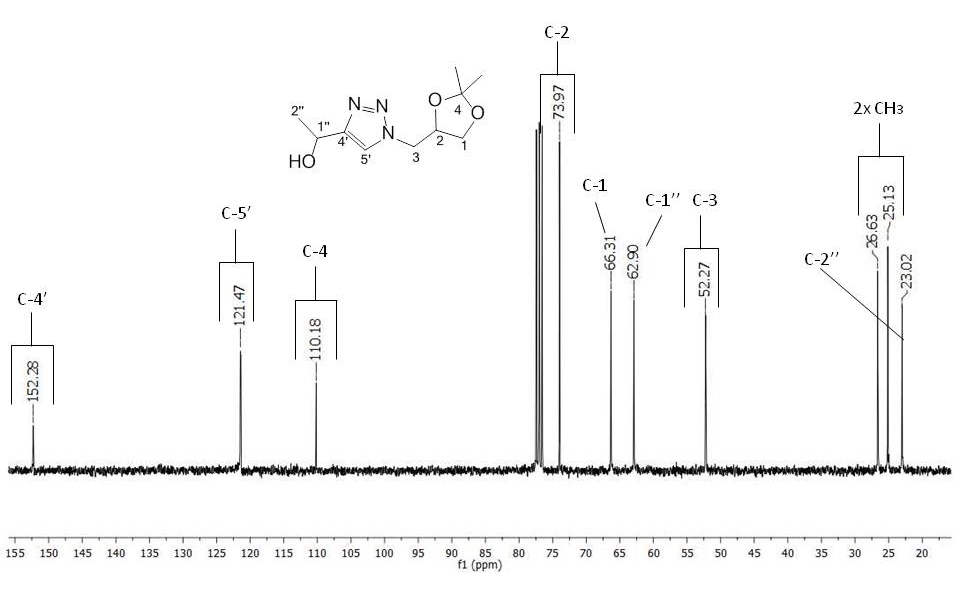


**Figure S27.** ^13^C NMR spectrum (75 MHz, CDCl_3_) of 1-(1-((2,2-dimethyl-1,3-dioxolan-4-yl)methyl)-1*H*-1,2,3-triazol-4-yl)ethanol (**4d**).


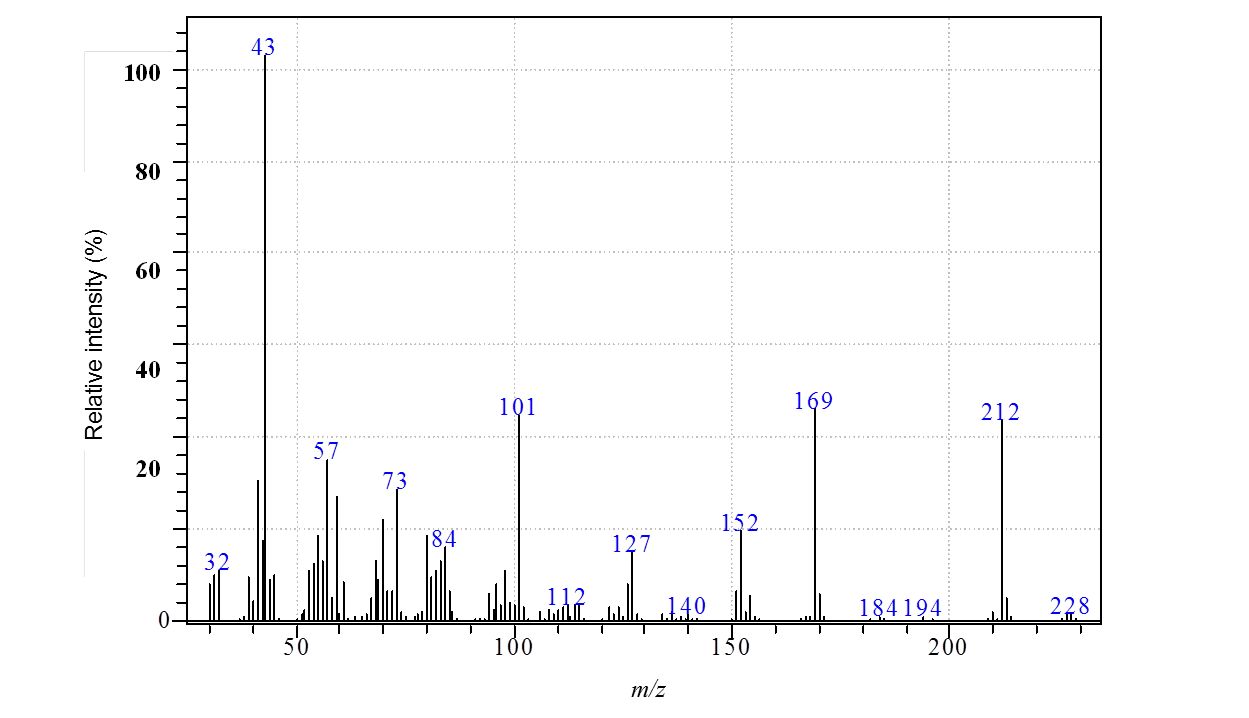


**Figure S28.** MS spectrum of 1-(1-((2,2-dimethyl-1,3-dioxolan-4-yl)methyl)-1*H*-1,2,3-triazol-4-yl)ethanol (**4d**).

**
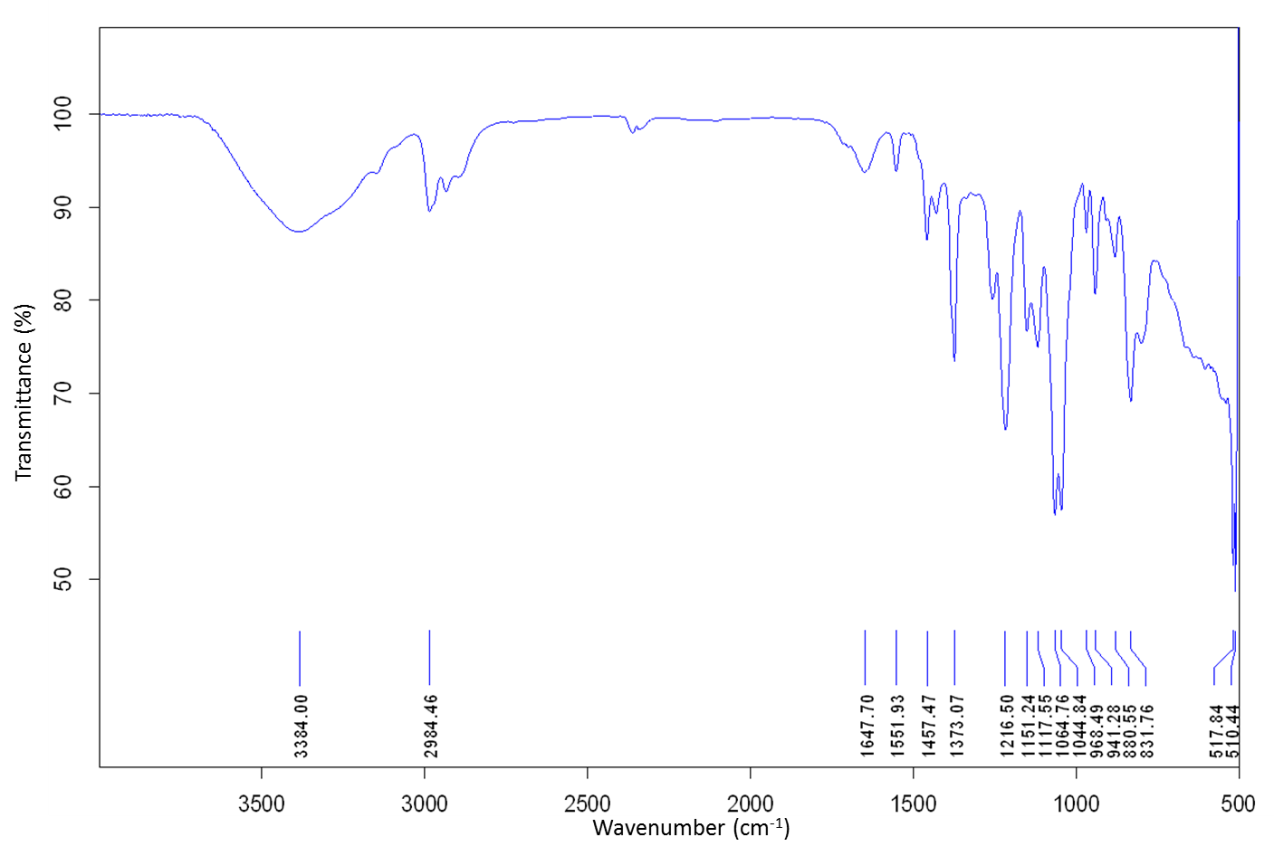
**

**Figure S29.** IR spectrum of 1-(1-((2,2-dimethyl-1,3-dioxolan-4-yl)methyl)-1*H*-1,2,3-triazol-4-yl)propan-2-ol (**4e**).


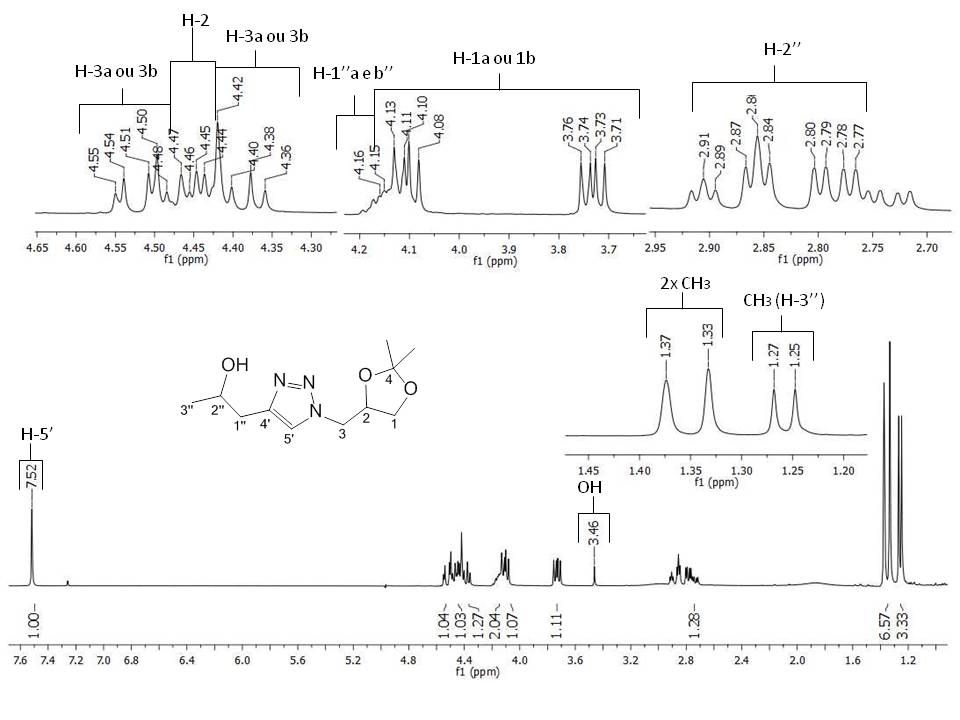


**Figure S30.** ^1^H NMR spectrum (300 MHz, CDCl_3_) of 1-(1-((2,2-dimethyl-1,3-dioxolan-4-yl)methyl)-1*H*-1,2,3-triazol-4-yl)propan-2-ol (**4e**).


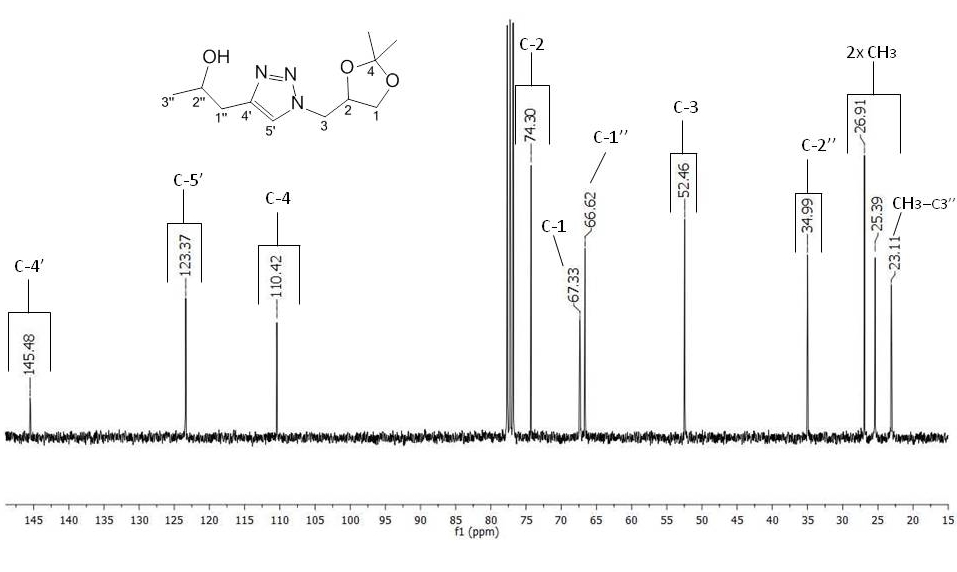


**Figure S31.** ^13^C NMR spectrum (75 MHz, CDCl_3_) of 1-(1-((2,2-dimethyl-1,3-dioxolan-4-yl)methyl)-1*H*-1,2,3-triazol-4-yl)propan-2-ol (**4e**).


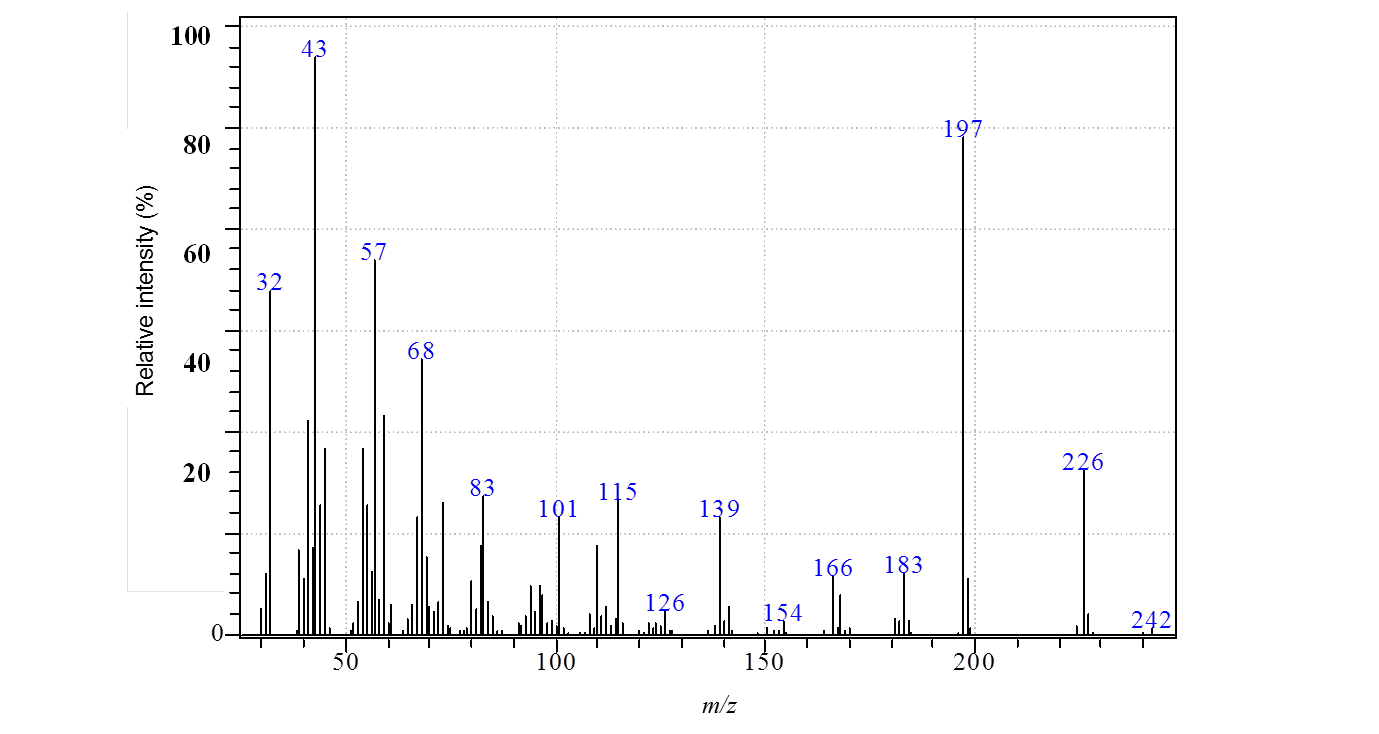


**Figure S32.** MS spectrum of 1-(1-((2,2-dimethyl-1,3-dioxolan-4-yl)methyl)-1*H*-1,2,3-triazol-4-yl)propan-2-ol (**4e**).


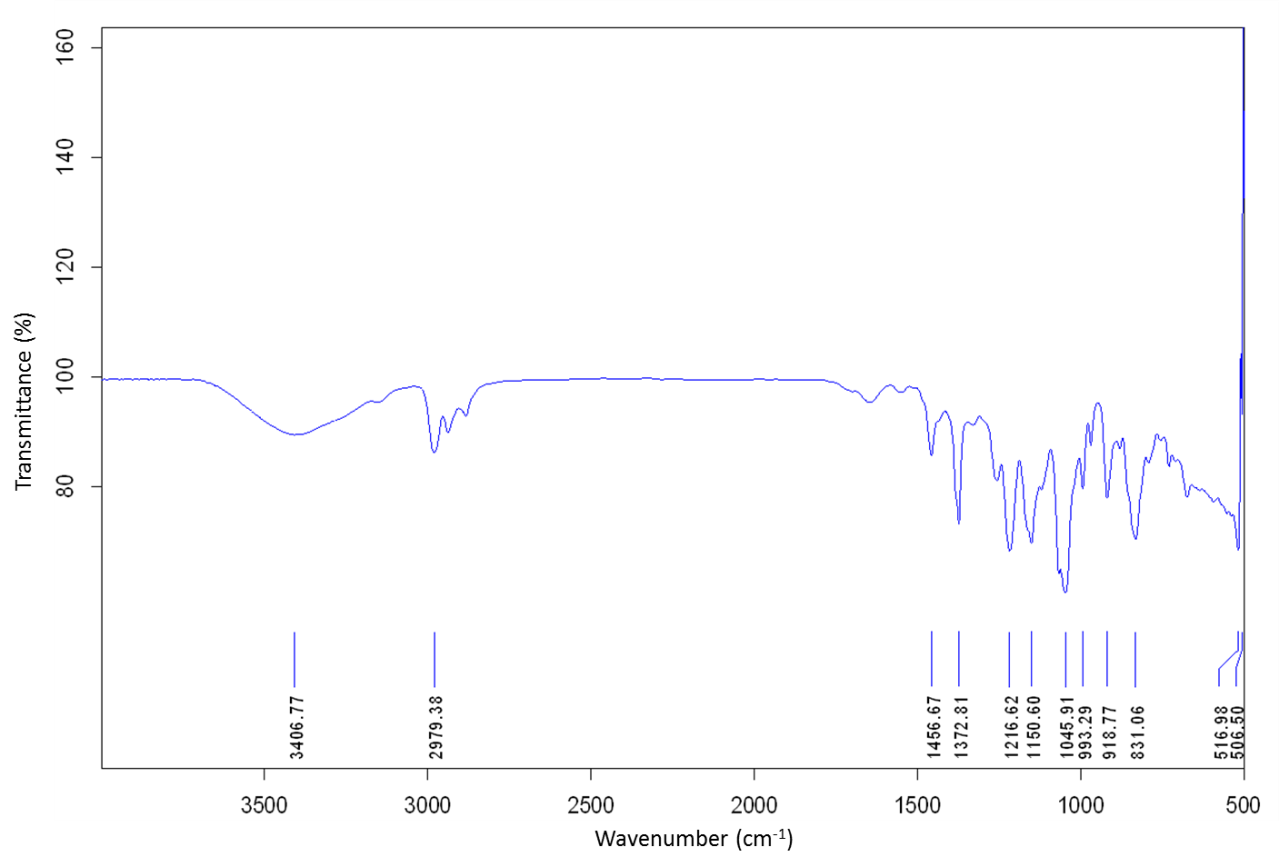


**Figure S33.** IR spectrum of 2-(1-((2,2-dimethyl-1,3-dioxolan-4-yl)methyl)-1*H*-1,2,3-triazol-4-yl)butan-2-ol (**4f**).


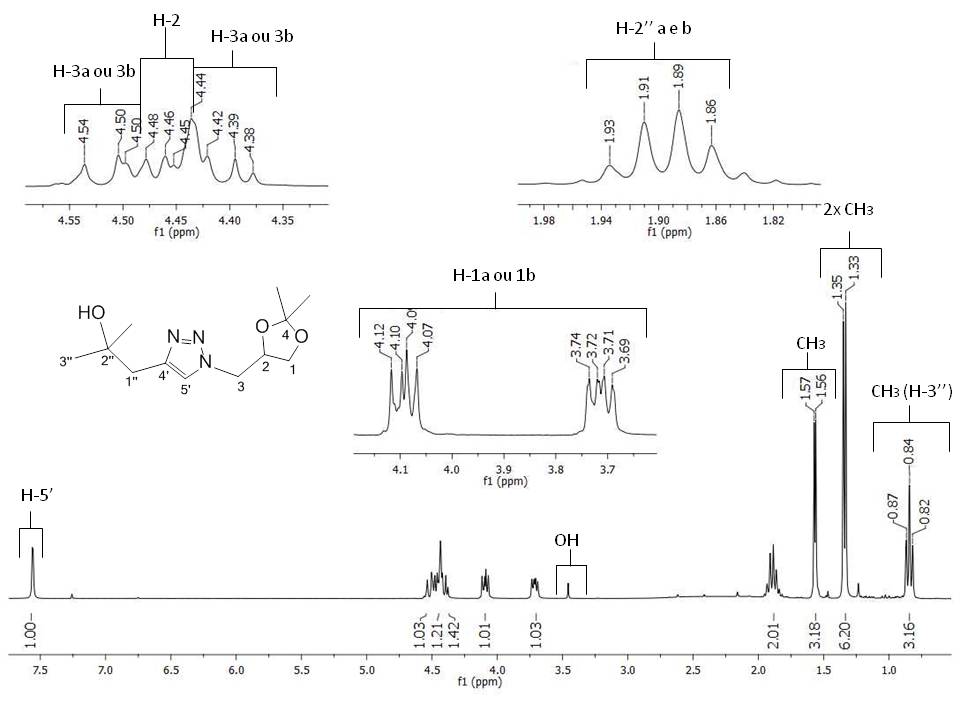


**Figure S34.** ^1^H NMR spectrum (300 MHz, CDCl_3_) of 2-(1-((2,2-dimethyl-1,3-dioxolan-4-yl)methyl)-1*H*-1,2,3-triazol-4-yl)butan-2-ol (**4f**).


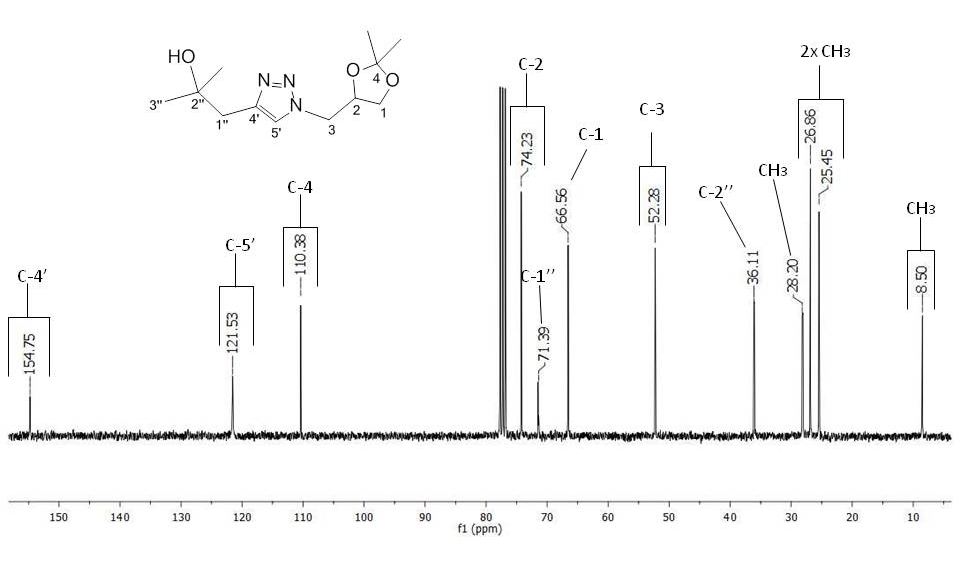


**Figure S35.** ^13^C NMR spectrum (75 MHz, CDCl_3_) of 2-(1-((2,2-dimethyl-1,3-dioxolan-4-yl)methyl)-1*H*-1,2,3-triazol-4-yl)butan-2-ol (**4f**).


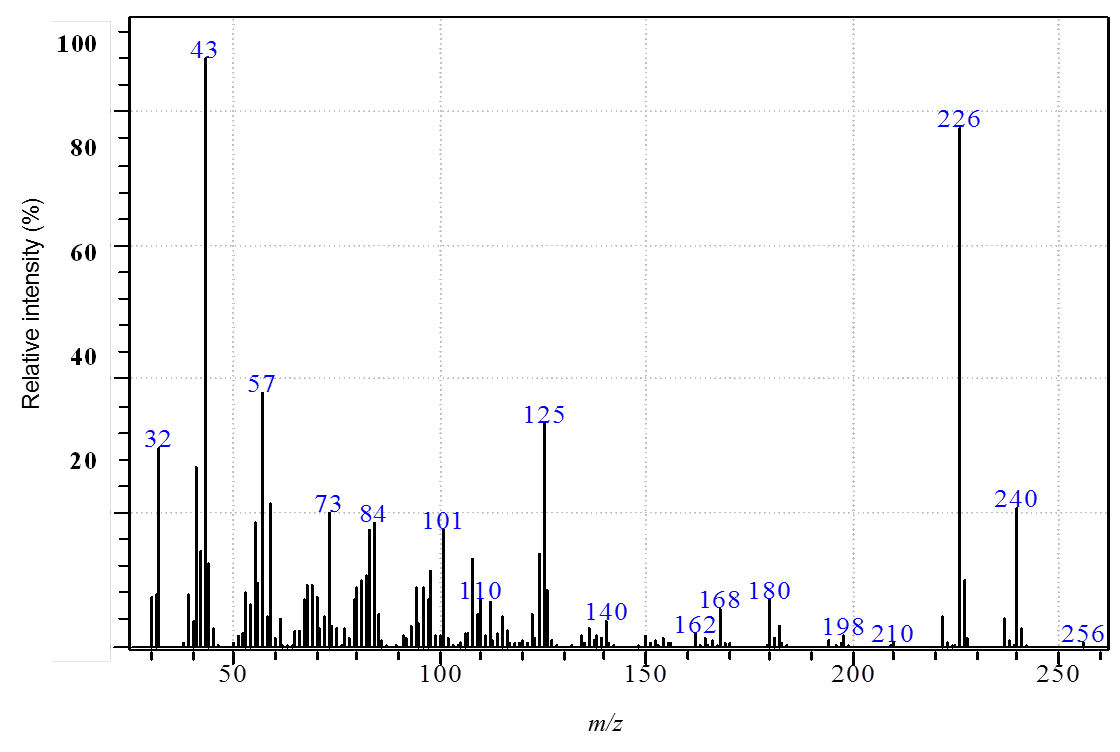


**Figure S36.** MS spectrum of 2-(1-((2,2-dimethyl-1,3-dioxolan-4-yl)methyl)-1*H*-1,2,3-triazol-4-yl)butan-2-ol (**4f**).

**
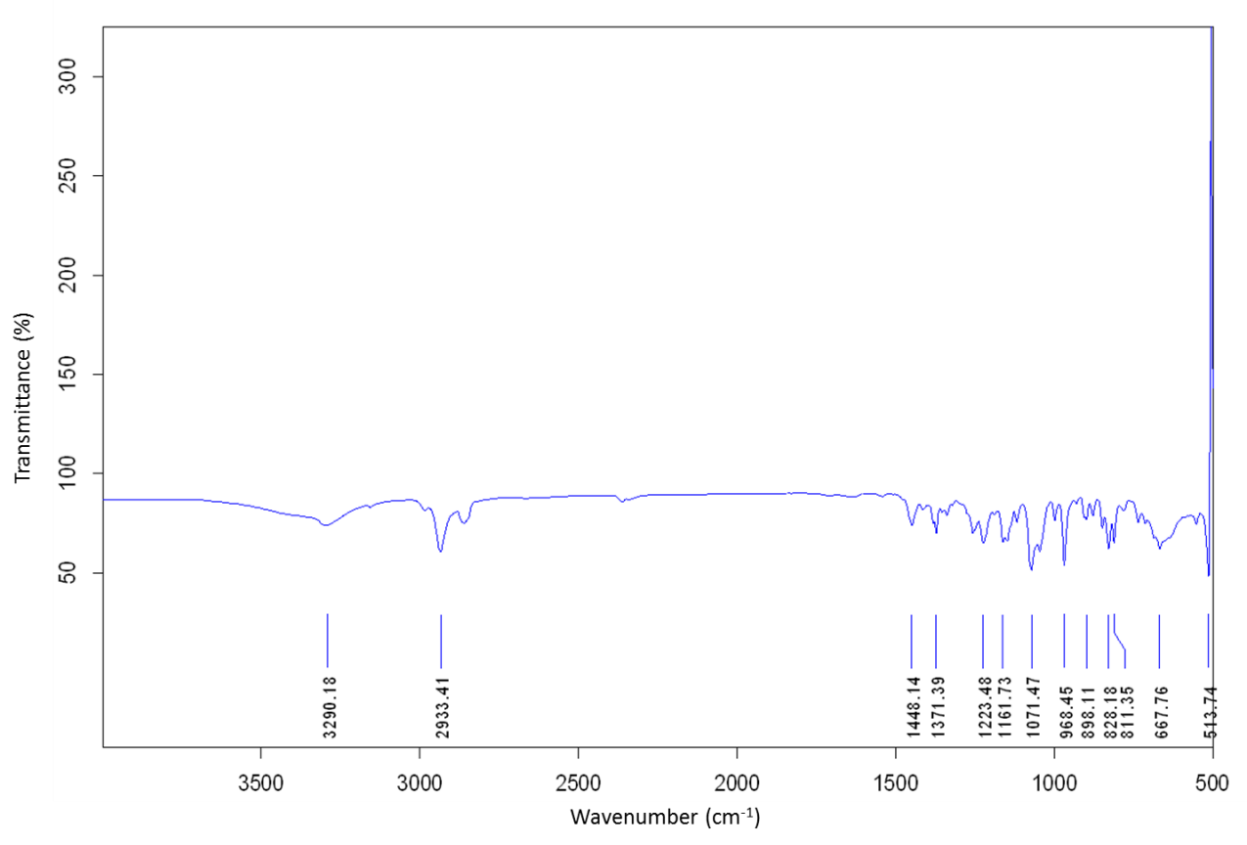
**

**Figure S37.** IR spectrum of 1-(1-((2,2-dimethyl-1,3-dioxolan-4-yl)methyl)-1*H*-1,2,3-triazol-4-yl)cyclohexanol (**4g**).


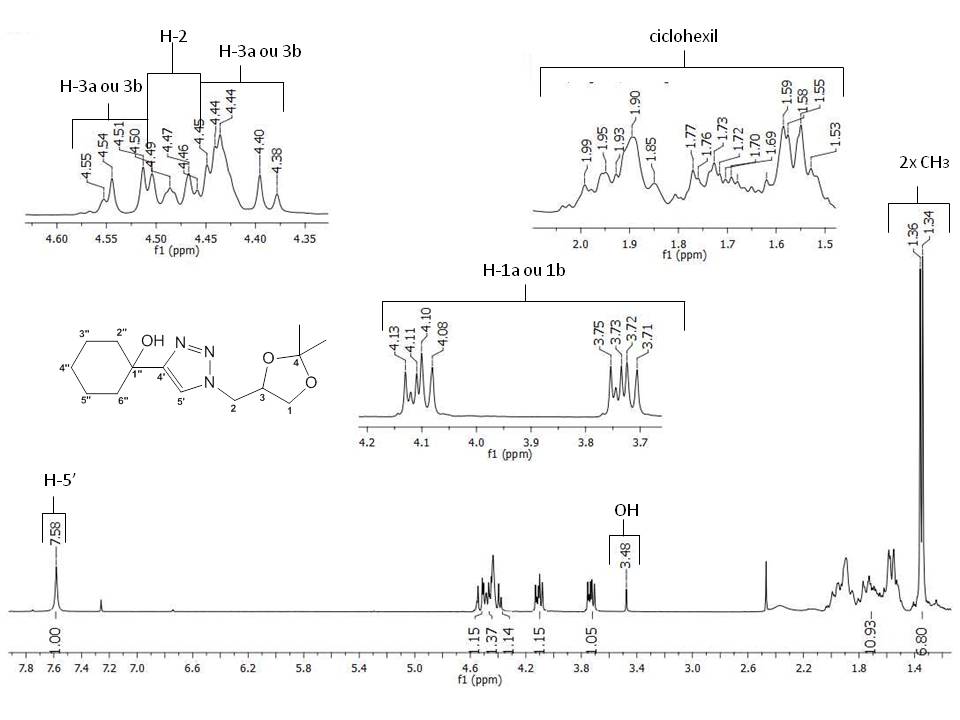


**Figure S38.** ^1^H NMR spectrum (300 MHz, CDCl_3_) of 1-(1-((2,2-dimethyl-1,3-dioxolan-4-yl)methyl)-1*H*-1,2,3-triazol-4-yl)cyclohexanol (**4g**).


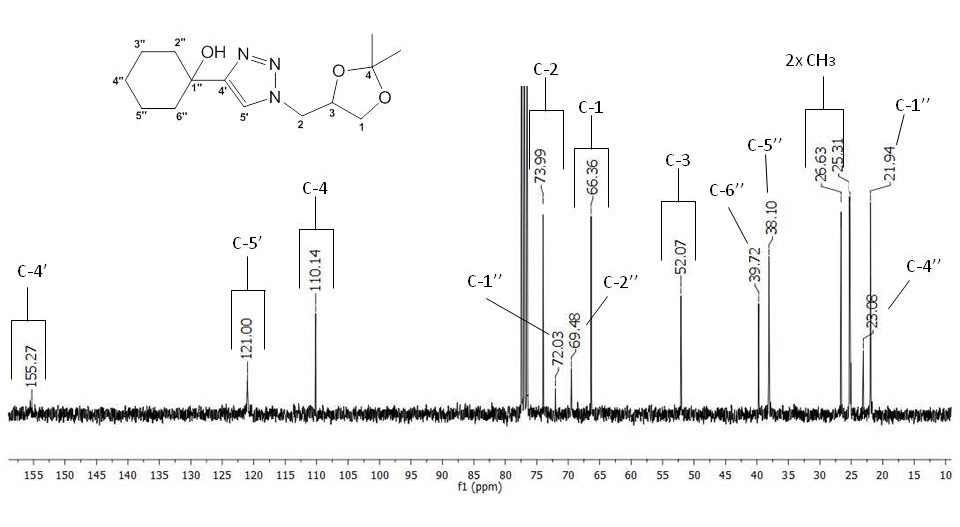


**Figure S39.** ^13^C NMR spectrum (75 MHz, CDCl_3_) of 1-(1-((2,2-dimethyl-1,3-dioxolan-4-yl)methyl)-1*H*-1,2,3-triazol-4-yl)cyclohexanol (**4g**).


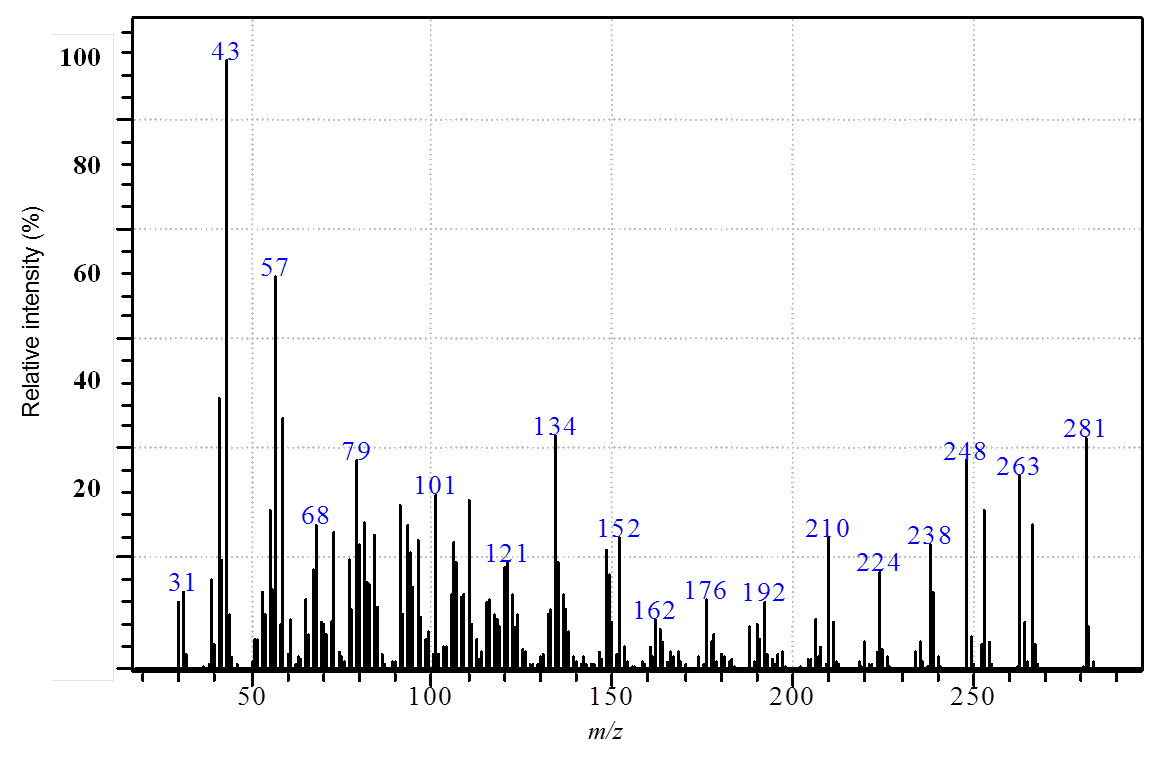


**Figure S40.** MS spectrum of 1-(1-((2,2-dimethyl-1,3-dioxolan-4-yl)methyl)-1*H*-1,2,3-triazol-4-yl)cyclohexanol (**4g**).

**
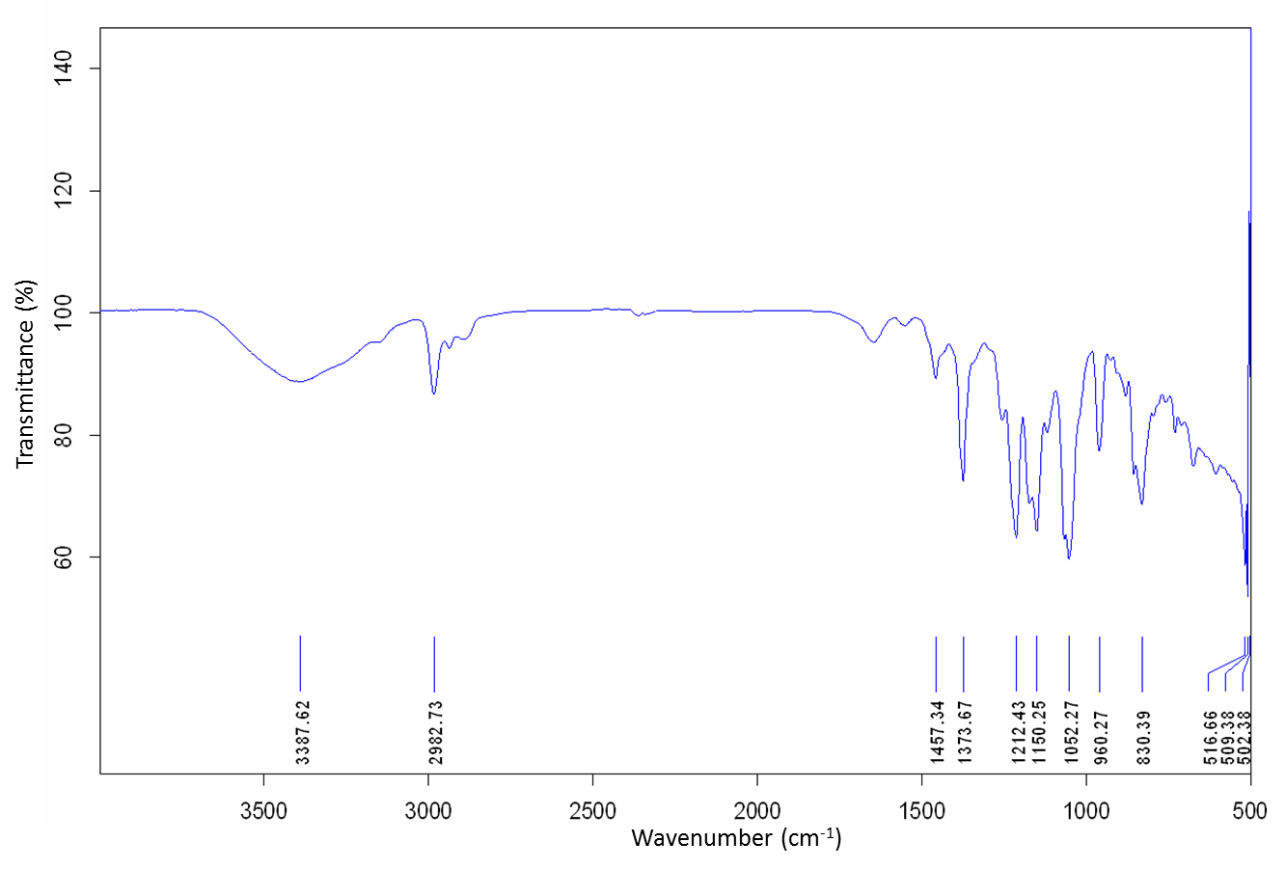
**

**Figure S41.** IR spectrum of 2-(1-((2,2-dimethyl-1,3-dioxolan-4-yl)methyl)-1*H*-1,2,3-triazol-4-yl)propan-2-ol (**4h**).


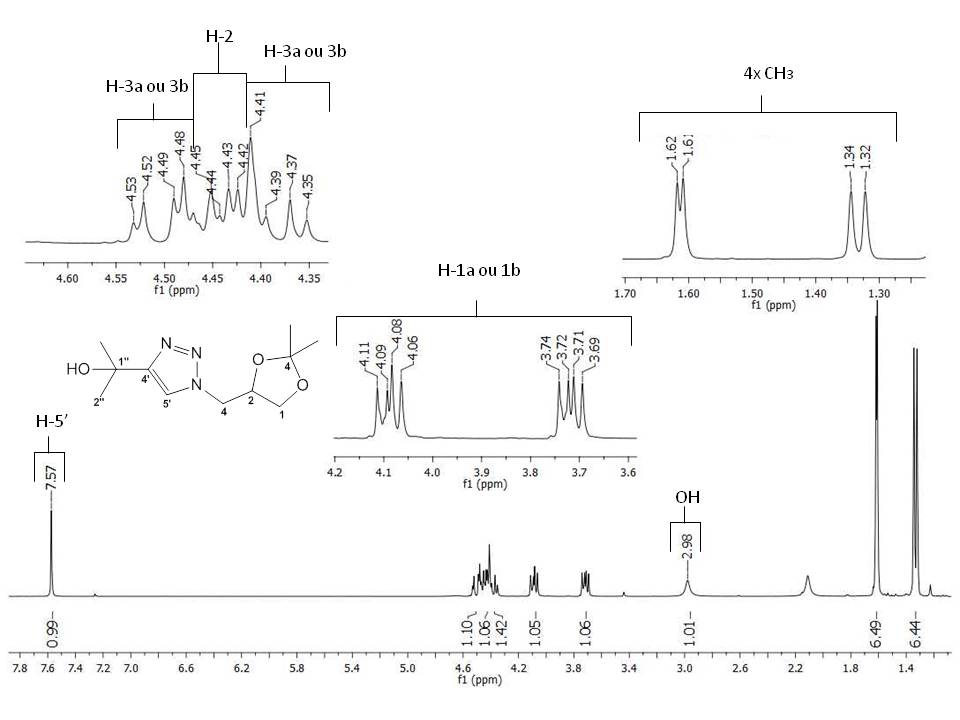


**Figure S42.** ^1^H NMR spectrum (300 MHz, CDCl_3_) of 2-(1-((2,2-dimethyl-1,3-dioxolan-4-yl)methyl)-1*H*-1,2,3-triazol-4-yl)propan-2-ol (**4h**).


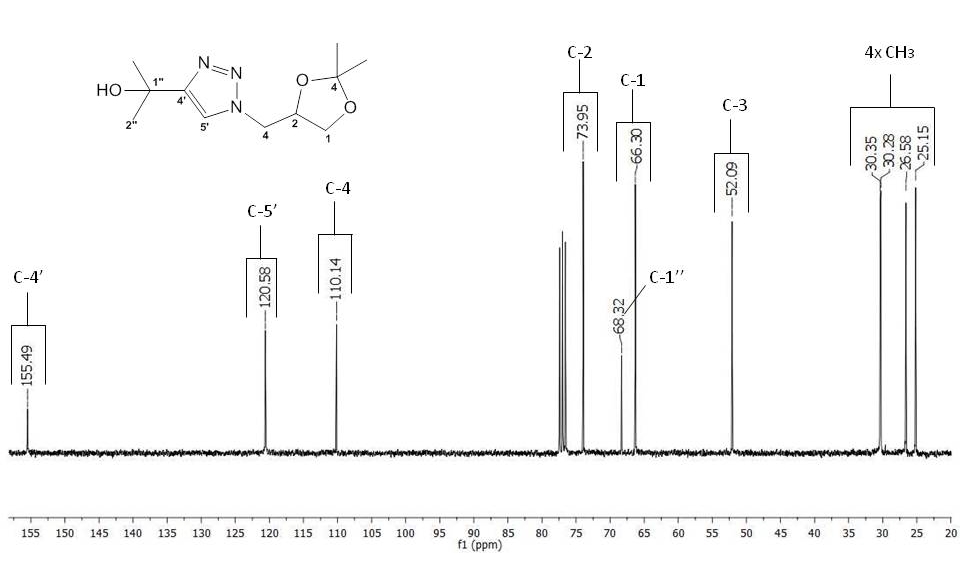


**Figure S43.** ^13^C NMR spectrum (75 MHz, CDCl_3_) of 2-(1-((2,2-dimethyl-1,3-dioxolan-4-yl)methyl)-1*H*-1,2,3-triazol-4-yl)propan-2-ol (**4h**).


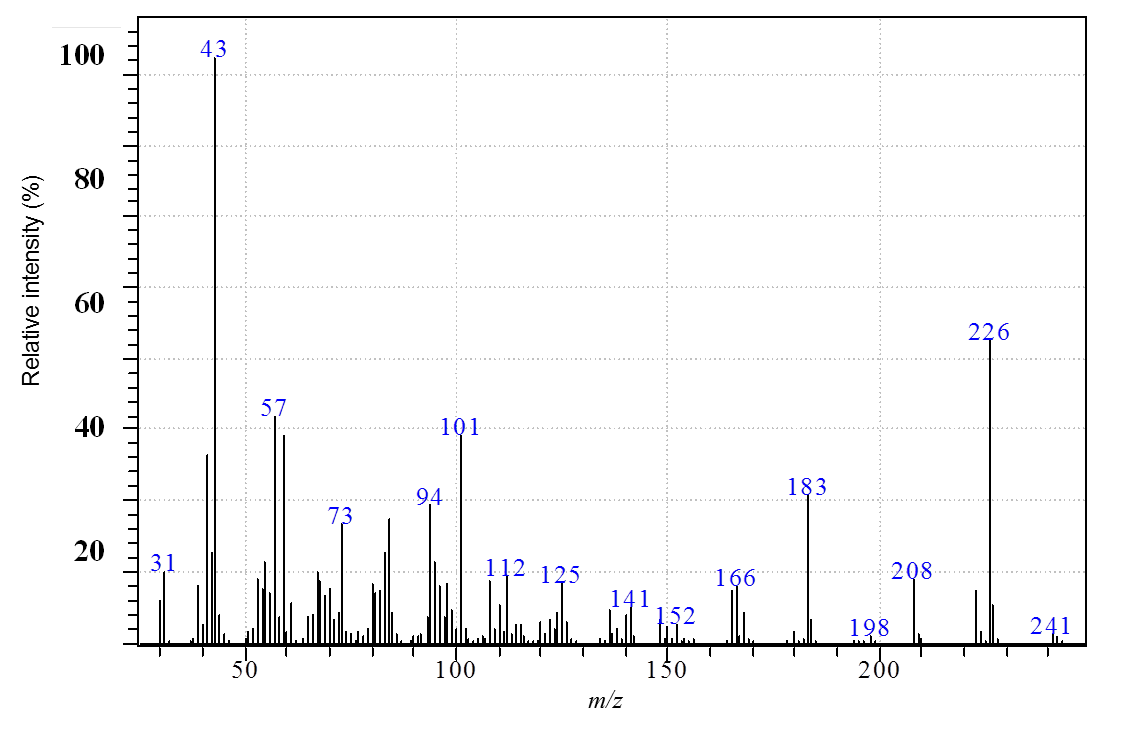


**Figure S44.** MS spectrum of 2-(1-((2,2-dimethyl-1,3-dioxolan-4-yl)methyl)-1*H*-1,2,3-triazol-4-yl)propan-2-ol (**4h**).

**
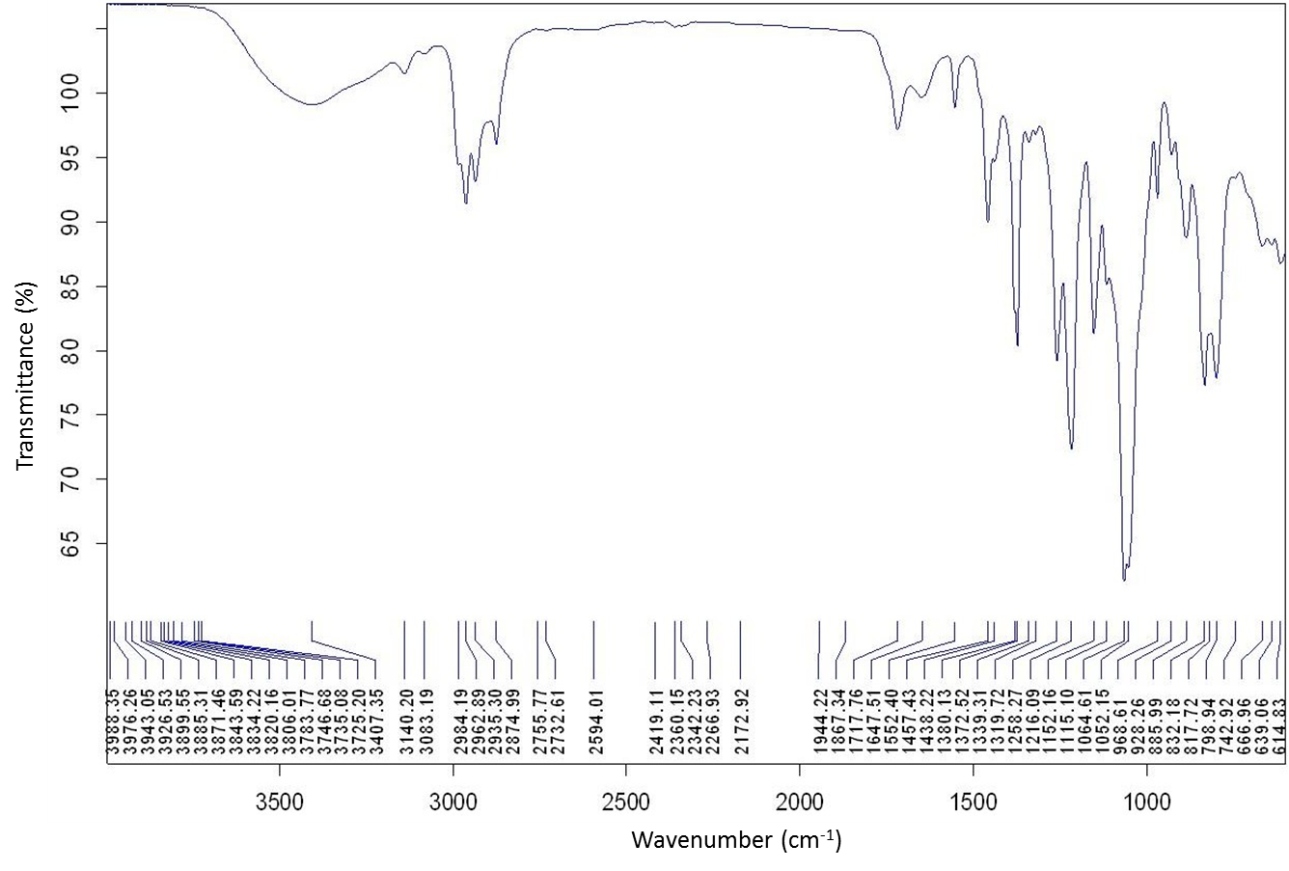
**

**Figure S45.** IR spectrum of 1-((2,2-dimethyl-1,3-dioxolan-4-yl)methyl)-4-propyl-1*H*-1,2,3-triazole (**4i**).


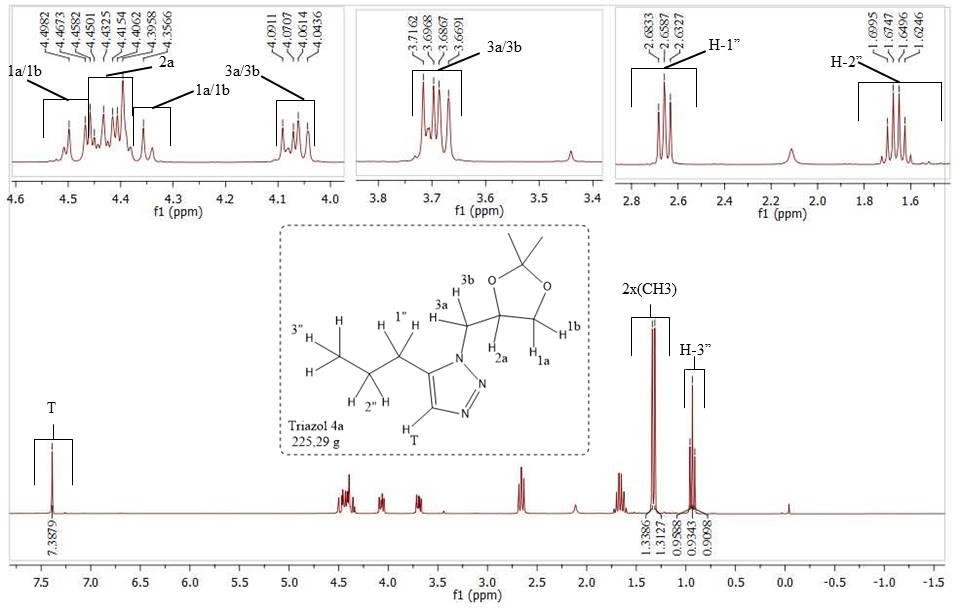


**Figure S46.** ^1^H NMR spectrum (300 MHz, CDCl_3_) of 1-((2,2-dimethyl-1,3-dioxolan-4-yl)methyl)-4-propyl-1*H*-1,2,3-triazole (**4i**).


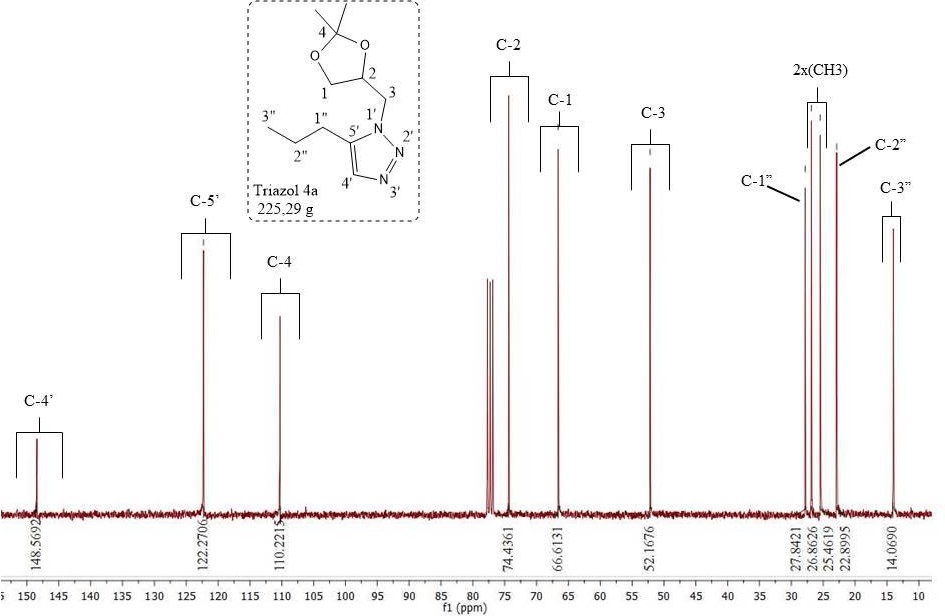


**Figure S47.** ^13^C NMR spectrum (75 MHz, CDCl_3_) of 1-((2,2-dimethyl-1,3-dioxolan-4-yl)methyl)-4-propyl-1*H*-1,2,3- triazole (**4i**).


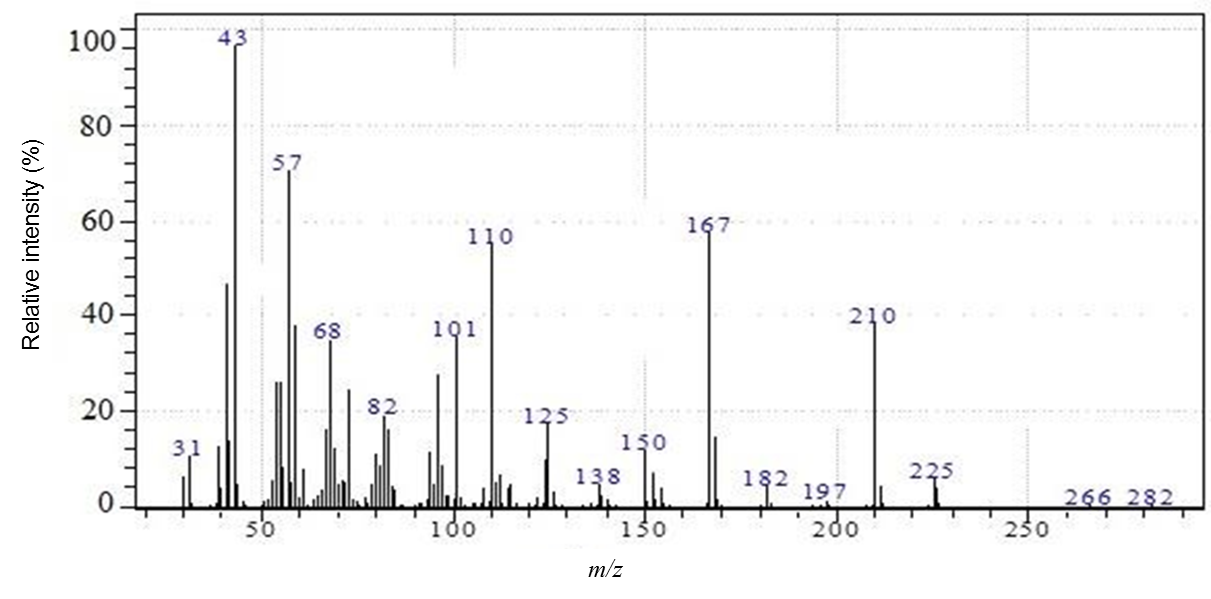


**Figure S48.** MS spectrum of 1-((2,2-dimethyl-1,3-dioxolan-4-yl)methyl)-4-propyl-1*H*-1,2,3- triazole (**4i**).

**
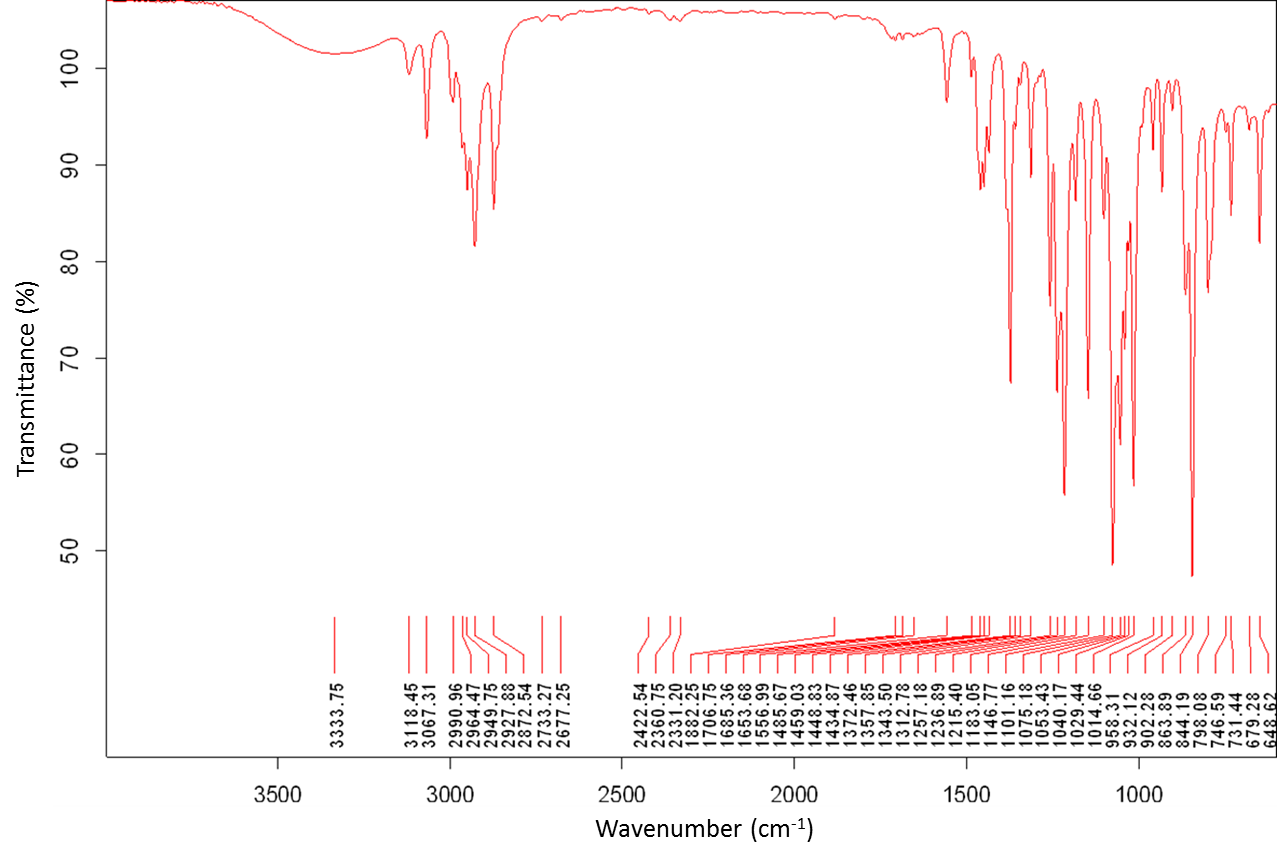
**

**Figure S49.** IR spectrum of 1-((2,2-dimethyl-1,3-dioxolan-4-yl)methyl)-4-butyl-1*H*-1,2,3-triazole (**4j**).


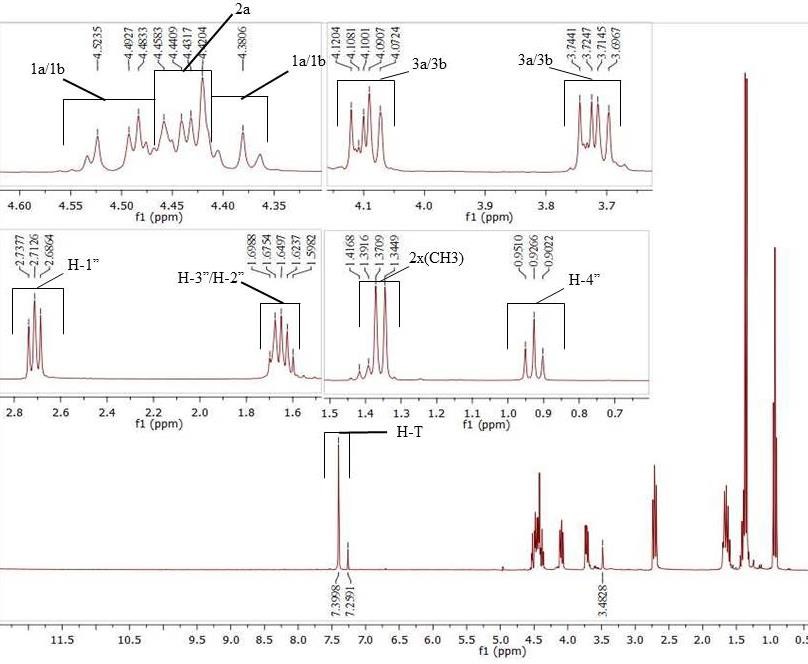

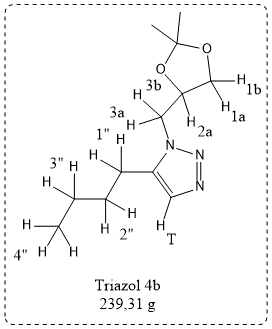


**Figure S50.** ^1^H NMR spectrum (300 MHz, CDCl_3_) of 1-((2,2-dimethyl-1,3-dioxolan-4-yl)methyl)-4-butyl-1*H*-1,2,3-triazole (**4j**).


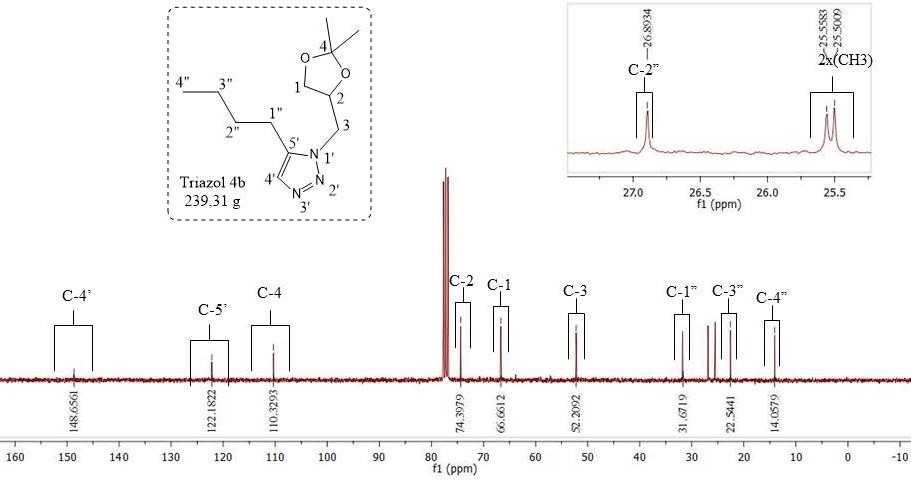


**Figure S51.** ^13^C NMR spectrum (75 MHz, CDCl_3_) of 1-((2,2-dimethyl-1,3-dioxolan-4-yl)methyl)-4-butyl-1*H*-1,2,3-triazole (**4j**).


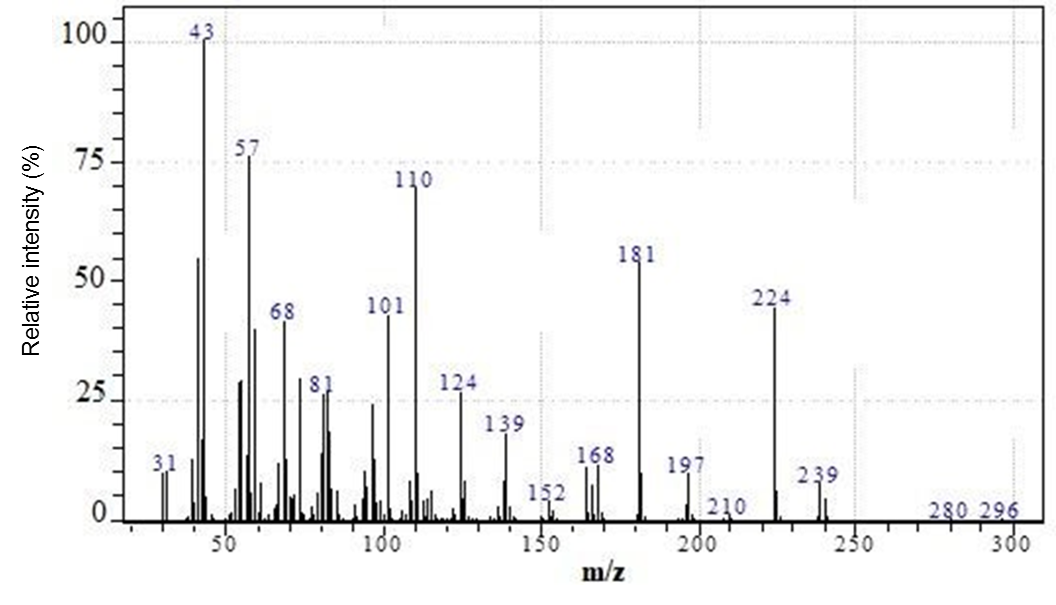


**Figure S52.** MS spectrum of 1-((2,2-dimethyl-1,3-dioxolan-4-yl)methyl)-4-butyl-1*H*-1,2,3-triazole (**4j**).

**
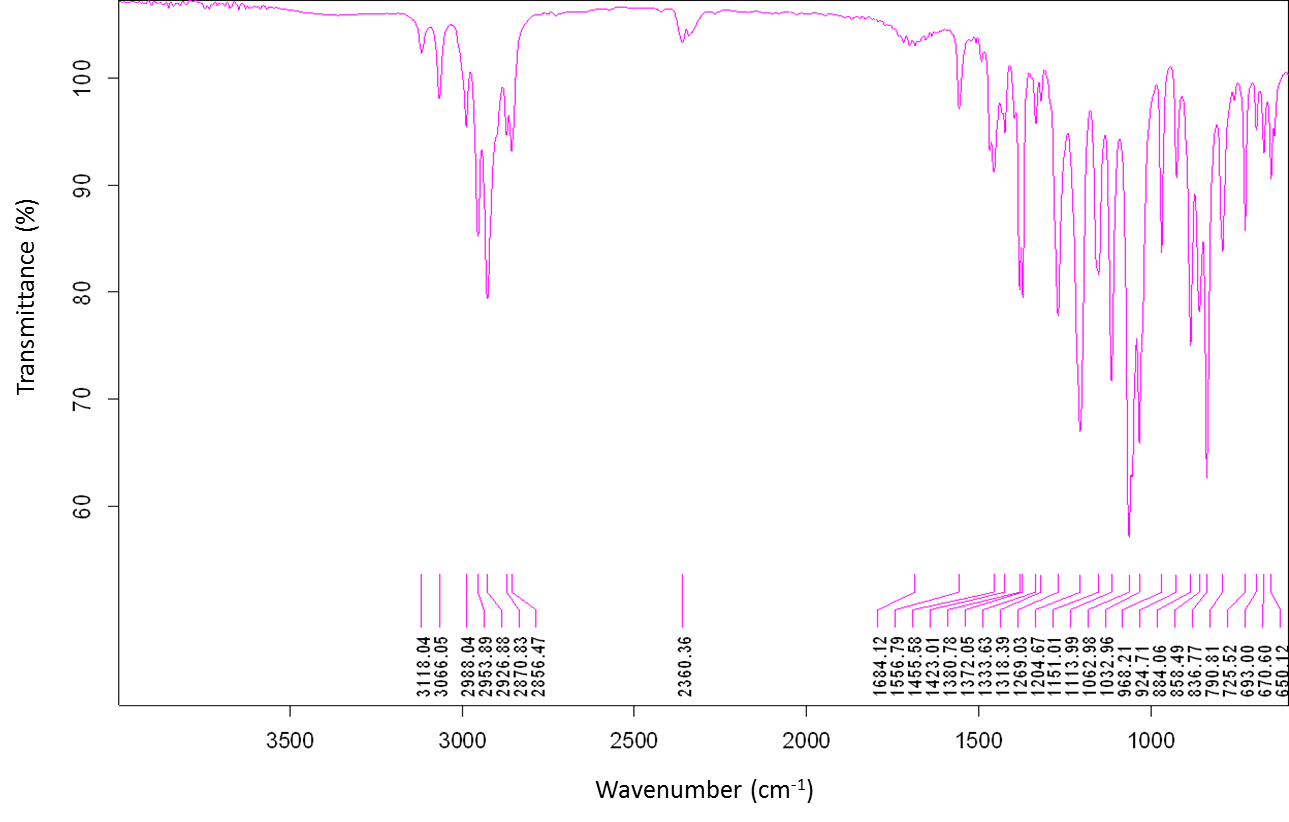
**

**Figure S53.** IR spectrum of 1-((2,2-dimethyl-1,3-dioxolan-4-yl)methyl)-4-pentyl-1*H*-1,2,3-triazole (**4k**).


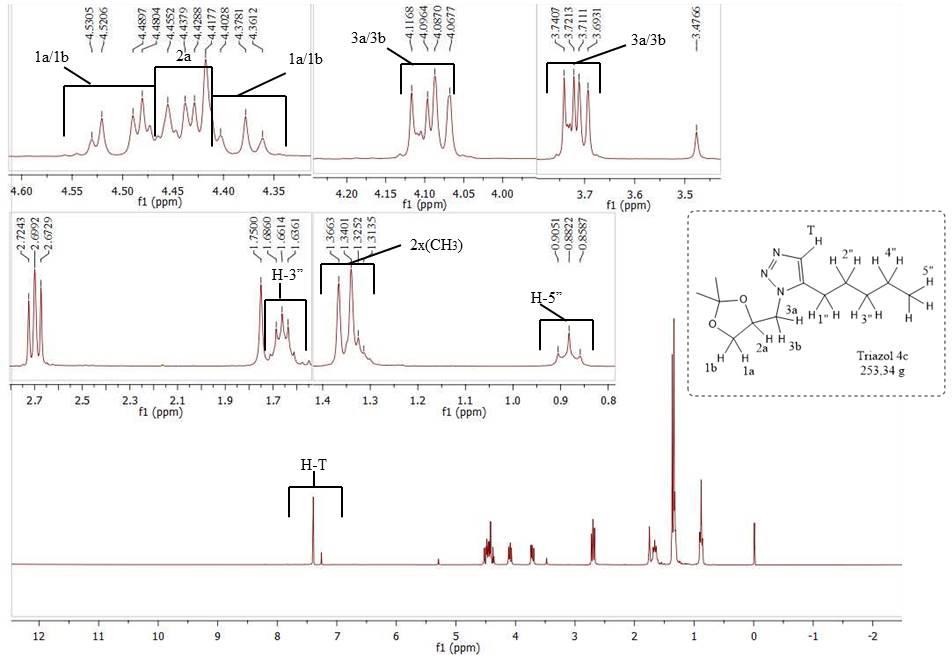


**Figure S54.** ^1^H NMR spectrum (300 MHz, CDCl_3_) of 1-((2,2-dimethyl-1,3-dioxolan-4-yl)methyl)-4-pentyl-1*H*-1,2,3-triazole (**4k**).


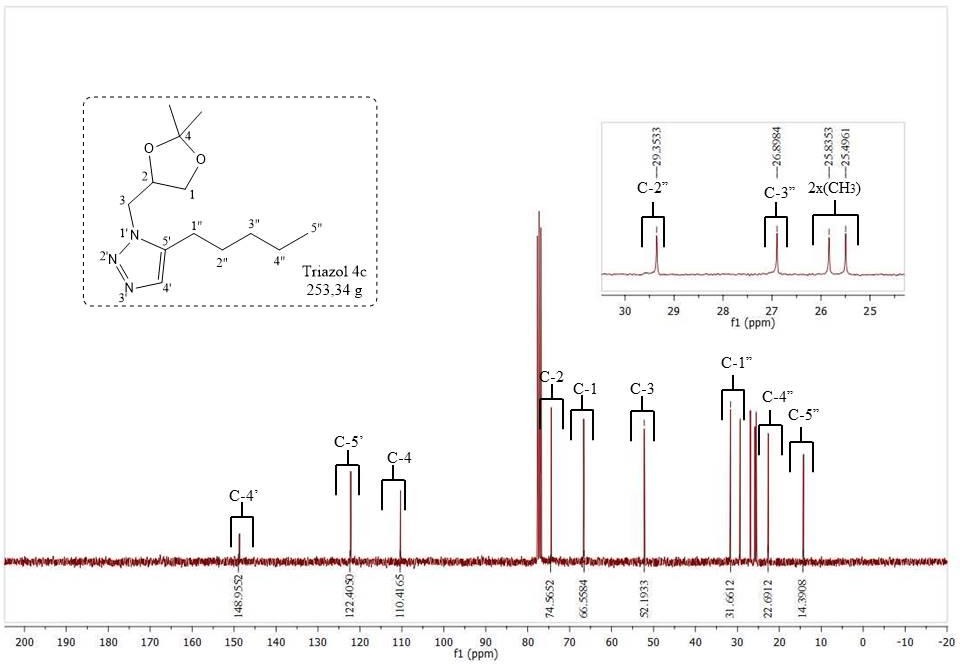


**Figure S55.** ^13^C NMR spectrum (75 MHz, CDCl_3_) of 1-((2,2-dimethyl-1,3-dioxolan-4-yl)methyl)-4-pentyl-1*H*-1,2,3-triazole (**4k**).


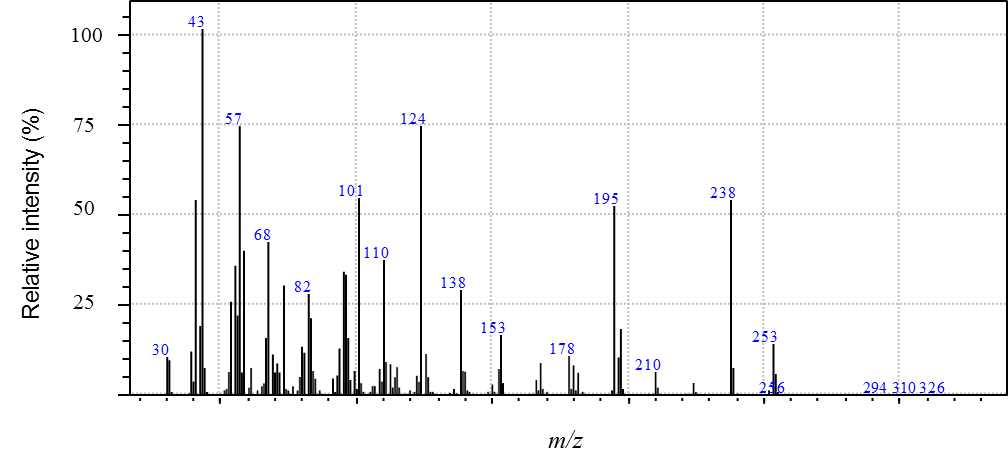


**Figure S56.** MS spectrum of 1-((2,2-dimethyl-1,3-dioxolan-4-yl)methyl)-4-pentyl-1*H*-1,2,3-triazole (**4k**).


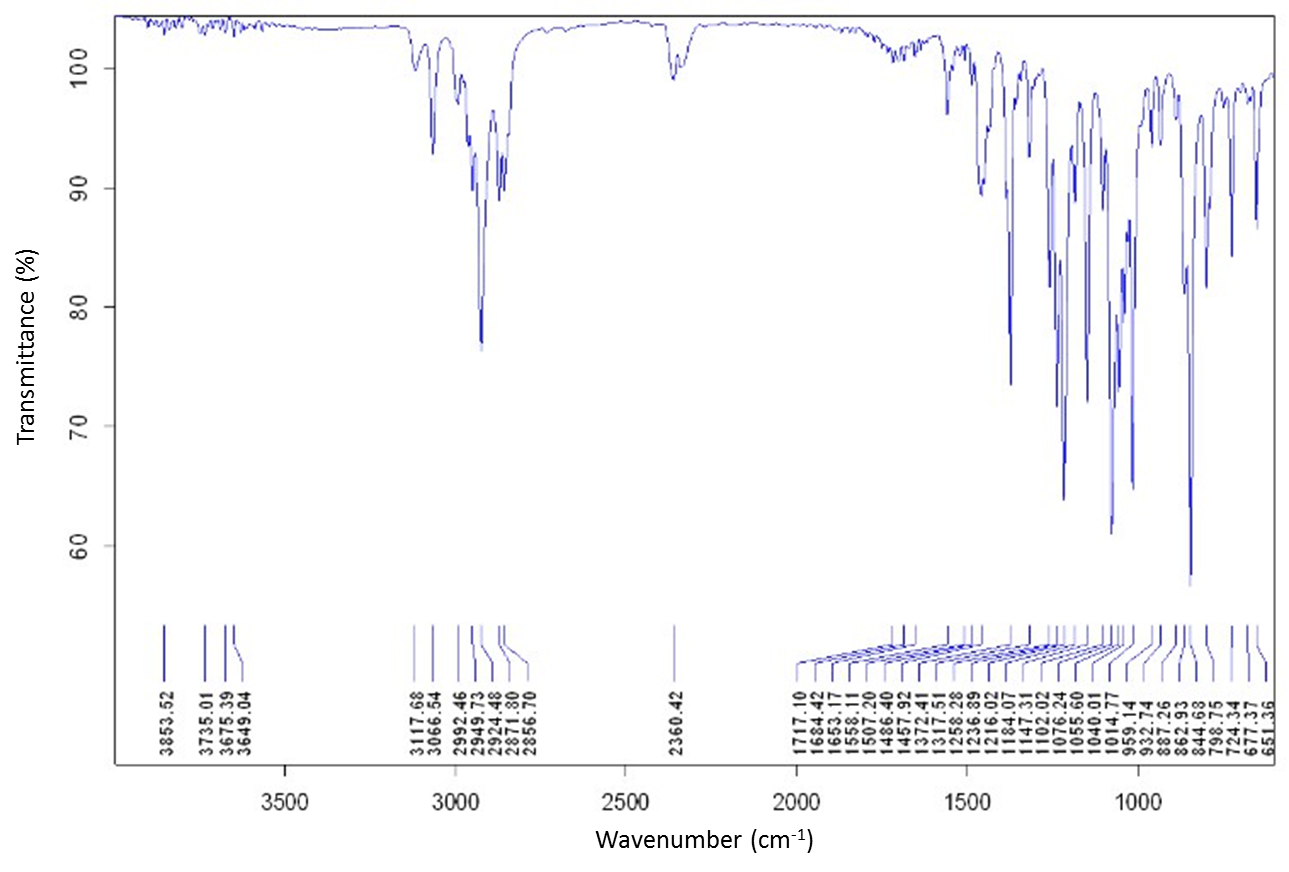


**Figure S57.** IR spectrum of 1-((2,2-dimethyl-1,3-dioxolan-4-yl)methyl)-4-hexyl-1*H*-1,2,3-triazole (**4l**).


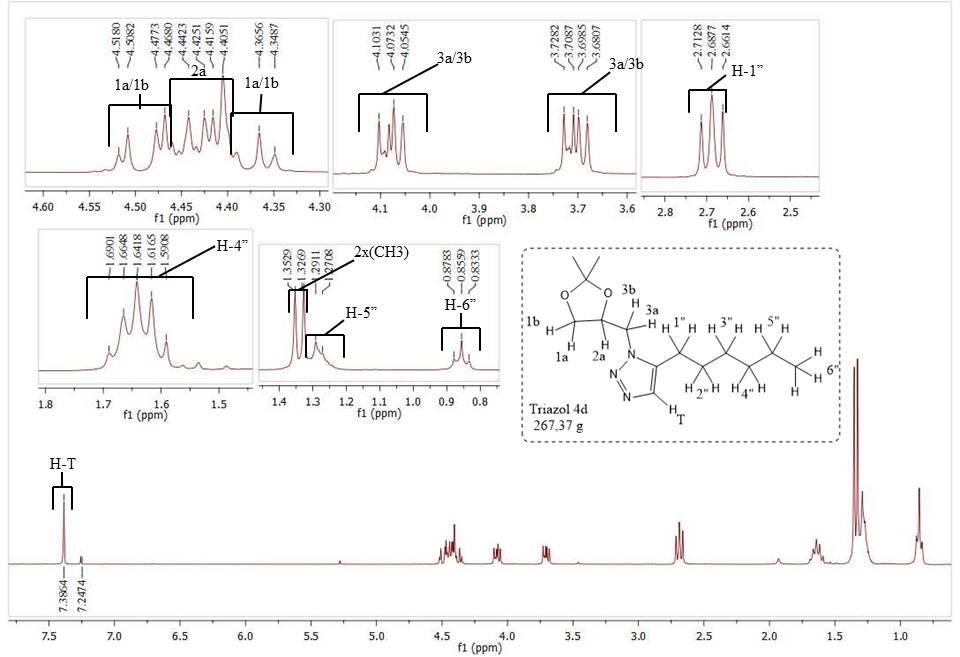


**Figure S58.** ^1^H NMR spectrum (300 MHz, CDCl_3_) of 1-((2,2-dimethyl-1,3-dioxolan-4-yl)methyl)-4-hexyl-1*H*-1,2,3-triazole (**4l**).


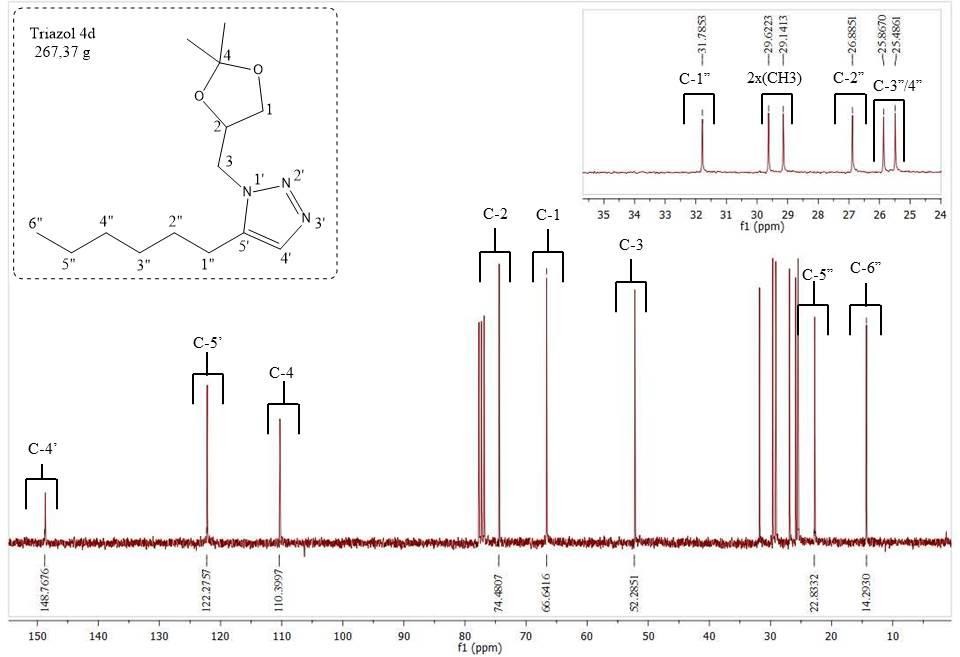


**Figure S59.** ^13^C NMR spectrum (75 MHz, CDCl_3_) of 1-((2,2-dimethyl-1,3-dioxolan-4-yl)methyl)-4-hexyl-1*H*-1,2,3-triazole (**4l**).


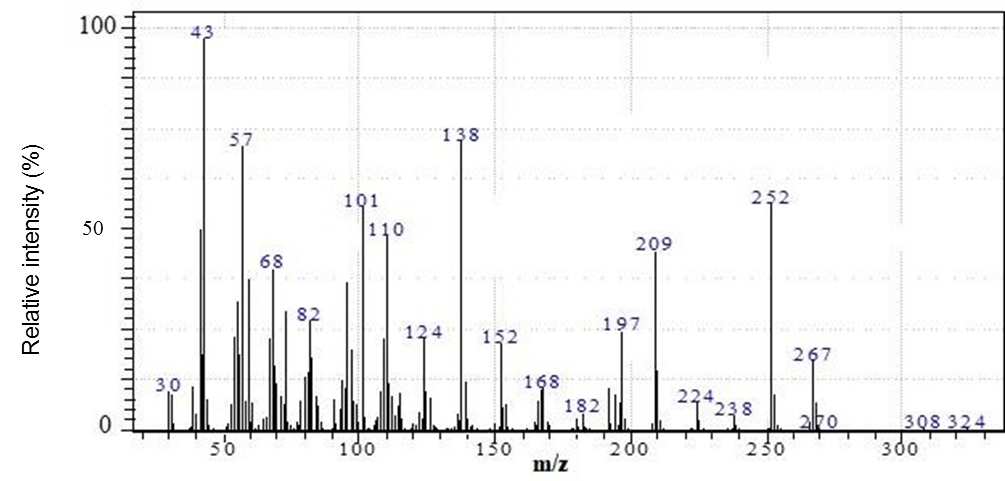


**Figure S60.** MS spectrum of 1-((2,2-dimethyl-1,3-dioxolan-4-yl)methyl)-4-hexyl-1*H*-1,2,3-triazole (**4l**).


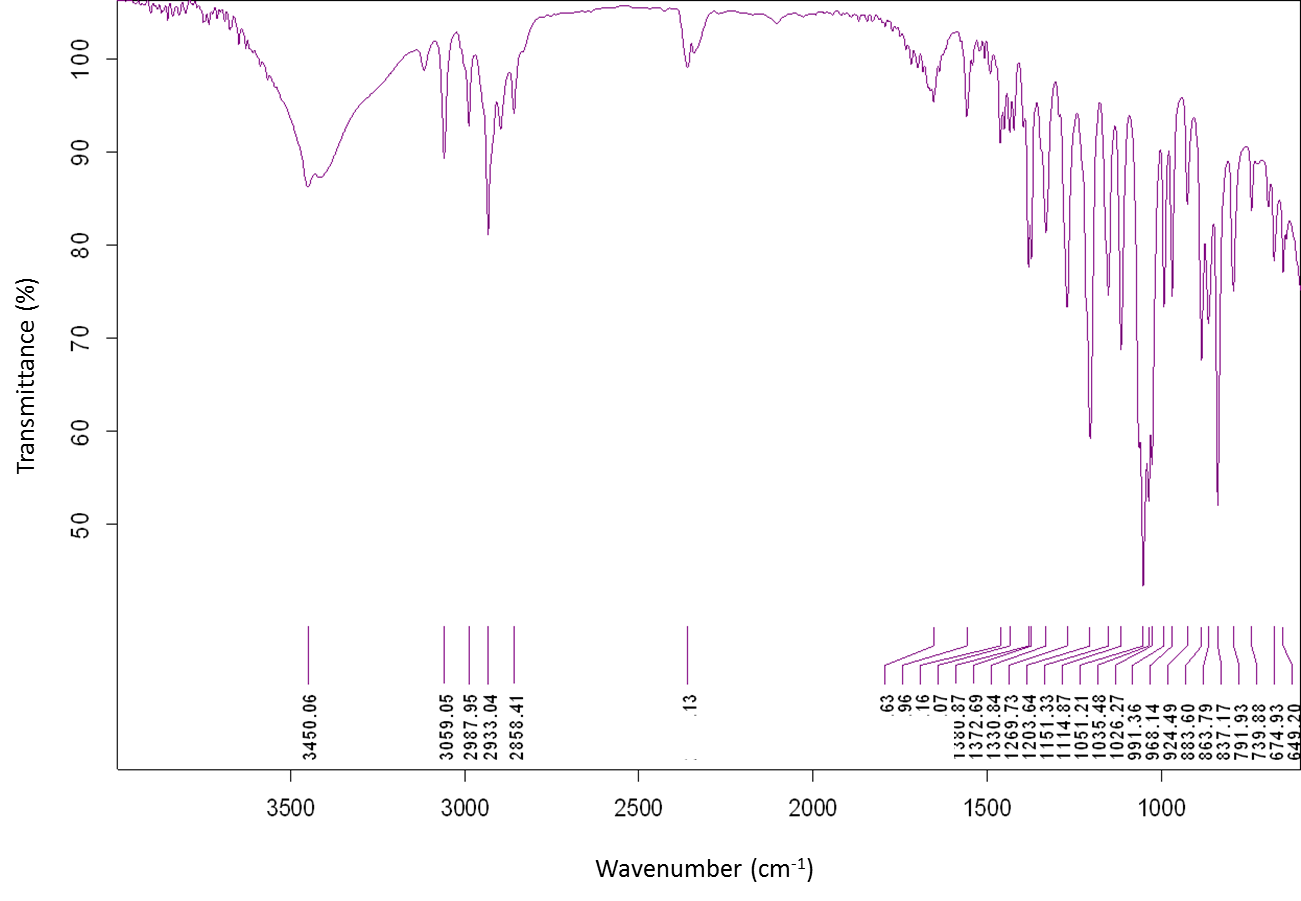


**Figure S61.** IR spectrum of 4-(1-((2,2-dimethyl-1,3-dioxolan-4-yl)methyl)-4-(4-hydroxybutyl)-1*H*-1,2,3-triazole (**4m**).


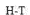


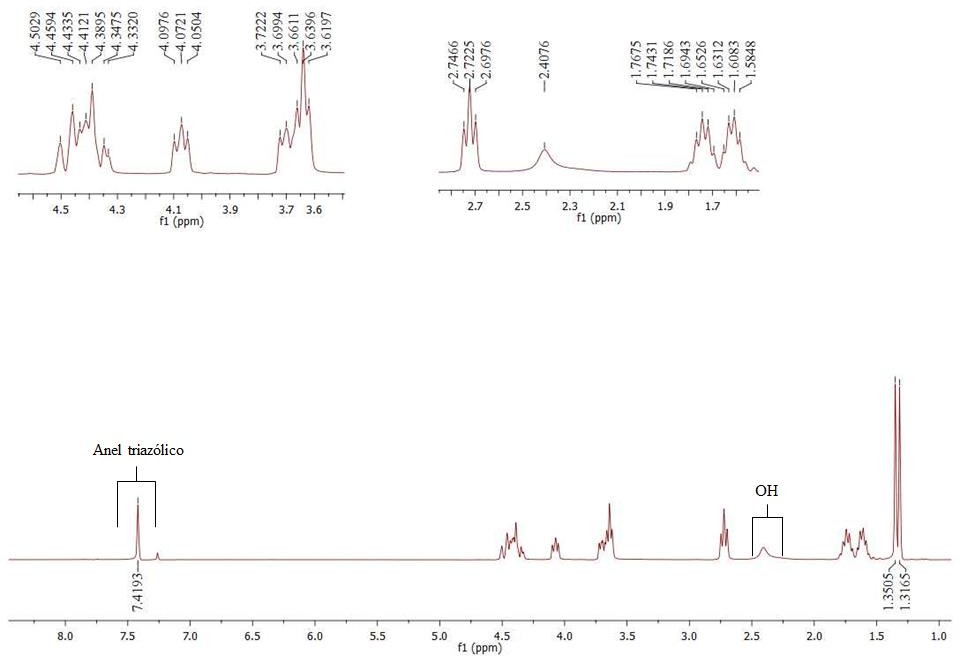

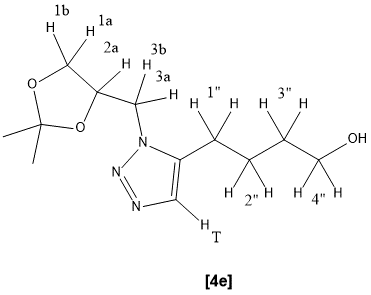


**Figure S62.** ^1^H NMR spectrum (300 MHz, CDCl_3_) of 4-(1-((2,2-dimethyl-1,3-dioxolan-4-yl)methyl)-4-(4-hydroxybutyl)-1*H*-1,2,3-triazole (**4m**).


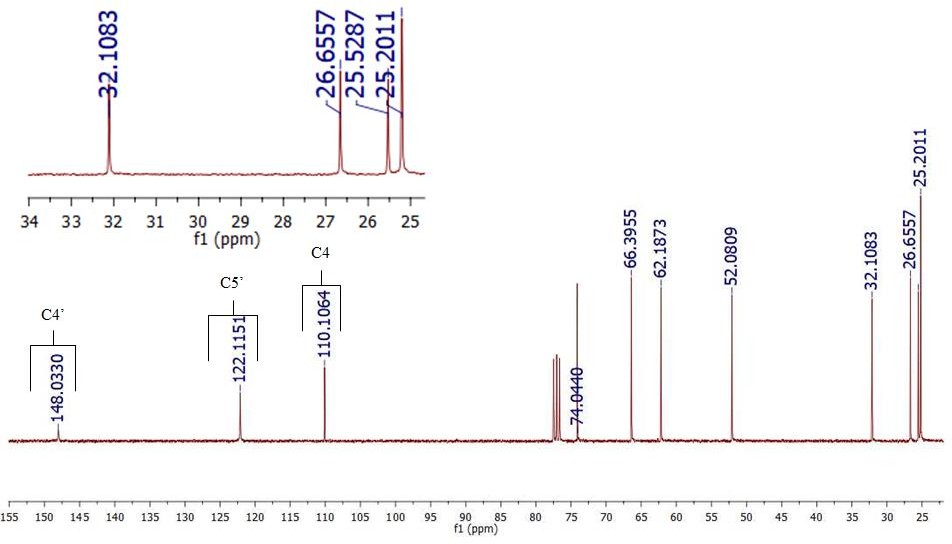

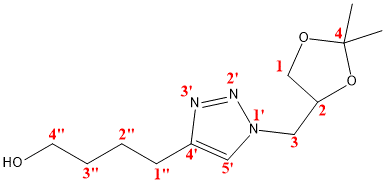


**Figure S63.** ^13^C NMR spectrum (75 MHz, CDCl_3_) of 4-(1-((2,2-dimethyl-1,3-dioxolan-4-yl)methyl)-4-(4-hydroxybutyl)-1*H*-1,2,3-triazole (**4m**).


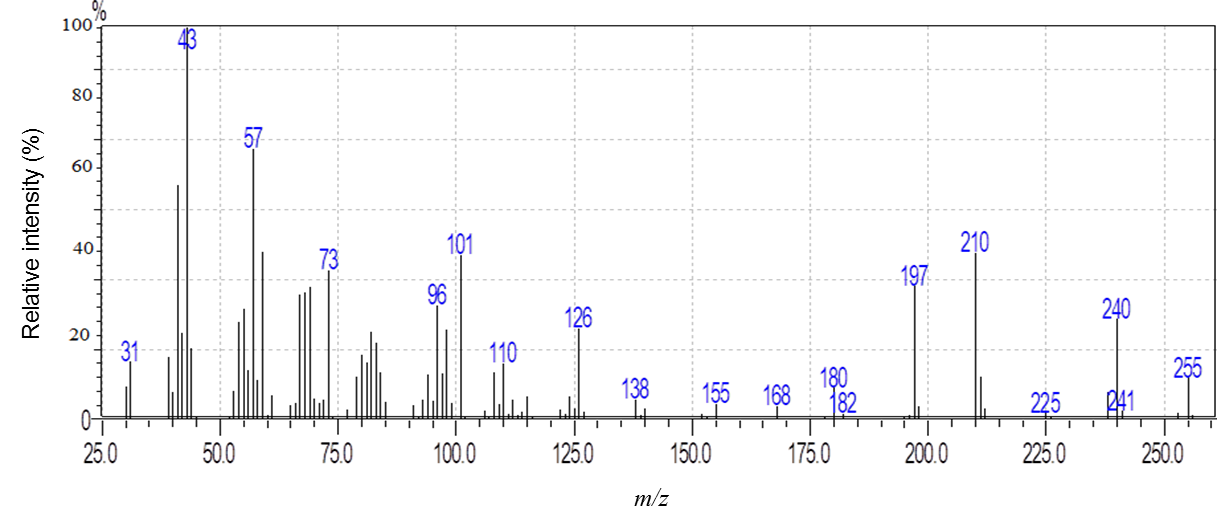


**Figure S64.** MS spectrum of 4-(1-((2,2-dimethyl-1,3-dioxolan-4-yl)methyl)-4-(4-hydroxybutyl)-1*H*-1,2,3-triazole (**4m**).


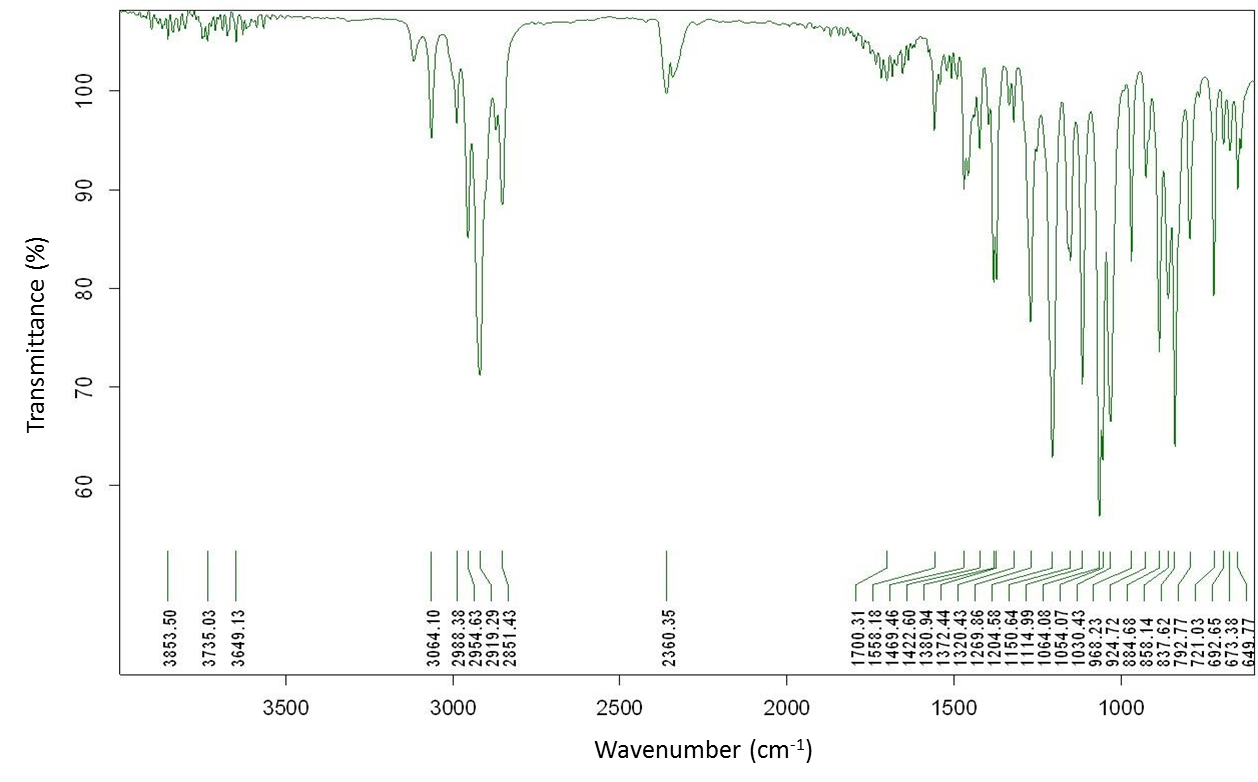


**Figure S65.** IR spectrum of 1-((2,2-dimethyl)1,3-dioxolan-4-yl)-4-heptyl-1*H*-1,2,3-triazole (**4n**).


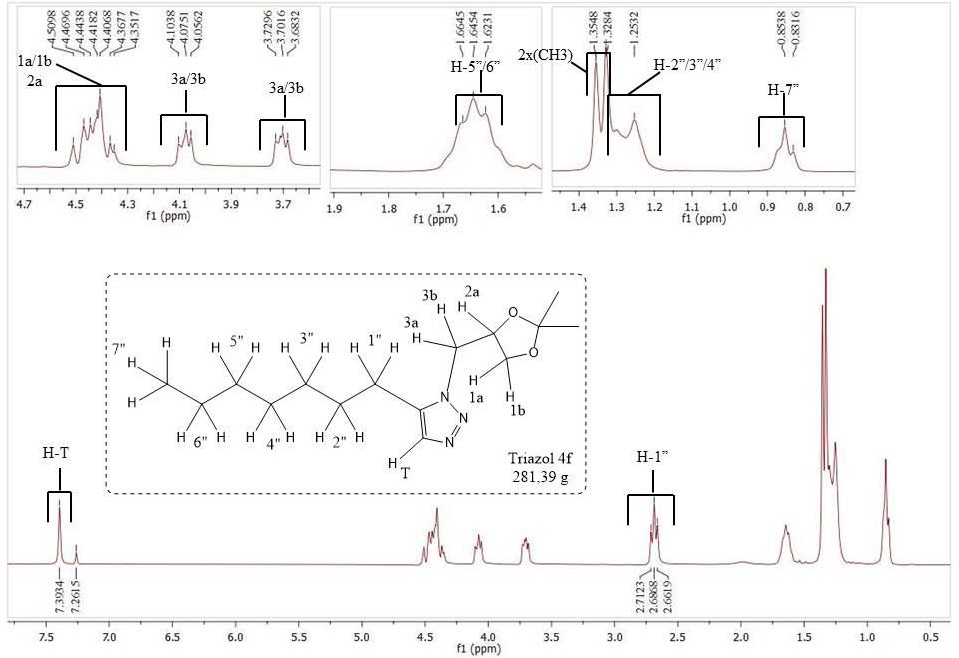


**Figure S66.** ^1^H NMR spectrum (300 MHz, CDCl_3_) of 1-((2,2-dimethyl)1,3-dioxolan-4-yl)-4-heptyl-1*H*-1,2,3-triazole (**4n**).


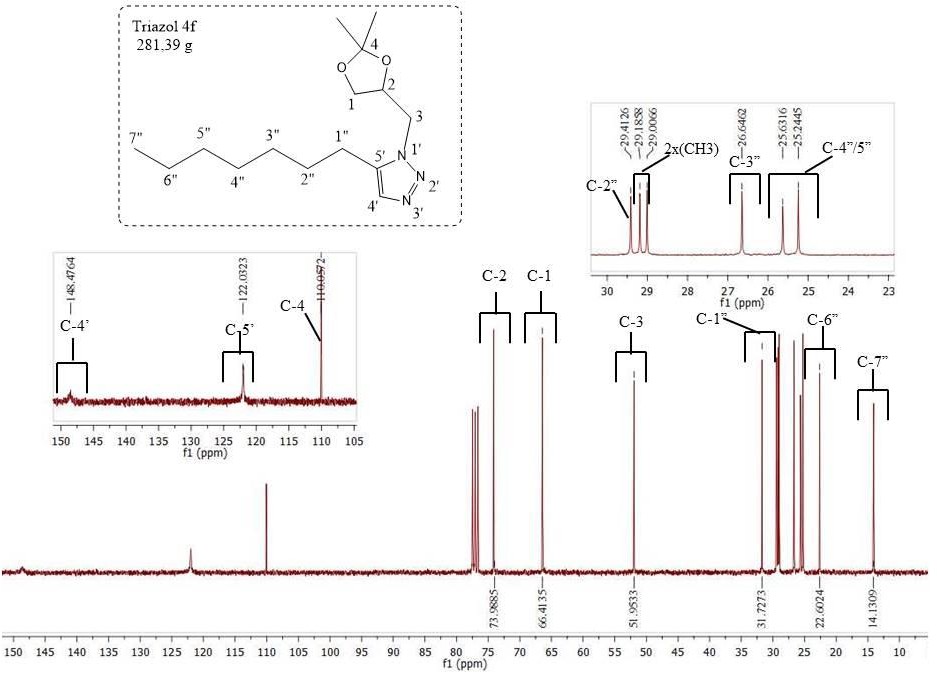


**Figure S67.** ^13^C NMR spectrum (75 MHz, CDCl_3_) of 1-((2,2-dimethyl)1,3-dioxolan-4-yl)-4-heptyl-1*H*-1,2,3-triazole (**4n**).


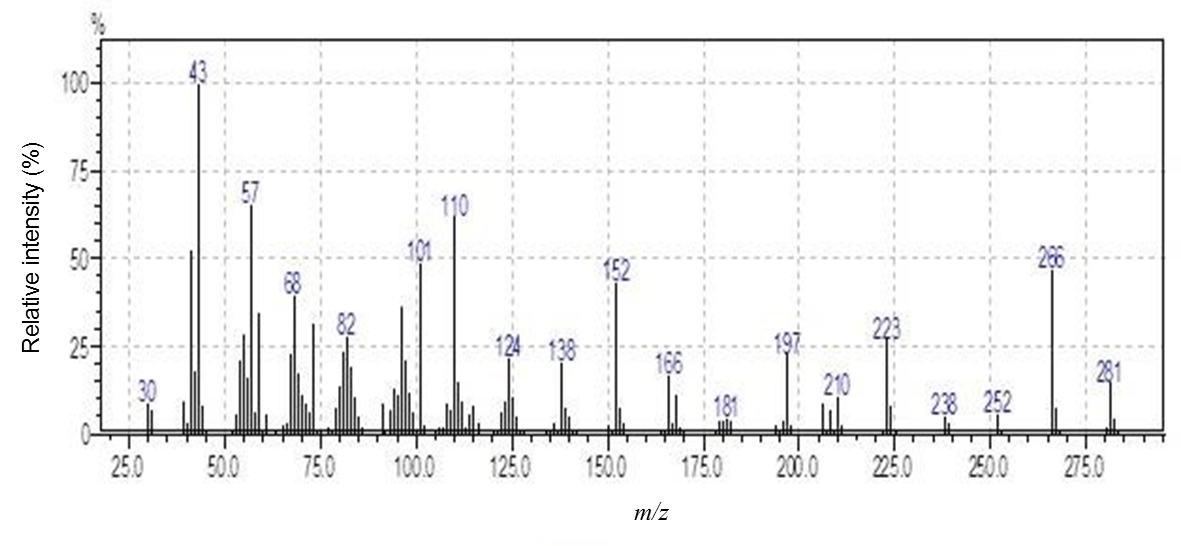


**Figure S68.** MS spectrum of 1-((2,2-dimethyl)1,3-dioxolan-4-yl)-4-heptyl-1*H*-1,2,3-triazole (**4n**).


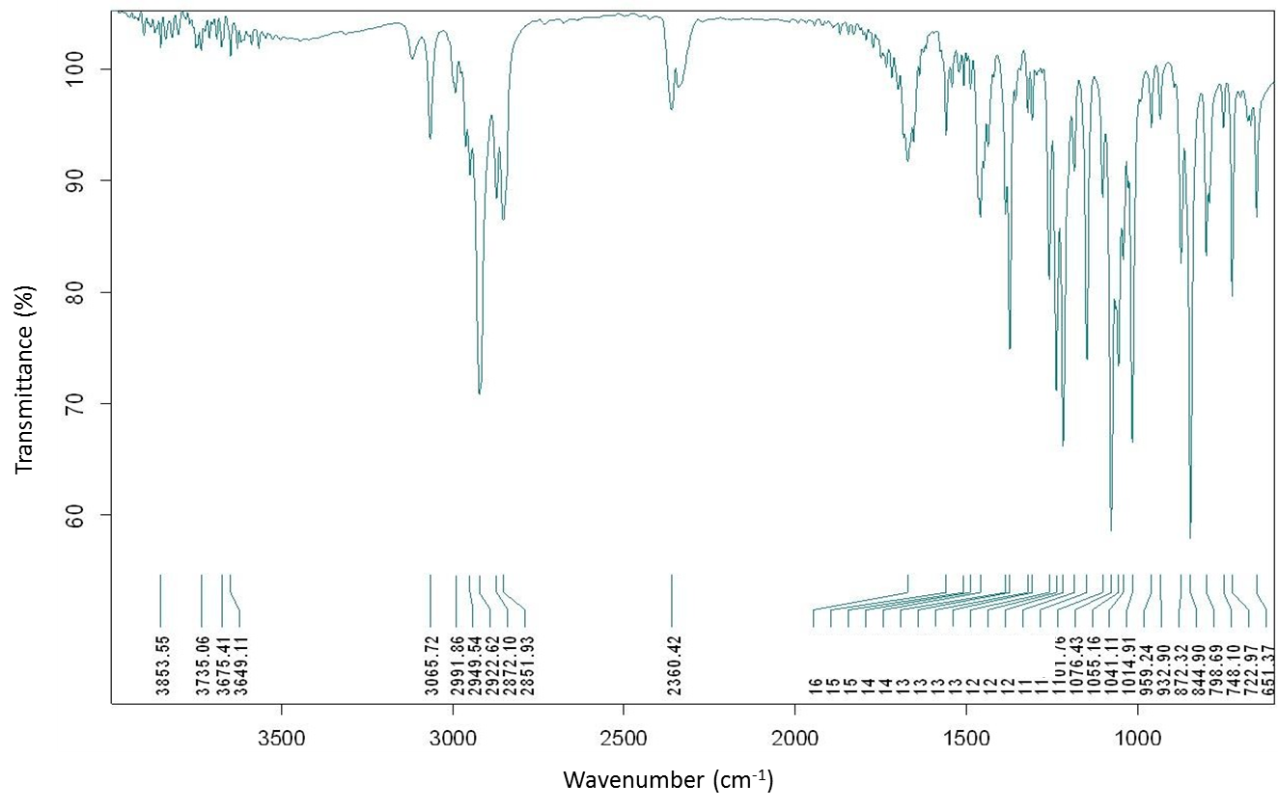


**Figure S69.** IR spectrum of 1-((2,2-dimethyl-1,3-dioxolan-4-yl)methyl)-4-octyl-1*H*-1,2,3-triazole (**4o**).


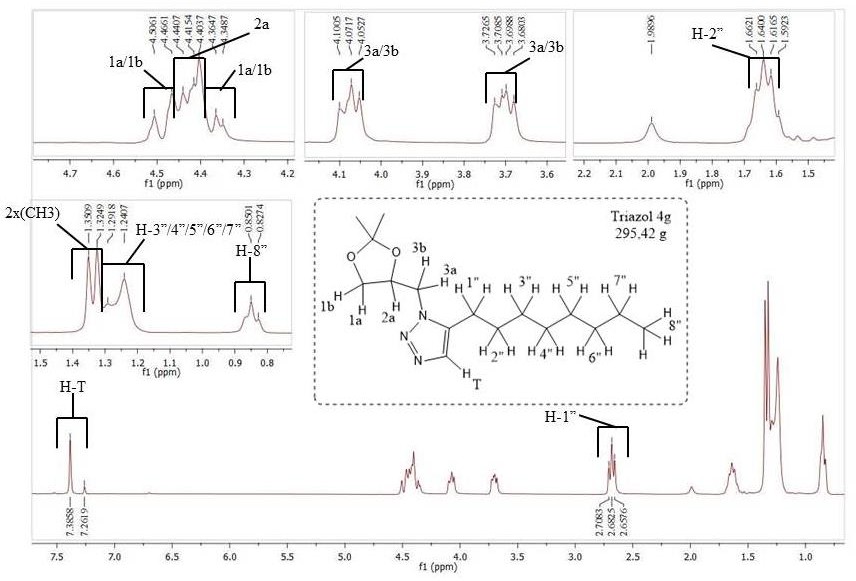


**Figure S70.** ^1^H NMR spectrum (300 MHz, CDCl_3_) of 1-((2,2-dimethyl-1,3-dioxolan-4-yl)methyl)-4-octyl-1*H*-1,2,3-triazole (**4o**).


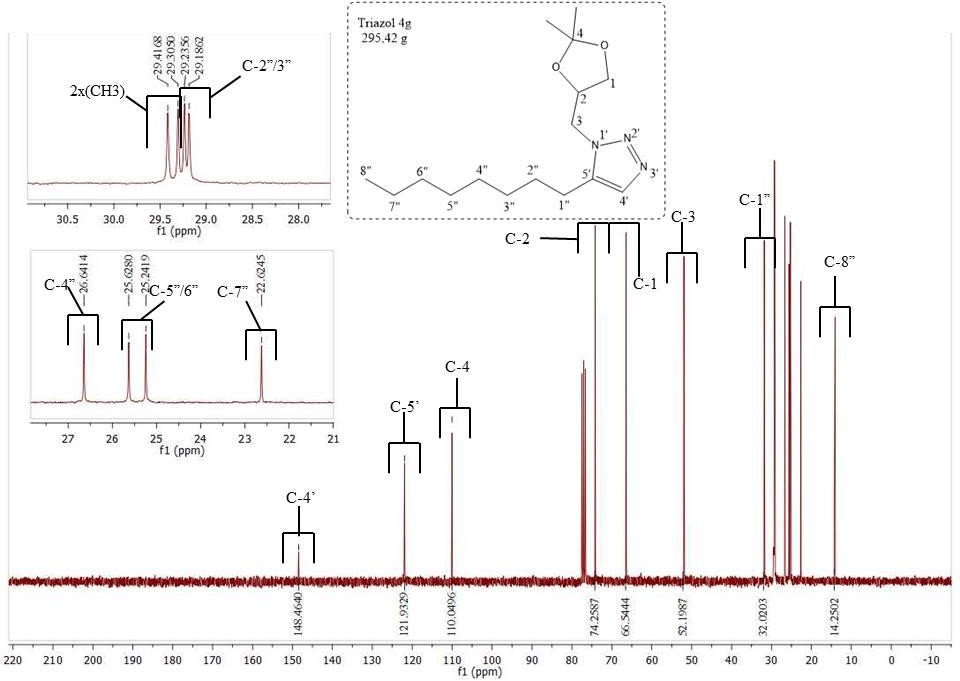


**Figure S71.** ^13^C NMR spectrum (75 MHz, CDCl_3_) of 1-((2,2-dimethyl-1,3-dioxolan-4-yl)methyl)-4-octyl-1*H*-1,2,3-triazole (**4o**).


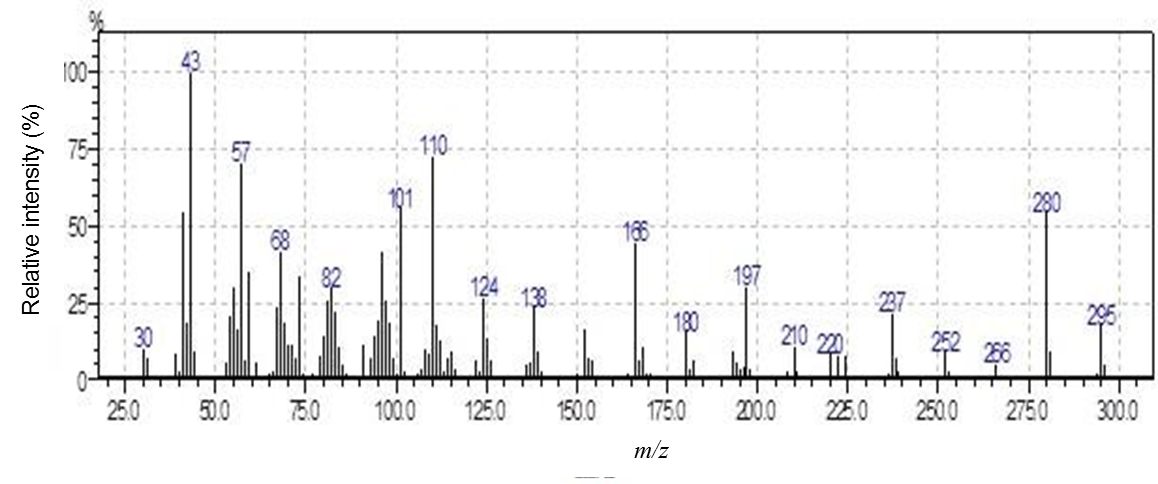


**Figure S72.** MS spectrum of 1-((2,2-dimethyl-1,3-dioxolan-4-yl)methyl)-4-octyl-1*H*-1,2,3-triazole (**4o**).


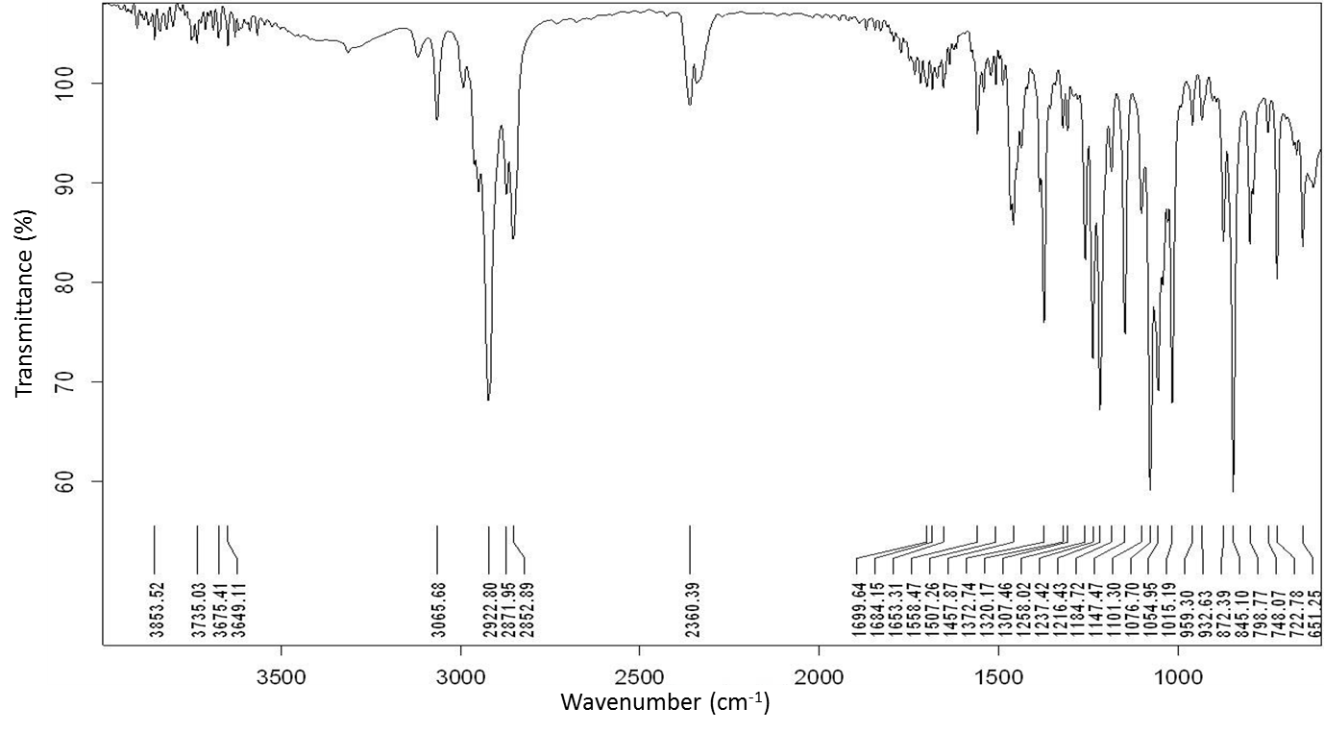


**Figure S73.** IR spectrum of 1-((2,2-dimethyl-1,3-dioxolan-4-yl)methyl)-4-nonyl-1*H*-1,2,3-triazole (**4p**).


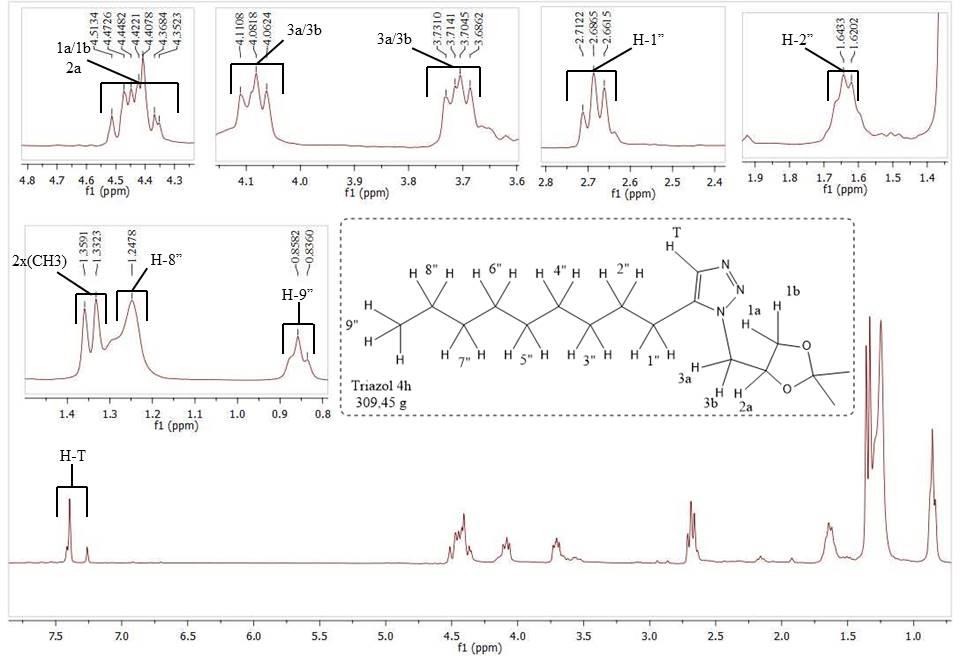


**Figure S74.** ^1^H NMR spectrum (300 MHz, CDCl_3_) of 1-((2,2-dimethyl-1,3-dioxolan-4-yl)methyl)-4-nonyl-1*H*-1,2,3-triazole (**4p**).


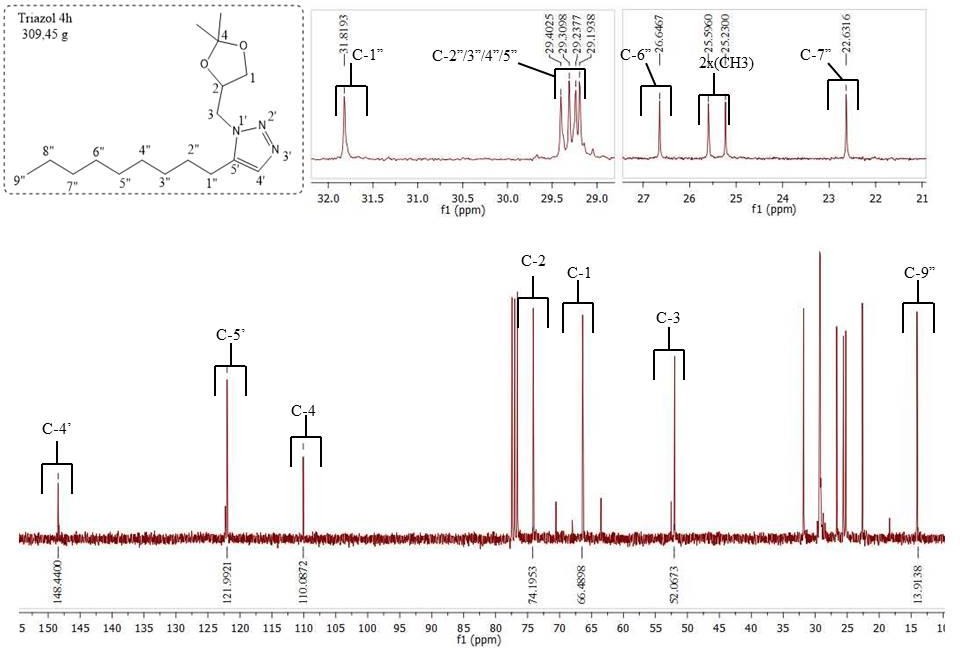


**Figure S75.** ^13^C NMR spectrum (75 MHz, CDCl_3_) of 1-((2,2-dimethyl-1,3-dioxolan-4-yl)methyl)-4-nonyl-1*H*-1,2,3-triazole (**4p**).


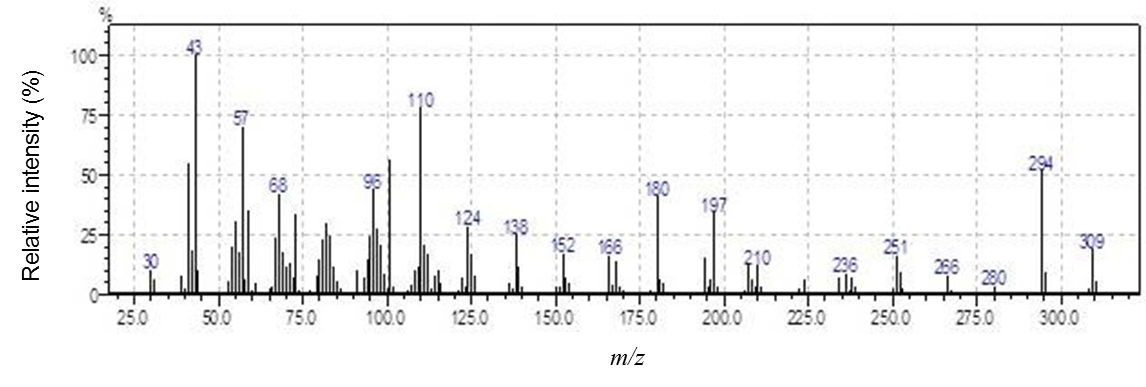


**Figure S76.** MS spectrum of 1-((2,2-dimethyl-1,3-dioxolan-4-yl)methyl)-4-nonyl-1*H*-1,2,3-triazole (**4p**).

**
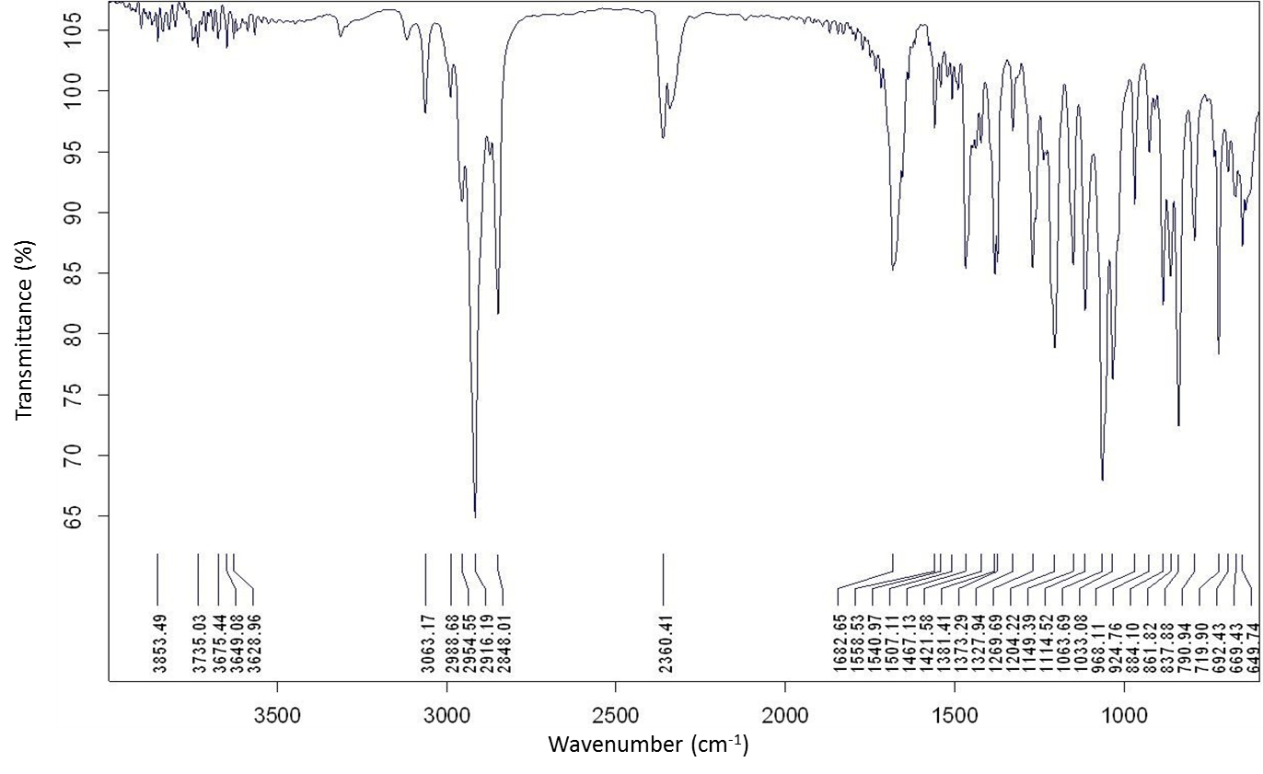
**

**Figure S77.** IR spectrum of 1-((2,2-dimethyl-1,3-dioxolan-4-yl)methyl)-4-decyl-1*H*-1,2,3-triazole (**4q**).


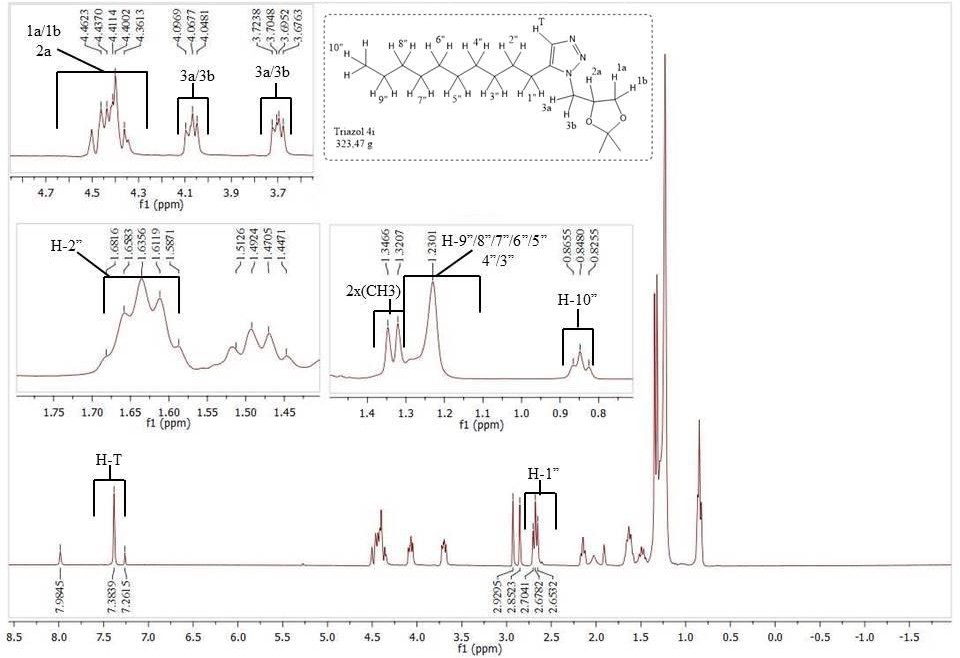


**Figure S78.** ^1^H NMR spectrum (300 MHz, CDCl_3_) of 1-((2,2-dimethyl-1,3-dioxolan-4-yl)methyl)-4-decyl-1*H*-1,2,3-triazole (**4q**).


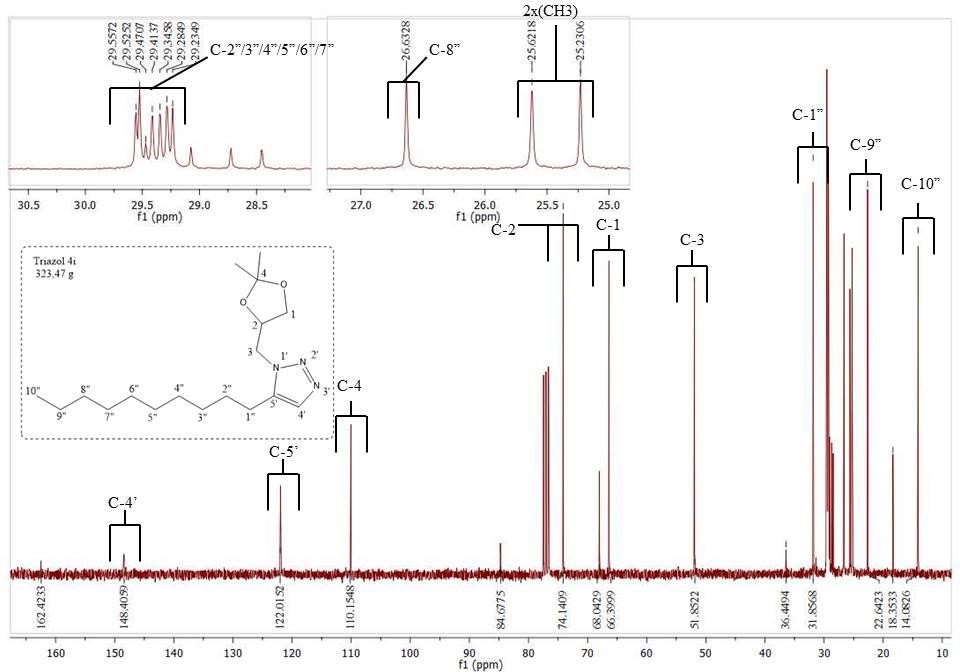


**Figure S79.** ^13^C NMR spectrum (75 MHz, CDCl_3_) of 1-((2,2-dimethyl-1,3-dioxolan-4-yl)methyl)-4-decyl-1*H*-1,2,3-triazole (**4q**).


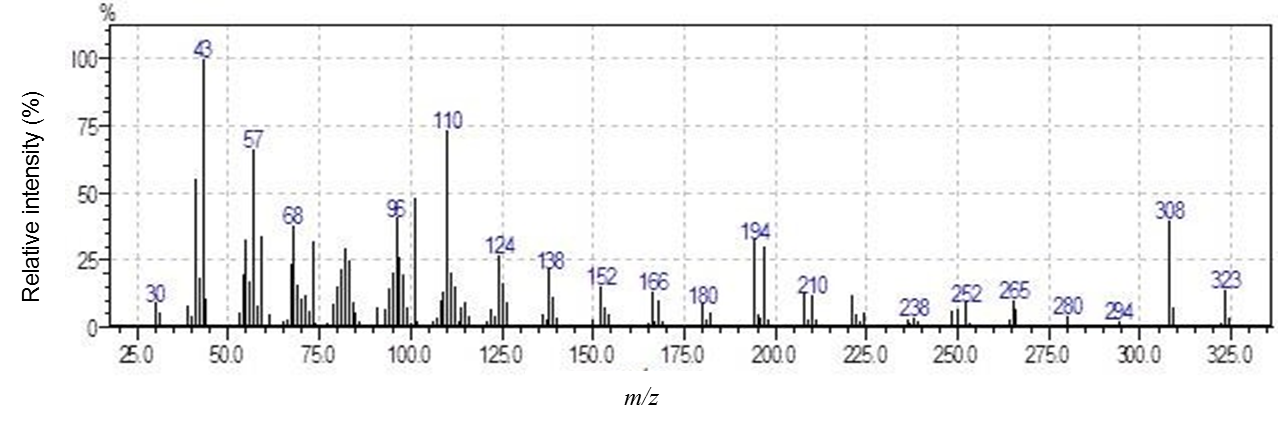


**Figure S80.** MS spectrum of 1-((2,2-dimethyl-1,3-dioxolan-4-yl)methyl)-4-decyl-1*H*-1,2,3-triazole (**4q**).

**2. The structural characterization data of compounds 1, 2, 3, and triazoles 4a−4q.**

*Synthesis of (2,2-dimethyl-1,3-dioxolan-4-yl)methanol (****1****).* Colorless liquid, prepared in 63% yield from the reaction between glycerol (100 mL, 1.36 mol), acetone (100 mL, 1.36 mol), *p*-toluenesulfonic acid (0.080 g, 0.460 mmol) and copper sulfate pentahydrate (10.000 g, 62.65 mmol); TLC (hexane-ethyl acetate 3:1 v v^-1^); R_f_ = 0.35; IR (ATR) –*v*_max_/cm^-1^ 3385, 2937, 1372, 1213, 1156. ^1^H NMR (300 MHz, CDCl_3_) *δ* 1.27 (s, 3H), 1.33 (s, 3H), 2.99 (brs, 1H), 3.49 (dd, 1H, *J* 11.5, 5.2 Hz), 3.58 (dd, 1H, *J* 11.5, 4.2 Hz), 3.66 (dd, 1H, *J* 8.2, 6.6 Hz), 3.94 (dd, 1H, *J* 8.2, 6.6 Hz), 4.09−4.16 (m, 1H). ^13^C NMR (75 MHz, CDCl_3_) *δ* 25.0, 26.4, 62.8, 65.6, 76.0, 109.1. MS (*m/z*, %): 117 ([M−15]+, 38), 101 (22), 72 (10), 57 (25), 43 (100), 31 (12).

*Synthesis of (2,2-dimethyl-1,3-dioxolan-4-yl)methyl-4-methylbenzenesulfonate (****2****).* Colorless liquid, prepared in 75% yield from the reaction between compound **1** (12.520 g, 98.48 mmol), pyridine (50 mL, 640.0 mmol) and 4-toluenesulfonyl chloride (27.000 g, 142.2 mmol); TLC (hexane-ethyl acetate 3:1 v v^-1^); R_f_ = 0.89; IR (ATR) –*v*_max_/cm^-1^ 2995, 2985, 1600, 1365, 1265, 1176, 978. ^1^H NMR (300 MHz, CDCl_3_) *δ* 1.30 (s, 3H,), 1.33 (s, 3H,), 2.45 (s, 3H), 3.76 (dd, 1H, *J* 8.8 Hz, 5.2 Hz), 3.93−4.04 (m, 3H), 4.23−4.31 (m, 1H), 7.34 (d, 2H, *J* 8.2 Hz), 7.79 (d, 2H, *J* 8.2 Hz). ^13^C NMR (75 MHz, CDCl_3_) *δ* 21.6, 25.1, 26.6, 66.1, 69.4, 72.8, 110.0, 127.9, 129.8, 132.4, 145.0. MS (*m/z*, %): 271 ([M −15]+, 90), 173 (10), 155 (76), 101 (86), 91(89), 65 (24), 59 (10), 43 (100), 31(4).

*Synthesis of 4-(azidomethyl)-2,2-dimethyl-1,3-dioxolane (****3****).* Yellow liquid, prepared in 93% yield from the reaction between compound **2** (0.900 g, 3.15 mmol), and sodium azide (1.000 g, 15.73 mmol); TLC (ether-dicloromethane 10:1 v v^-1^); R_f_ = 0.51; IR (ATR) –*v*_max_/cm^-1^ 3497, 2932, 2102, 1736, 1662, 1439, 1386, 1244, 1091, 1046, 659. ^1^H NMR (300 MHz, CDCl_3_) *δ* 1.32 (s, 3H), 1.42 (s, 3H), 3.24 (dd, 1H, *J* 12.6, 5.4 Hz), 3.35 (dd, 1H, *J* 12.6, 4.7 Hz), 3.72 (dd, 1H, *J* 8.5, 5.7 Hz), 4.00 (dd, 1H, *J* 8.5, 6.3 Hz), 4.19*−*4.26 (m, 1H). ^13^C NMR (75 MHz, CDCl_3_) *δ* 25.4, 26.6, 53.0, 66.7, 74.7, 110.2. MS (*m/z*, %): 142 ([M *−* 15]+, 39), 101 (61), 83 (4), 72 (10), 59 (12), 43 (100), 31 (5).

*Synthesis of (1-((2,2-dimethyl-1,3-dioxolan-4-yl)methyl)-1H-1,2,3-triazol-4-yl)methanol (****4a****).* Yellow liquid, prepared in 76% yield from the reaction between prop-2-yn-1-ol (0.538 g, 9.6 mmol) and azide **3** (1.000 g, 6.4 mmol); TLC (ether-dicloromethane 10:1 v v^-1^); R_f_ = 0.32; IR (ATR) –*v*_max_/cm^-1^ 3355, 2972, 1472, 1365, 1202, 1040, 910, 833; ^1^H NMR (300 MHz, CDCl_3_) *δ* 1.32 (s, 3H), 1.37 (s, 3H), 3.44 (s, 1H), 3.72 (dd, 1H, *J* 8.8, 5.5 Hz), 4.10 (dd, 1H, *J* 8.8, 6.0 Hz), 4.38 (dd, 1H, *J* 12.9, 6.0 Hz), 4.41−4.48 (m, 1H), 4.53 (dd, 1H, *J* 12.9, 3.3 Hz), 4.75 (s, 2H), 7.68 (s, 1H). ^13^C NMR (75 MHz, CDCl_3_) *δ* 25.3, 26.9, 52.5, 56.3, 66.6, 74.4, 110.6, 123.6, 148.0. MS (*m/z*, %): 213 ([M+], 1), 198 ([M − 15]+, 42), 155 (49), 138 (18), 113 (18), 101 (44), 83 (10), 73 (23), 57 (23), 43 (100), 31 (12).

*Synthesis of 2-(1-((2,2-dimethyl-1,3-dioxolan-4-yl)methyl)-1H-1,2,3-triazol-4-yl)ethanol (****4b****).* Yellow liquid, prepared in 82% yield from the reaction between but-3-yn-1-ol (0.673 g, 9.6 mmol) and azide **3** (1.000 g, 6.4 mmol); TLC (ether-dicloromethane 10:1 v v^-1^); R_f_ = 0.35; IR (ATR) –*v*_max_/cm^-1^ 3384, 2928, 1648, 1554, 1457, 1373, 1217, 1151, 1116, 1051, 968, 880, 831. ^1^H NMR (300 MHz, CDCl_3_) *δ* 1.30 (s, 3H), 1.35 (s, 3H), 2.91 (t, 2H, *J* 6.3 Hz), 3.40 (s, 1H), 3.70 (dd, 1H, *J* 8.8, 5.5 Hz), 3.88 (t, 2H, *J* 6.3 Hz), 4.08 (dd, 1H, *J* 8.8, 6.0 Hz), 4.35 (dd, 1H, *J* 12.6, 5.4 Hz), 4.39−4.45 (m, 1H), 4.48 (dd, 1H, *J* 12.6, 3.3 Hz), 7.52 (s, 1H). ^13^C NMR (75 MHz, CDCl_3_) *δ* 25.4, 26.9, 28.9, 52.5, 61.6, 66.6, 74.3, 110.4, 123.3, 145.4. MS (*m/z*, %): 228 ([M+], 2), 227 (M+, 2), 212 ([M − 15]+, 35), 197 (8), 169 (65), 152 (16), 127 (15), 110 (20), 101 (25), 68 (37), 57 (62), 43 (100), 32 (50).

*Synthesis of 3-(1-((2,2-dimethyl-1,3-dioxolan-4-yl)methyl)-1H-1,2,3-triazol-4-yl)propan-1-ol (****4c****).* Yellow liquid, prepared in 68% yield from the reaction between pent-4-yn-1-ol (0.808 g, 9.6 mmol) and azide **3** (1.000 g, 6.4 mmol); TLC (ether-dicloromethane 10:1 v v^-1^); Rf = 0.37; IR (ATR) –*v*_max_/cm^-1^ 3380, 2938, 1648, 1552, 1456, 1373, 1257, 1216, 1151, 1058, 969, 880, 831. ^1^H NMR (300 MHz, CDCl_3_) *δ* 1.30 (s, 3H), 1.34 (s, 3H), 1.88 (quint, 2H, *J* 7.4, 6.3 Hz), 2.78 (t, 2H, *J* 7.4 Hz), 3.41(s, 1H), 3.69 (t, 2H, *J* 6.3 Hz), 3.70 (dd, 1H, *J* 8.7, 5.7 Hz), 4.07 (dd, 1H, *J* 8.7, 5.7 Hz),4.35 (dd, 1H, *J* 12.6, 5.2 Hz), 4.38−4.45 (m, 1H), 4.48 (dd, 1H, *J* 12.6, 3.2 Hz), 7.44 (s, 1H). ^13^C NMR (75 MHz, CDCl_3_) *δ* 22.1, 25.4, 26.7, 32.2, 52.4, 61.7, 66.6, 74.3, 110.4, 122.6, 147.8. MS (*m/z*, %): 242 ([M+], 2), 241 ([M+], 4), 226 ([M − 15]+, 31), 211 (12), 197 (9), 183 (39), 166 (11), 112 (46), 101 (28), 83 (223), 68 (25), 57 (61), 43 (100), 41 (41), 31 (18).

*Synthesis of 1-(1-((2,2-dimethyl-1,3-dioxolan-4-yl)methyl)-1H-1,2,3-triazol-4-yl)ethanol (****4d****).* Yellow liquid, prepared in 75% yield from the reaction between but-3-yn-2-ol (0.673 g, 9.6 mmol) and azide **3** (1.000 g, 6.4 mmol); TLC (ether-dicloromethane 10:1 v v^-1^); R_f_ = 0.37; IR (ATR) –*v*_max_/cm^-1^ 3385, 2985, 1373, 1217, 1149, 1066, 892, 830. ^1^H NMR (300 MHz, CDCl_3_) *δ* 1.33 (s, 3H), 1.37 (s, 3H), 1.58 (d, *J* 6.6 Hz), 3.50 (s, 1H), 3.73 (dd, 1H, *J* 8.8, 5.4 Hz), 4.10 (dd, 1H, *J* 8.8, 5.7 Hz), 4.39 (dd, 1H, *J* 12.9, 5.5 Hz), 4.42−4.49 (m, 1H), 4.52 (dd, 1H, *J* 12.9, 3.0 Hz), 5.07 (q, 1H, *J* 6.6 Hz), 7.61 (s, 1H). ^13^C NMR (75 MHz, CDCl_3_) *δ* 23.0, 25.1, 26.6, 52.2, 62.9, 66.3, 74.0, 110.1, 121.4, 152.7. MS (*m/z*, %): 228 ([M+], 1), 227 ([M+], 1), 212 ([M−15]+, 36), 169 (38), 152 (16), 127 (12), 101 (36), 73 (23), 57 (29), 43 (100), 31 (8).

*Synthesis of 1-(1-((2,2-dimethyl-1,3-dioxolan-4-yl)methyl)-1H-1,2,3-triazol-4-yl)propan-2-ol (****4e****).* Yellow liquid, prepared in 70% yield from the reaction between pent-4-yn-2-ol (0.808 g, 9.6 mmol) and azide **3** (1.000 g, 6.4 mmol); TLC (ether-dicloromethane 10:1 v v^-1^); R_f_ = 0.29; IR (ATR) –*v*_max_/cm^-1^ 3384, 2985, 1648, 1552, 1457, 1373, 1216, 1151, 1117, 1065, 1045, 968, 941, 880, 831. ^1^H NMR (300 MHz, CDCl_3_) *δ* 1.26 (d, 3H, *J* 6.3 Hz), 1.33 (s, 3H), 1.37 (s, 3H), 2.72−292 (m, 1H), 3.46 (s, 1H), 3.74 (dd, 1H, *J* 8.8, 5.7 Hz), 4.10 (dd, 1H, *J* 8.8, 5.8 Hz), 4.15−4.17 (m, 2H), 4.39 (dd, 1H, *J* 12.6, 5.7 Hz), 4.42−4.49 (m, 1H), 4.52 (dd, 1H, *J* 12.6, 3.3 Hz), 7.52 (s, 1H). ^13^C NMR (75 MHz, CDCl_3_) *δ* 23.1, 25.4, 26.9, 35.0, 52.5, 66.6, 67.3, 74.3, 110.4, 123.4, 145.5. MS (*m/z*, %): 242 ([M+], 1), 226 ([M − 15]+, 31), 197 (94), 183 (12), 166 (11), 139 (22), 115 (26), 101 (22), 83 (26),68 (50), 57 (69), 43 (100).

*Synthesis of 2-(1-((2,2-dimethyl-1,3-dioxolan-4-yl)methyl)-1H-1,2,3-triazol-4-yl)butan-2-ol (****4f****).* Yellow liquid, prepared in 84% yield from the reaction between 3-methylpent-1-yn-3-ol (0.942 g, 9.6 mmol) and azide **3** (1.000 g, 6.4 mmol); TLC (ether-dicloromethane 10:1 v v^-1^); R_f_ = 0.42; IR (ATR) –*v*_max_/cm^-1^ 3407, 2979, 1457, 1373, 1217, 1151, 1046, 993, 919, 831. ^1^H NMR (300 MHz, CDCl_3_) *δ* 0.84 (t, 3H, *J* 7.4 Hz), 1.33 (s, 3H), 1.35 (s, 3H), 1.56 (d, 3H, *J* 3.6 Hz), 1.89 (sept, 2H, *J* 7.4 Hz), 3.46 (s, 1H), 3.71 (dd, 1H, *J* 8.7, 5.5 Hz), 4.09 (dd, 1H, *J* 8.7, 6.0 Hz), 4.39 (dd, 1H, *J* 12.9, 5.2 Hz), 4.42−4.49 (m, 1H), 4.51 (dd, 1H, *J* 12.9, 4.4 Hz), 7.55 (s, 1H). ^13^C NMR (75 MHz, CDCl_3_) *δ* 8.5, 25.4, 26.7, 28.2, 36.1, 52.3, 66.6, 71.4, 74.4, 110.4, 121.5, 154.7. MS (*m/z*, %): 256 ([M+], 1), 240 ([M − 15]+, 25), 226 (95), 180 (9), 125 (40), 101 (20), 84 (22), 57 (45), 43 (100), 31 (9).

*Synthesis of 1-(1-((2,2-dimethyl-1,3-dioxolan-4-yl)methyl)-1H-1,2,3-triazol-4-yl)cyclohexanol (****4g****).* White solid, prepared in 65% yield from the reaction between 1-ethynylcyclohexan-1-ol (1.192 g, 9.6 mmol) and azide **3** (1.000 g, 6.4 mmol); TLC (ether-dicloromethane 10:1 v v^-1^); R_f_ = 0.26; m.p. 85−88 ºC; IR (ATR) –*v*_max_/cm^-1^ 3290, 2933, 1448, 1371, 1223, 1162, 1071, 968, 898, 828. ^1^H NMR (300 MHz, CDCl_3_) *δ* 1.34 (s, 3H), 1.36 (s, 3H), 1.52−1.99 (m, 10H), 3.48 (s, 1H), 3.73 (dd, 1H, *J* 8.5, 5.4 Hz), 4.11 (dd, 1H, *J* 8.5, 6.0 Hz), 4.38−4.55 (m, 3H), 7.58 (s, 1H). ^13^C NMR (75 MHz, CDCl_3_) *δ* 21.9, 23.1, 25.3, 26.6, 38.1, 39.7, 52.1, 66.4, 69.5, 72.0, 74.0, 110.1, 121.0, 152.0. MS (*m/z*, %): 281 ([M+], 33), 263 (32), 248 (34), 238 (18), 210 (19), 176 (12), 152 (19), 134 (39), 121 (18), 101 (27), 79 (35), 68 (33), 57 (65), 43 (100), 31 (12).

*Synthesis of 2-(1-((2,2-dimethyl-1,3-dioxolan-4-yl)methyl)-1H-1,2,3-triazol-4-yl)propan-2-ol (****4h****).*  Yellow liquid, prepared in 73% yield from the reaction between 2-methylbut-3-yn-2-ol (0.808 g, 9.6 mmol) and azide **3** (1.000 g, 6.4 mmol); TLC (ether-dicloromethane 10:1 v v^-1^); R_f_ = 0.37; IR (ATR) –*v*_max_/cm^-1^ 3388, 2983, 1457, 1374, 1212, 1150, 1052, 960, 830. ^1^H NMR (300 MHz, CDCl_3_) *δ* 1.32 (s, 3H), 1.34 (s, 3H), 1.61 (s, 3H), 1.62 (s, 3H), 2.98 (s, 1H), 3.72 (dd, 1H, *J* 8.8, 5.2Hz), 4.09 (dd, 1H, *J* 8.8, 5.7 Hz), 4.38 (dd, 1H, *J* 12.6, 5.2 Hz), 4.41−4.47 (m, 1H), 4.51 (dd, 1H, *J* 12.6, 3.3 Hz), 7.57 (s, 1H). ^13^C NMR (75 MHz, CDCl_3_) *δ* 25.1, 26.6, 30.2, 30.4, 52.1, 66.3, 68.3, 73.9, 110.1, 120.6, 155.7. MS (*m/z*, %): 241 ([M+], 1), 226 ([M − 15]+, 49), 208 (11), 183 (24), 166 (10), 101 (35), 94 (24), 73 (21), 57 (38), 43 (100), 31 (12).

*Synthesis of 1-((2,2-dimethyl-1,3-dioxolan-4-yl)methyl)-4-propyl-1H-1,2,3-triazole (****4i****).* Yellow liquid, prepared in 85% yield from the reaction between pent-1-yne (1.300 g, 19.2 mmol) and azide **3** (2.000 g, 12.8 mmol); TLC (ether-dicloromethane 10:1 v v^-1^); R_f_ = 0.61; IR (ATR) –*v*_max_/cm^-1^ 2984, 1372, 1216, 1064, 832, 798; ^1^H NMR (300 MHz, CDCl_3_) *δ* 0.93 (t, 3H, *J* 9.0 Hz), 1.31 (s, 3H), 1.34 (s, 3H), 1.67 (sept, 2H, *J* 9.0 Hz), 2.66 (t, 2H, *J* 9.0 Hz), 3.70 (dd, 1H, *J* 9.0, 6.0 Hz), 4.07 (dd, 1H, *J* 9.0, 6.0 Hz), 4.37 (dd, 1H, *J* 12.0, 6.0 Hz), 4.40−4.45 (m, 1H), 4.48 (dd, 1H, *J* 12.0, 3.0 Hz), 7.38 (s,1H); ^13^C NMR (75 MHz, CDCl_3_) *δ* 14.0, 22.9, 25.4, 26.8, 27.8, 52.1, 66.6, 74.3, 110.2, 122.2, 148.3; MS (*m/z*, %) 225 ([M+], 4), 210 ([M − 15]+, 3), 168 (11), 167 (46), 150 (13), 125 (13), 110 (50), 101 (30), 82 (1), 73 (27), 68 (35), 57 (57), 43 (100), 32 (17).

*Synthesis of 1-((2,2-dimethyl-1,3-dioxolan-4-yl)methyl)-4-butyl-1H-1,2,3-triazole (****4j****).* White solid, prepared in 93% yield from the reaction between hex-1-yne (1.600 g, 19.2 mmol) and azide **3** (2.000 g, 12.8 mmol); TLC (ether-dichloromethane 10:1 v v^-1^); R_f_ = 0.60; m.p. 52−53 ºC; IR (ATR) –*v* max/cm^−1^ 3067, 2927, 1312, 1236, 1183, 1014, 844, 798, 648; ^1^H NMR (300 MHz, CDCl_3_) *δ* 0.93 (t, 3H, *J* 9.0 Hz), 1.34 (s, 3H), 1.37 (s, 3H), 1.38 (sept, 2H, *J* 9.0 Hz), 1.68 (quint, 2H, *J* 9.0 Hz), 2.71 (t, 2H, *J* 9.0 Hz), 3.77 (dd, 1H, *J* 9.0, 6.0 Hz), 4.10 (dd, 1H, *J* 9.0, 6.0 Hz), 4.40 (dd, 1H, *J* 12.0, 6.0 Hz), 4.40−4.45 (m, 1H), 4.51 (dd, 1H, *J* 12.0, 6.0 Hz), 7.40 (s, 1H);^13^C NMR (75 MHz, CDCl_3_) *δ* 14.0, 22.5, 25.5, 25.5, 26.8, 31.7, 52.2, 66.6, 74.4, 110.3, 122.1, 148.7; MS (*m/z*, %) 239 ([M+], 6), 124 ([M − 15]+, 25), 224 (40), 181 (48), 164 (11), 139 (16), 110 (66), 101 (38), 82 (27), 68 (41), 57 (74), 43 (100), 31 (10).

*Synthesis of 1-((2,2-dimethyl-1,3-dioxolan-4-yl)methyl)-4-pentyl-1H-1,2,3-triazole (****4k****).* White solid, prepared in 87% yield from the reaction between hep-1-yne (1.800 g, 19.2 mmol) and azide **3** (2.000 g, 12.8 mmol); TLC (ether-dichloromethane 10:1 v v^−1^); R_f_ = 0.60; m.p. 32−33 ºC; IR (ATR) –*v*_max_ /cm^−1^ 2968, 2926,1380, 1269, 1204, 1062, 1032, 884, 836; ^1^H NMR (300 MHz,CDCl_3_) *δ* 0.88 (t, 3H, *J* 9.0 Hz), 1.33 (s, 3H), 1.36 (s, 3H), 1.37 (sept, 4H, *J* 9.0 Hz), 1.65 (quint, 2H, *J* 9.0 Hz), 2.70 (t, 2H, *J* 9.0 Hz), 3.71 (dd, 1H, *J* 9.0, 6.0 Hz), 4.09 (dd, 1H, *J* 9.0, 6.0 Hz), 4.39 (dd, 1H, *J* 12.0, 9.0 Hz), 4.38−4.46 (m,1H), 4.50 (dd, 1H, *J* 12.0, 9.0 Hz), 7.39 (s, 1H); ^13^C NMR (75 MHz, CDCl_3_) *δ* 14.2, 22.6, 25.5, 25.8, 26.9, 29.3, 31.6, 52.1, 66.6, 74.3, 110.3, 122.1, 148.7; MS (*m/z*, %) 253 ([M+], 12), 238 ([M − 15]+, 50), 195 (47), 178 (11), 138 (27), 124 (69), 110 (36), 101 (49), 73 (30), 57 (73), 43 (100), 30 (10).

*Synthesis of 1-((2,2-dimethyl-1,3-dioxolan-4-yl)methyl)-4-hexyl-1H-1,2,3-triazole (****4l****).* White solid, prepared in 75% yield from the reaction between oct-1-yne (2.110 g, 19.2 mmol) and azide **3** (2.000 g, 12.8 mmol); TLC (ether-dichloromethane 10:1 v v^-1^); R_f_ = 0.68; m.p. 58−60 ºC; IR (ATR) –*v*_max_/cm^−1^ 2871, 1372, 1216, 1147, 1076, 1014, 844, 798; ^1^H NMR (300 MHz, CDCl_3_) *δ* 0.86 (t, 3H, *J* 9.0 Hz), 1.30 (sept, 6H, *J* 9.0 Hz), 1.33 (s, 3H), 1.35 (s, 3H), 1.64 (quint, 2H, *J* 9.0 Hz), 2.69 (t, 2H, *J* 9.0 Hz), 3.70 (dd, 1H, *J* 9.0, 6.0 Hz), 4.07 (dd, 1H, *J* 9.0, 6.0 Hz), 4.37 (dd, 1H, *J* 12.0, 6.0 Hz), 4.40−4.46 (m, 1H), 4.49 (dd, 1H, *J* 12.0, 6.0 Hz), 7.39 (s, 1H); ^13^C NMR (75 MHz, CDCl_3_) *δ* 14.2, 22.7, 25.4, 25.8, 26.8, 29.1, 29.6, 31.7, 52.1, 66.6, 74.3, 110.3, 122.1, 148.6; MS (*m/z*, %) 267 ([M+], 15), 252 ([M − 15]+, 52), 209 (40), 192 (10), 168 (9), 152 (20), 138 (67), 124 (22), 101 (52), 96 (36), 68 (39), 57 (71), 43 (100), 30 (10).

*Synthesis of 4-(1-((2,2-dimethyl-1,3-dioxolan-4-yl)methyl)-4-(4-hydroxybutyl)-1H-1,2,3-triazole (****4m****).* Yellow solid, prepared in 81% yield from the reaction between hex-5-yn-1-ol (1.400 g, 14.4 mmol) and azide **3** (1.500 g, 9.60 mmol); TLC (ether-dichloromethane 10:1 v v^-1^); R_f_ = 0.15; m.p. 57−58 ºC; IR (ATR) *̅v*_max_/cm^−1^ 3450, 3059, 2933, 1269, 1203, 1051, 883, 837, 791; ^1^H NMR (300 MHz, CDCl_3_) *δ* 1.32 (s, 3H), 1.35 (s, 3H), 1.61 (quint, 2H, *J* 12.0 Hz) 1.74 (quint, 2H, *J* 9.0 Hz), 2.41 (s, 1H, OH), 2.72 (t, 2H, *J* 9.0 Hz), 3.66 (dd, 1H, *J* 9.0, 6.0 Hz), 4.08 (dd, 1H, *J* 9.0, 6.0 Hz), 4.34 (dd, 1H, *J* 12.0, 6.0 Hz), 4.35−4.45 (m,1H), 4.48 (dd, 1H, *J* 12.0, 6.0 Hz), 7.42 (s, 1H); ^13^C NMR (75 MHz, CDCl_3_) *δ* 25.2, 25.5, 26.6, 32.1, 52.0, 62.1, 66.4, 74.1, 110.1, 122.1, 148.0; MS (*m/z*, %) 255 ([M+], 3), 240 ([M − 15]+, 8), 210 (13), 197 (10), 126 (7), 101 (13), 69 (13), 57 (21), 43 (53), 32 (100).

*Synthesis of 1-((2,2-dimethyl)1,3-dioxolan-4-yl)-4-heptyl-1H-1,2,3-triazole (****4n****).* White solid, prepared in 85% yield from the reaction between non-1-yne (1.780 g, 14.4 mmol) and azide **3** (1.500 g, 9.60 mmol); TLC (ether-dichloromethane 10:1 v v^-1^); R_f_ = 0.68; m.p. 45−47 ºC; IR (ATR) –*v*_max_ /cm^−1^ 2954, 2919, 2851, 2360, 1380, 1320, 1150, 1030, 924; ^1^H NMR (300 MHz, CDCl_3_) *δ* 0.85 (t, 3H, *J* 9.0 Hz), 1.29 (sept, 8H, *J* 9.0 Hz), 1.33 (s, 3H), 1.35 (s, 3H), 1.64 (quint, 2H, *J* 9.0 Hz), 2.69 (t, 2H, *J* 9.0 Hz), 3.69 (dd, 1H, *J* 9.0, 6.0 Hz), 4.09 (dd, 1H, *J* 9.0, 6.0 Hz), 4.32 (dd, 1H, *J* 12.0, 6.0 Hz), 4.39−4.43 (m, 1H), 4.43 (dd, 1H, *J* 12.0, 6.0 Hz), 7.39 (s, 1H); ^13^C NMR (75 MHz, CDCl_3_) *δ* 14.0, 22.6, 22.2, 25.6, 26.6, 29.0, 29.1, 29.4, 31.7, 51.9, 66.4, 74.1, 110.0, 122.0, 148.5; MS (*m/z*, %) 281 ([M+], 13), 266 ([M − 15]+, 44), 223 (25), 210 (10), 197 (21), 166 (16), 152 (41), 138 (19), 124 (21), 110 (60), 101 (46), 82 (27), 68 (39), 57 (64), 43 (100), 30 (8).

*Synthesis of 1-((2,2-dimethyl-1,3-dioxolan-4-yl)methyl)-4-octyl-1H-1,2,3-triazole (****4o****).* White solid, prepared in 92% yield from the reaction between dec-1-yne (1.980 g, 14.4 mmol) and azide **3** (1.500 g, 9.60 mmol); TLC (ether-dichloromethane 10:1 v v^−1^); R_f_ = 0.69; m.p. 64−65 ºC; IR (ATR) –*v*_max_/cm^−1^ 2991, 2949, 2922, 2872, 2360, 1458, 1372, 1101, 844; ^1^H NMR (300 MHz, CDCl_3_) *δ* 0.85 (t, 3H, *J* 9.0 Hz), 1.25 (sept, 10H, *J* 9.0 Hz), 1.31 (s, 3H), 1.34 (s, 3H), 1.63 (quint, 2H, *J* 9.0 Hz), 2.68 (t, 2H, *J* 9.0 Hz), 3.70 (dd, 1H, *J* 9.0, 6.0 Hz), 4.08 (dd, 1H, *J* 9.0, 6.0 Hz), 4.36 (dd, 1H, *J* 12.0, 6.0 Hz), 4.35−4.45 (m, 1H), 4.49 (dd, 1H, *J* 12.0, 6.0 Hz), 7.47 (s, 1H); ^13^C NMR (75 MHz, CDCl_3_) *δ* 14.0, 22.6, 25.2, 25.6, 26.6, 29.1, 29.2, 29.3, 29.4, 31.8, 51.9, 66.4, 74.1, 110.0, 121.9, 148.4; MS (*m/z*, %) 295 ([M+], 15), 280 ([M − 15]+,48), 237 (19), 210 (9), 197 (27), 180 (14), 166 (40), 138 (21), 124 (25), 110 (68), 101 (50), 82 (29), 68 (40), 57 (67), 43 (100), 32 (93).

*Synthesis of 1-((2,2-dimethyl-1,3-dioxolan-4-yl)methyl)-4-nonyl-1H-1,2,3-triazole (****4p****).* White solid, prepared in 88% yield from the reaction between undec-1-yne (0.500 g, 3.28 mmol) and azide **3** (0.562 g, 3.62 mmol); TLC (ether-dichloromethane 10:1 v v^−1^); R_f_ = 0.69; m.p. 55−57 ºC; IR (ATR) –*v*_max_/cm^−1^ 2922, 2852, 2360, 1507, 1307, 1258, 1184, 1076, 845; ^1^H NMR (300 MHz, CDCl_3_) *δ* 0.85 (t, 3H, *J* 9.0 Hz), 1.25 (sext, 6H, *J* 9.0 Hz), 1.33 (s, 3H), 1.36 (s, 3H), 1.65 (quint, 2H, *J* 9.0 Hz), 2.69 (t, 2H, *J* 9.0 Hz), 3.71 (dd, 1H, *J* 9.0, 6.0 Hz), 4.09 (dd, 1H, *J* 9.0, 6.0 Hz), 4.36 (dd, 1H, *J* 12.0, 6.0 Hz), 4.42−4.45 (m, 1H), 4.46 (dd, 1H, *J* 12.0,6.0 Hz), 7.48 (s, 1H); ^13^C NMR (75 MHz, CDCl_3_) *δ* 14.0, 22.6, 25.2, 25.6, 26.6, 29.1, 29.2, 29.3, 29.4, 31.8, 51.9, 66.4, 74.1, 110.0, 121.9, 148.4; MS (*m/z*, %) 309 ([M+], 16), 294 ([M − 15]+, 47), 251 (14), 210 (11), 197 (32), 180 (37), 166 (15), 152 (16), 138 (23), 124 (27), 110 (75), 96 (43), 68 (41), 57 (68), 43 (100), 32 (29).

*Synthesis of 1-((2,2-dimethyl-1,3-dioxolan-4-yl)methyl)-4-decyl-1H-1,2,3-triazole (****4q****).* White solid, prepared in 94% yield from the reaction between dodec-1-yne (2.390 g, 14.4 mmol) and azide **3** (1.500 g, 9.60 mmol); TLC (ether-dichloromethane 10:1 v v^-1^); R_f_ = 0.53; m.p. 54−56 ºC; IR (ATR) –*v*_max_ /cm^−1^ 3063, 2954, 2916, 2848, 2360, 1682, 1558, 1540, 1269, 1063, 837; ^1^H NMR (300 MHz, CDCl_3_) *δ* 0.85 (t, 3H, *J* 9.0 Hz), 1.30 (sext, 14H, *J* 9.0 Hz), 1.32 (s, 3H), 1.35 (s, 3H), 1.64 (quint, 2H, *J* 9.0 Hz), 2.68 (t, 2H, *J* 9.0 Hz), 3.70 (dd, 1H, *J* 12.0, 6.0 Hz), 4.07 (dd, 1H, *J* 12.0, 6.0 Hz), 4.35 (dd, 1H, *J* 9.0, 6.0 Hz), 4.39−4.44 (m, 1H), 4.45 (dd, 1H, *J* 9.0, 6.0 Hz), 7.39 (s, 1H); ^13^C NMR (75 MHz, CDCl_3_) *δ* 14.0, 22.6, 25.2, 25.6, 26.6, 29.1, 29.2, 29.3, 29.4, 29.5, 29.5, 31.8, 51.9, 66.4, 74.1, 110.0, 121.9, 148.4; MS (*m/z*, %) 323 ([M+], 3), 308 ([M − 15]+, 9), 210 (2), 194 (7), 168 (2), 152 (3), 138 (5), 124 (6), 110 (19), 96 (11), 69 (18), 57 (17), 44 (34), 32 (100).

**3. The biological assay data of triazole derivatives (4a−4q)**

**
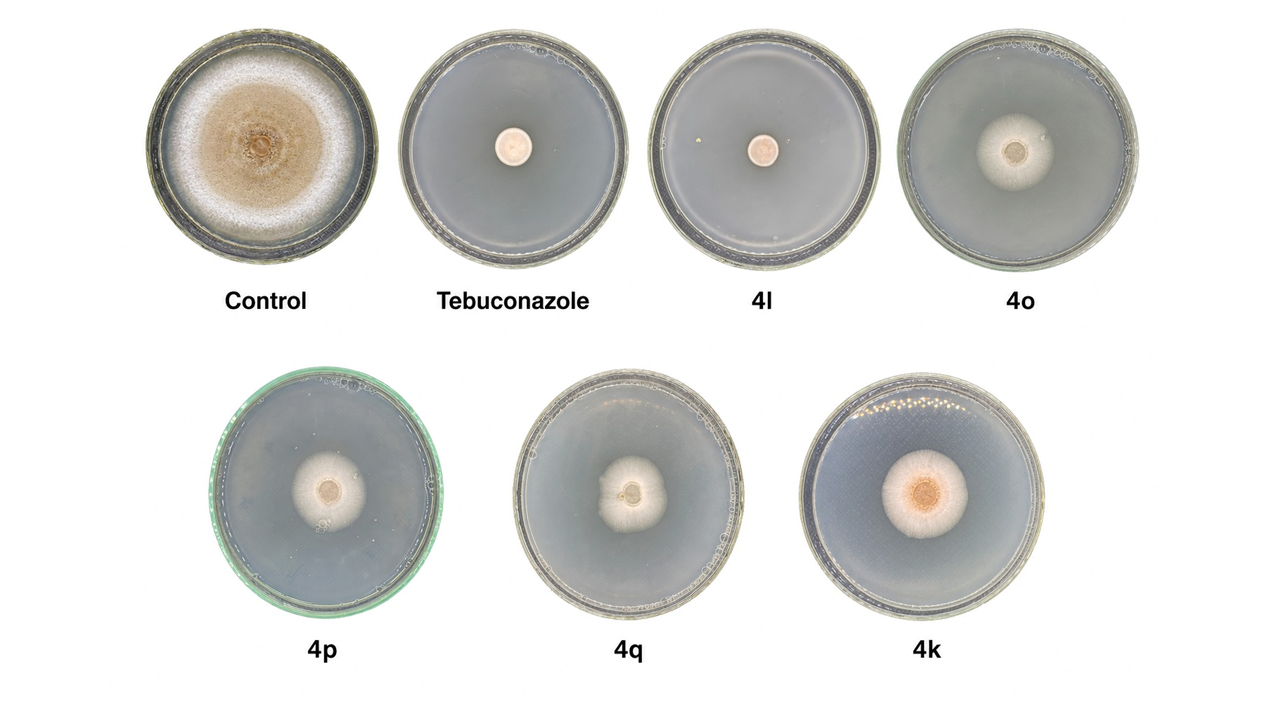
**

**Figure S81. Effects of glycerol-derived triazoles on the mycelial growth of *Neocosmospora falciformis***

**Supplementary Material – Table S1. ANOVA for mycelial growth diameter (cm) of *Neocosmospora falciformis* UENF/CF 295 at different concentrations of the tested compounds (n = 5).**

**Concentration: 1 µg/mL**

| Source of Variation | DF | SS | MS | F | p-value |
| --- | --- | --- | --- | --- | --- |
| Treatment | 17 | 3.284 | 0.193 | 1.027 | 0.447 ns |
| Residual | 72 | 13.548 | 0.188 | – | – |
| Total | 89 | 16.832 | – | – | – |

**Concentration: 10 µg/mL**

| Source of Variation | DF | SS | MS | F | p-value |
| --- | --- | --- | --- | --- | --- |
| Treatment | 17 | 6.912 | 0.407 | 2.174 | 0.020 * |
| Residual | 72 | 13.484 | 0.187 | – | – |
| Total | 89 | 20.396 | – | – | – |

**Concentration: 100 µg/mL**

| Source of Variation | DF | SS | MS | F | p-value |
| --- | --- | --- | --- | --- | --- |
| Treatment | 17 | 49.873 | 2.934 | 13.674 | < 0.001 *** |
| Residual | 72 | 15.452 | 0.215 | – | – |
| Total | 89 | 65.325 | – | – | – |

**Concentration: 500 µg/mL**

| Source of Variation | DF | SS | MS | F | p-value |
| --- | --- | --- | --- | --- | --- |
| Treatment | 17 | 160.245 | 9.426 | 44.936 | < 0.001 *** |
| Residual | 72 | 15.108 | 0.210 | – | – |
| Total | 89 | 175.353 | – | – | – |

**Concentration: 1000 µg/mL**

| Source of Variation | DF | SS | MS | F | p-value |
| --- | --- | --- | --- | --- | --- |
| Treatment | 17 | 212.388 | 12.493 | 84.278 | < 0.001 *** |
| Residual | 72 | 10.672 | 0.148 | – | – |
| Total | 89 | 223.060 | – | – | – |

**Legend for Table S1**

- **DF**: degrees of freedom
- **SS**: sum of squares
- **MS**: mean square
- **F**: F-statistic
- **p-value**: statistical significance
- **ns**: not significant (p > 0.05)
- *** p < 0.001; ** p < 0.01; * p < 0.05

Five replicates per treatment (n = 5). Data met assumptions of homogeneity of variances (Bartlett’s test) and normality of residuals (Shapiro–Wilk test). Means were grouped by the Scott-Knott test at 5% significance, as shown in Table 1 of the main article.

**Supplementary Material – Table S2. ANOVA for the average number of spores produced by *Neocosmospora falciformis* UENF/CF 295 at different concentrations of the tested compounds (n = 5).**

**Concentration: 1 µg/mL**

| Source of Variation | DF | SS | MS | F | p-value |
| --- | --- | --- | --- | --- | --- |
| Treatment | 17 | 14,872.3 | 874.84 | 4.62 | < 0.001 *** |
| Residual | 72 | 13,634.7 | 189.37 | – | – |
| Total | 89 | 28,507.0 | – | – | – |

**Concentration: 10 µg/mL**

| Source of Variation | DF | SS | MS | F | p-value |
| --- | --- | --- | --- | --- | --- |
| Treatment | 17 | 62,847.5 | 3,696.9 | 18.73 | < 0.001 *** |
| Residual | 72 | 14,212.6 | 197.40 | – | – |
| Total | 89 | 77,060.1 | – | – | – |

**Concentration: 100 µg/mL**

| Source of Variation | DF | SS | MS | F | p-value |
| --- | --- | --- | --- | --- | --- |
| Treatment | 17 | 97,917.3 | 5,759.8 | 29.21 | < 0.001 *** |
| Residual | 72 | 14,197.2 | 197.18 | – | – |
| Total | 89 | 112,114.5 | – | – | – |

**Concentration: 500 µg/mL**

| Source of Variation | DF | SS | MS | F | p-value |
| --- | --- | --- | --- | --- | --- |
| Treatment | 17 | 154,839.6 | 9,108.2 | 48.12 | < 0.001 *** |
| Residual | 72 | 13,628.3 | 189.28 | – | – |
| Total | 89 | 168,467.9 | – | – | – |

**Concentration: 1000 µg/mL**

| Source of Variation | DF | SS | MS | F | p-value |
| --- | --- | --- | --- | --- | --- |
| Treatment | 17 | 558,681.1 | 32,863.6 | 172.87 | < 0.001 *** |
| Residual | 72 | 13,686.2 | 190.09 | – | – |
| Total | 89 | 572,367.3 | – | – | – |

**Legend for Table S2**

- **DF**: degrees of freedom
- **SS**: sum of squares
- **MS**: mean square
- **F**: F-statistic
- **p-value**: statistical significance
- *** p < 0.001

Five replicates per treatment (n = 5). Data met assumptions of homogeneity of variances (Bartlett’s test) and normality of residuals (Shapiro–Wilk test). Means were grouped by the Scott-Knott test at 5% significance, as shown in Table 3 of the main article.
